# Text Analysis of Trends in Health Equity and Disparities From the Internal Revenue Service Tax Documentation Submitted by US Nonprofit Hospitals Between 2010 and 2019: Exploratory Study

**DOI:** 10.2196/44330

**Published:** 2023-05-24

**Authors:** Emily Hadley, Laura Haak Marcial, Wes Quattrone, Georgiy Bobashev

**Affiliations:** 1 RTI International Durham, NC United States

**Keywords:** text mining, natural language processing, health care disparities, hospital administration

## Abstract

**Background:**

Many US hospitals are classified as nonprofits and receive tax-exempt status partially in exchange for providing benefits to the community. Proof of compliance is collected with the Schedule H form submitted as part of the annual Internal Revenue Service Form 990 (F990H), including a free-response text section that is known for being ambiguous and difficult to audit. This research is among the first to use natural language processing approaches to evaluate this text section with a focus on health equity and disparities.

**Objective:**

This study aims to determine the extent to which the free-response text in F990H reveals how nonprofit hospitals address health equity and disparities, including alignment with public priorities.

**Methods:**

We used free-response text submitted by hospital reporting entities in Part V and VI of the Internal Revenue Service Form 990 Schedule H between 2010 and 2019. We identified 29 main themes connected to health equity and disparities, and 152 related key phrases. We tallied occurrences of these phrases through term frequency analysis, calculated the Moran I statistic to assess geographic variation in 2018, analyzed Google Trends use for the same terms during the same period, and used semantic search with Sentence-BERT in Python to understand contextual use.

**Results:**

We found increased use from 2010 to 2019 across all the 29 phrase themes related to health equity and disparities. More than 90% of hospital reporting entities used terms in 2018 and 2019 related to affordability (2018: 2117/2131, 99.34%; 2019: 1620/1627, 99.57%), government organizations (2018: 2053/2131, 96.33%; 2019: 1577/1627, 96.93%), mental health (2018: 1937/2131, 90.9%; 2019: 1517/1627, 93.24%), and data collection (2018: 1947/2131, 91.37%; 2019: 1502/1627, 92.32%). The themes with the largest relative increase were *LGBTQ* (lesbian, gay, bisexual, transgender, and queer; 1676%; 2010: 12/2328, 0.51%; 2019: 149/1627, 9.16%) and *social determinants of health* (958%; 2010: 68/2328, 2.92%; 2019: 503/1627, 30.92%). Terms related to *homelessness* varied geographically from 2010 to 2018, and terms related to *equity*, *health IT*, *immigration*, *LGBTQ, oral health*, *rural*, *social determinants of health*, and *substance use* showed statistically significant (*P*<.05) geographic variation in 2018. The largest percentage point increase was for terms related to substance use (2010: 403/2328, 17.31%; 2019: 1149/1627, 70.62%). However, use in themes such as *LGBTQ*, *disability, oral health,* and *race and ethnicity* ranked lower than public interest in these topics, and some increased mentions of themes were to explicitly say that no action was taken.

**Conclusions:**

Hospital reporting entities demonstrate an increasing awareness of health equity and disparities in community benefit tax documentation, but these do not necessarily correspond with general population interests or additional action. We propose further investigation of alignment with community health needs assessments and make suggestions for improvements to F990H reporting requirements.

## Introduction

### Background

Nonprofit hospitals in the United States are exempt from federal taxes. In exchange for this exemption, these hospitals have an obligation to provide community benefit [1]. The proof of compliance is collected with Schedule H, a form submitted as part of the annual Form 990 (F990) Internal Revenue Service (IRS) tax documentation for nonprofit hospitals. A substantial section of the F990 Schedule H (F990H) is composed of free-response (unstructured) text fields, where reporting entities can voluntarily provide details on community benefit spending. This may include discussion of community needs and the measures a hospital has or has not taken to address these needs.

Community needs can and do include topics related to health equity and disparities. Health equity is commonly understood as an opportunity for all individuals to be healthy, regardless of membership in a group that has historically been economically or socially disadvantaged [2]. Health disparities are defined as a particular type of health difference that is worse among socially disadvantaged individuals, namely members of disadvantaged race or ethnicity groups, or economically disadvantaged people within any racial or ethnic group [2]. Addressing social determinants of health (SDOH), defined as the economic and social conditions that impact the health of people and communities, is considered a primary approach for reducing health disparities and achieving health equity [3].

In recent years, legislators and other stakeholders have paid increasing attention to whether hospitals are providing adequate community benefits to justify their tax-exempt status [4]. The IRS is required to review each tax-exempt hospital’s community benefit activities at least once every 3 years, although historically, this requirement has been ambiguous and difficult to track [5]. In 2020, the Government Accountability Office completed a review of the IRS’s implementation of requirements for tax-exempt hospitals and made a series of recommendations [5]. One recommendation was that the IRS establish a well-documented process for identifying hospitals at risk of noncompliance with the community benefit standard; the IRS added instructions in April and July 2021 for employees to document case files with relevant facts and circumstances considered during their review to determine whether the hospital organization satisfied the community benefit standard [5]. One unfulfilled recommendation is updating F990H to ensure that the community benefit a hospital is providing is clear and can be easily identified by Congress and the public [5]. The IRS recognizes that 3 of the factors currently addressed through open-ended narrative responses are not part of the quantitative, machine-readable files and that a revised F990H could more clearly, consistently, and comprehensively provide community benefit information to the public [5]. A related open recommendation is that Congress should specify which hospital services and activities are sufficient for community benefit [5]. These recommendations provide an opportunity for an explicit alignment with approaches to address health equity and disparities.

Most existing studies that use data from F990H have focused on financial data. Empirical studies suggest that nonprofit hospital community benefit spending focuses on charity care and patient care services with little effort to improve community health [6-8]. Limited literature has explored community benefit spending with a focus on health equity or disparities, generally finding that increased IRS reporting clarification or explicit goals to address health disparities could more directly address community needs [9-11]. The text in F990H is unstructured data that varies in detail and length and has been historically challenging to analyze or review in large quantities. Only 1 study by Chen et al [12] reviewed this text in depth using a manual review of a small sample of 47 hospitals from 2015 to 2017. Recent advances in natural language processing techniques have made text analysis with much larger and longer text data sets more accessible [13].

### Objective

We present a novel text analysis of F990H tax documentation to understand if and how US nonprofit hospitals address health equity and disparities through community benefit programming. Our research is the first known work to analyze the F990H free-response text on a national scale across a 9-year period using text analytics approaches. We contribute to a larger body of work regarding hospital community benefits programming, including the limited existing discussion on how hospitals use community benefits programming to address health equity and disparities. By comparing our results with public search trends and identifying gaps in term use and action, we provide findings that stakeholders can use to advocate for community benefit approaches and improvements to F990H to better address health equity and disparities.

## Methods

### Data Source

The data for this analysis come from free-response text in Part V and VI of Schedule H from F990s submitted by US nonprofit hospitals for tax years between 2010 and 2019 [14]. Detailed descriptions of the specific IRS requirements for Parts V and VI are included in Multimedia Appendix 1. Data from 2020 onward were not available at the time of analysis because of a data lag that has been exacerbated by the COVID-19 pandemic. F990H is submitted annually (although sometimes delayed by extensions) by a hospital facility or, in many cases, by a hospital organization with a shared employee identification number for multiple hospital facilities. All free-response answers for Part V and VI were combined for this analysis. Data were collected and maintained through the Community Benefit Insight project [15]. Analysis was completed in Python (Python Software Foundation) using pandas, numpy, nltk, PyTorch, and SentenceTransformers. Visualizations were created in Tableau (Tableau) and R (R Foundation for Statistical Computing) using ggplot2.

The free-response text sections include answers to several questions regarding community health needs assessments (CHNAs), financial eligibility assistance programs, and descriptions of whether and how identified community needs are addressed by a hospital facility. With a few exceptions, these questions are often ambiguous, and hospitals voluntarily choose the level of detail they provide [16]. The F990H for each hospital is reviewed by the IRS at least once every 3 years but rarely audited; an audit is more complicated and thorough than a review. Even if audited, there is no clear definition of the activities and services that are sufficient to prove community benefit [5]. Text responses are generally full sentences and paragraphs. Colloquial terminology and misspellings are infrequent.

**Figure 1:**
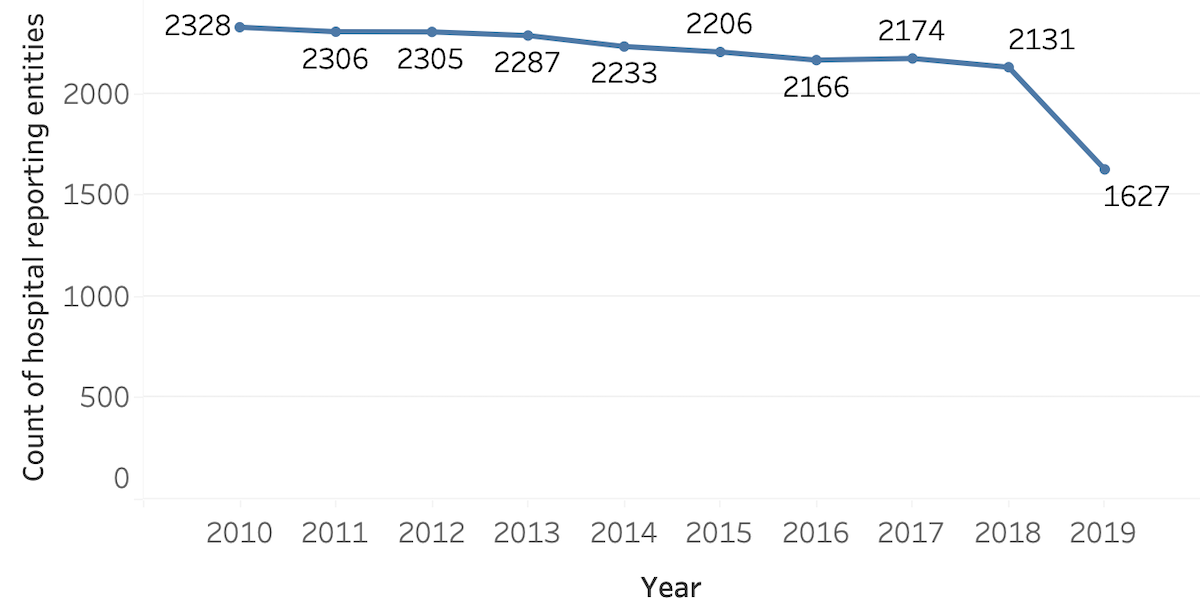
Count of hospital reporting entities from 2010 to 2019. The number of unique hospital reporting entities with free-response text data in Schedule H each year from 2010 to 2019.

Figure 1 shows the number of hospital entities that report each year. As portions of the free-response text are required in the IRS Schedule H Instructions (Multimedia Appendix 1), every nonprofit hospital reporting entity is expected to have a free-response text entry; an average of 99.9% of hospital reporting entities that submitted the IRS Form 990 have completed the free-response text in Schedule H. Of the 2131 reporting hospital entities in 2018, a total of 90.5% (n=1930) had continuously submitted free-response text data since 2010. However, the overall number of hospital reporting entities decreased from 2010 to 2018, likely reflecting national trends in hospital consolidation and closure [17,18]. The number of reporting hospitals was notably low in 2019 (n=1627), likely because of the reporting extensions permitted in 2020 during the COVID-19 pandemic. Though we anticipate that 2019 is likely missing data from some hospitals, we decided to retain the 2019 data in the analysis as we prioritized the timeliness of the findings. The median number of words in text responses increased from 1629 in 2010 to 3439 in 2019, whereas the average number of words increased from 2840 in 2010 to 10,123 in 2019. The average was skewed by hospital reporting entities in California, Arizona, and Utah, which submitted average responses of over 30,000 words. Most hospitals (2406/2554, 94.21%) do not submit duplicate text across years.

### Outcomes and Variable Construction

Term frequency analysis is a type of lexical analysis that searches for an exact word or phrase using a bag-of-words model [19]. In this analysis, we used the term frequency to flag whether a word or phrase was used one or more times by a particular hospital organization in each tax year. Term frequency analysis was proposed as an option in the first step of computational grounded theory in sociology, which combines expert human knowledge with the processing power and pattern recognition of computers for content analysis [20]. This study leveraged an opportunity to use clearly defined expectations from stakeholders that did not align with the more exploratory nature of the computational grounded theory approach. However, we used the key principles of computational grounded theory by deriving a list of terms, regularly seeking expert feedback, and validating our results. We also evaluated a semisupervised topic modeling approach that could have supported finding additional terms and topics related to key anchor words; however, we found that the suggested topics were too broad or included terms that were unrelated to the topic, as defined by stakeholders [21,22]. Improvements in semisupervised topic modeling in this context could be an area of future research.

Term frequency analysis requires a list of words or phrases to begin with. A limitation of term frequency analysis is that it will only consider the exact words and phrases searched for, so a thorough and nontrivial multistep process combining text analysis approaches and stakeholder input was used to create the term set. We used a 3-step process to build this list.

The first step in key term selection was providing stakeholders the opportunity to suggest specific words, phrases, and themes related to health equity and disparities. Stakeholders included 11 subject matter experts and programming staff with experience in health and community benefits from the Robert Wood Johnson Foundation and the Robert Wood Johnson Foundation grantees Community Catalyst and Healthy Food in Health Care. These stakeholders provided suggestions and agreed on words, phrases, and themes in meetings and by email from fall 2021 to fall 2022. The terms included both single words (*rural*) and phrases (*data collection*). Terms with singular and specific meanings were selected. For example, specific drugs such as *opioids* and *fentanyl* were used for the *substance use* theme, as opposed to *drug*, which is broader and not always related to substance use. In some cases, both singular and plural versions of a term were included (ie, *equity* and *equities*).

The second step involved n-gram tokenization of the text, followed by a search for the 1000 most common single words, bigrams, and trigrams. We reviewed the most common words and phrases. Common terms closely related to the themes suggested by the stakeholders were added to the full list of terms. For example, the terms *listening tour* and *focus group* were both added to the *data collection* theme through this process. The third step involved a review of the SDOH literature associated with the Healthy People 2030 initiative led by the US Office of Disease Prevention and Health Promotion for any other words or phrases that should be included [23]. For example, this review led to the addition of *lead-based paint* and *air pollution* to the environment theme. The final step was to provide the list of terms to a variety of stakeholders, including those from the first step, for feedback.

**Figure 2:**
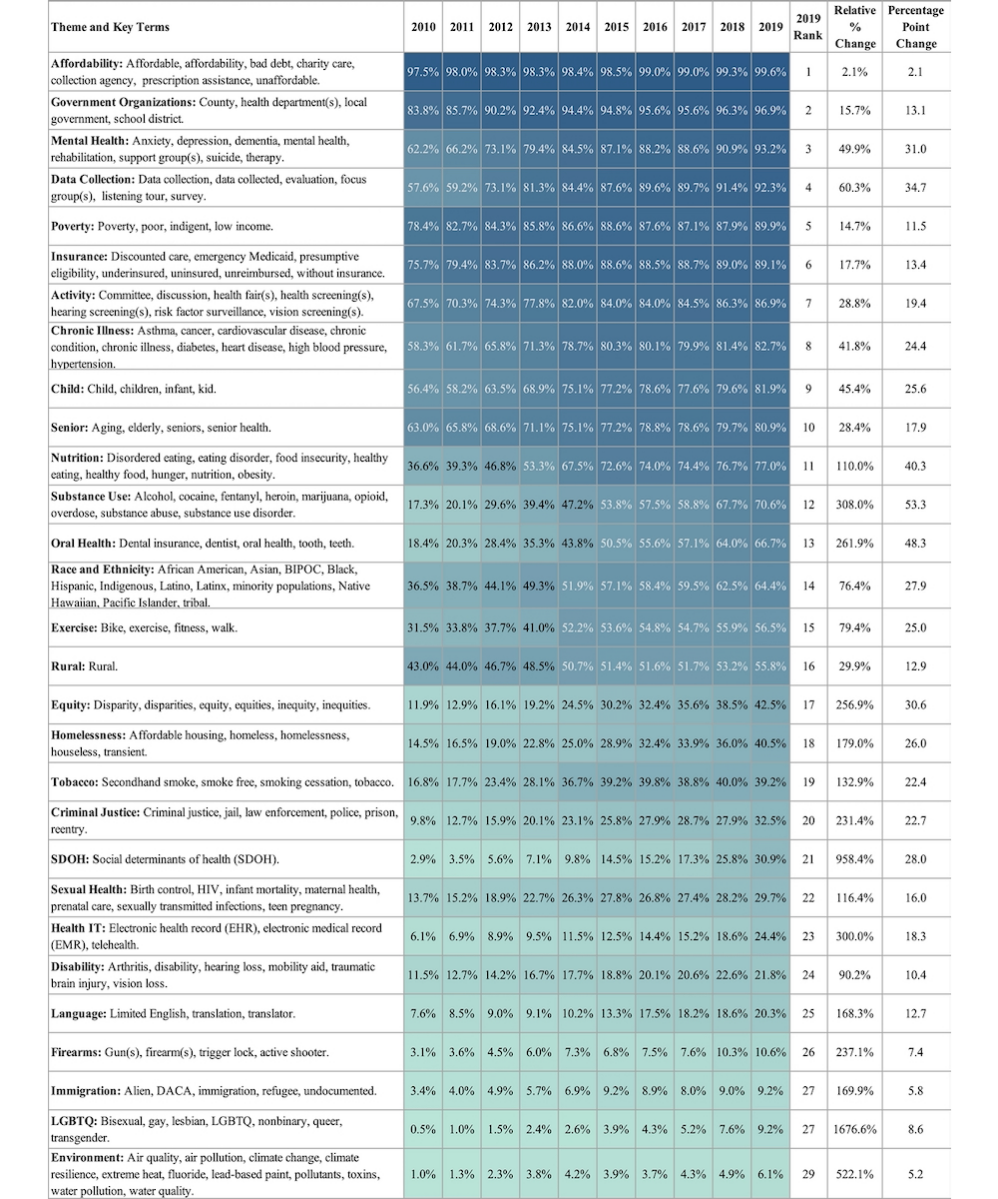
Percent of hospital reporting entities with one or more uses of term in theme by tax year, ranked by use in 2019. This heat map highlights the number of hospital reporting entities with one or more uses of any term from a health equity or disparity theme by tax year. Darker tones indicate a higher use. The figure is sorted by ranking of use in the 2019 tax year. The figure also includes the relative percentage and percentage point changes from 2010 to 2019. BIPOC: Black, Indigenous, (and) People of Color; DACA: Deferred Action for Childhood Arrivals; LGBTQ: lesbian, gay, bisexual, transgender, and queer.

The resulting term set spanned 29 themes and included 152 words and phrases. These words and themes are shown in Figure 2. A clean version of the text was created such that it was all lower case with no symbols or punctuation.

Term frequency analysis was performed using this text. We ranked the use of these terms in 2019. We also calculated the percentage and percentage point change from 2010 to 2019. Percent change measurements are useful for understanding the relative increase in term use, especially for less-frequent themes. Percentage point change measurements are useful for understanding the raw change in term use.

### Geographic Variation in F990H Use

Community benefit programming is intended to align with community needs, which may vary by geographic region. To assess the alignment of F990H theme use by geography, we first aggregated the percentage of hospital reporting entities with one or more uses of a term by state for each year from 2010 to 2019 and mapped the findings. We visually reviewed the maps for trends in changes over time. We then calculated the Moran I statistic for 2018 to evaluate the presence of spatial autocorrelation to determine if there is a pattern of similarity between observations that are geographically close to one another [24]. The data from 2018 were used as it is the most recent year with the most complete data. Neighboring states were identified through centroids up to 1000 km apart. We performed a 1-sided statistical significance test with an alternative hypothesis that the observed spatial autocorrelation in the data was significantly greater than what would be expected by chance under a null hypothesis of no spatial autocorrelation. Statistical significance was assessed at α values of .001, .01, and .05.

### Comparison With Google Trends

One criticism of hospital community benefits programming (and related spending) is that it is out of touch with community-identified needs [8]. Google Trends has emerged as a source for investigating how social trends change over time [25]. We used Google Trends as a proxy for the general population’s interest in topics related to health equity and disparities. We obtained the relative frequency of term use in Google searches related to health (as categorized by Google) between January 1, 2010, and December 31, 2019, in the United States, for the words and phrases in the term list. We note a limitation in that it is unclear how Google determines the category to which a search query belongs, and we are unable to determine the scope of the health category; however, for the purposes of this work, filtering to the health category is still preferable to using all search categories. Further details on Google Trends are provided in Multimedia Appendix 2.

Considering only the searches in 2019, we ranked public interest in each of the 29 themes. We compared this Google Trends ranking with the ranking of use in F990H in 2019. We assigned similar relative usage to themes within 5 rankings of each other (eg, *Nutrition* has rank 11 in 2019 F990H use and rank 10 in 2019 in Google Trends searches; *Nutrition* is considered a theme with “similar relative usage” as rank 11 is only 1 rank difference from rank 10). A difference of 6 to 18 rankings was considered large, whereas a difference of more than 18 rankings was considered very large. These thresholds were selected to ensure bands of similar width in the figure were used for comparison of the Google Trends and F990H results.

### Semantic Search

A major criticism of term frequency analysis is that, although it is useful for determining whether a keyword or phrase is used, it is challenging to determine the context or meaning of the term. Therefore, a semantic search was used to augment term frequency analysis. This methodology is used by major search engines to search for meaning by evaluating both the searcher’s intent and the contextual meaning of the terms. We used the question-answer retrieval implementation of Sentence-BERT, a model pretrained on the Natural Questions data set which uses real questions from Google Search with annotated data from Wikipedia as the answers [26]. This approach is best for an asymmetrical search task in which a short query (such as a question or keyword) is used to find a sentence or paragraph.

For this project, we built a semantic search model and used two search queries for each theme: (1) “took action on <theme>” and (2) “did not take action on <theme>.”

We reviewed the top 20 sentences returned for each query and reported summary findings from the themes with the 3 largest percent or percentage point increase. We selected up to 3 sentences for each theme that best reflected the dichotomy between taking action and not taking action. Not all themes had examples of both action and inaction, and we intentionally did not seek to quantify the results from the semantic search, as it is an imperfect method that can return ambiguous or unrelated sentences.

### Ethical Considerations

This research was completed using publicly available secondary data from hospital reporting entities and did not require institutional review board review because it did not involve human participants. All research was conducted with an ethic of respect for cultures, communities, individuals, and independent knowledge. Feedback was obtained from stakeholders likely to be impacted by the findings of this study.

## Results

### Overview

Figure 2 shows the results of the term frequency analysis sorted by prevalence in 2019. Figures S1-S29 in Multimedia Appendix 3 provide the detailed disaggregation for each theme. Figure 2 illustrates that nearly every hospital organization uses a term related to *affordability* (2018: 2117/2131, 99.34%; 2019: 1620/1627, 99.57%), and more than 90% of hospital reporting entities used a term in 2018 and 2019 related to *government organizations* (2018: 2053/2131, 96.33%; 2019: 1577/1627, 96.93%), *mental health* (2018: 1937/2131, 90.9%; 2019: 1517/1627, 93.24%), and *data collection* (2018: 1947/2131, 91.37%; 2019: 1502/1627, 92.32%).

The least used themes, with a prevalence of less than 10% in 2018 and 2019, were related to immigration (2018: 191/2131, 8.96%; 2019: 149/1627, 9.16%), LGTBQ (2018: 161/2131, 7.56%; 2019: 149/1627, 9.16%), and the environment (2018: 104/2131, 4.88%; 2019: 100/1627, 6.14%).

Figure 2 provides additional details on the percentage increase and raw percentage increase in the use of a term at least once from 2010 to 2019. All 29 themes showed an increase in use in Schedule H of F990, as indicated by both relative and percentage point change. Although LGBTQ (lesbian, gay, bisexual, transgender, and queer)-related terms were used by only a small percentage of hospital reporting entities (Figure 2), this theme saw the largest relative increase from 2010 to 2019 (1676%; 2010: 12/2328, 0.51%; 2019: 149/1627, 9.16%). Other themes with large relative increases were *SDOH* (958%; 2010: 68/2328, 2.92%; 2019: 503/1627, 30.92%) and *environment* (522%; 2010: 23/2328, 0.99%; 2019: 100/1627, 6.15%). The themes with the smallest relative increase include *affordability* (2.06%; 2010: 2270/2328, 97.51%; 2019: 1620/1627, 99.57%) and *insurance* (13.39%; 2010: 1763/2328, 75.73%; 2019: 1450/1627, 89.12%). Terms related to *substance use* saw the largest raw percentage point increase: less than a fifth of hospital reporting entities used any *substance use* language in 2010 (403/2328, 17.31%), and more than two-thirds of hospital reporting entities used a *substance use* term in 2019 (1149/1627, 70.62%). Other themes with notable increases included keywords related to *oral health* (48.26 percentage points, 2010: 429/2328, 18.43%; 2019: 1085/1627, 66.69%) and *nutrition* (40.31 percentage points, 2010: 853/2328, 36.64%; 2019: 1252/1627, 76.95%). Themes with the smallest percentage point increase included *affordability* (2.06 percentage points, 2010: 2270/2328, 97.51%; 2019: 1620/1627, 99.57%) and *environment* (5.16 percentage points, 2010: 23/2328, 0.99%; 2019: 100/1627, 6.15%).

### Geographic Variation in Themes From 2010 to 2019

**Figure 3:**
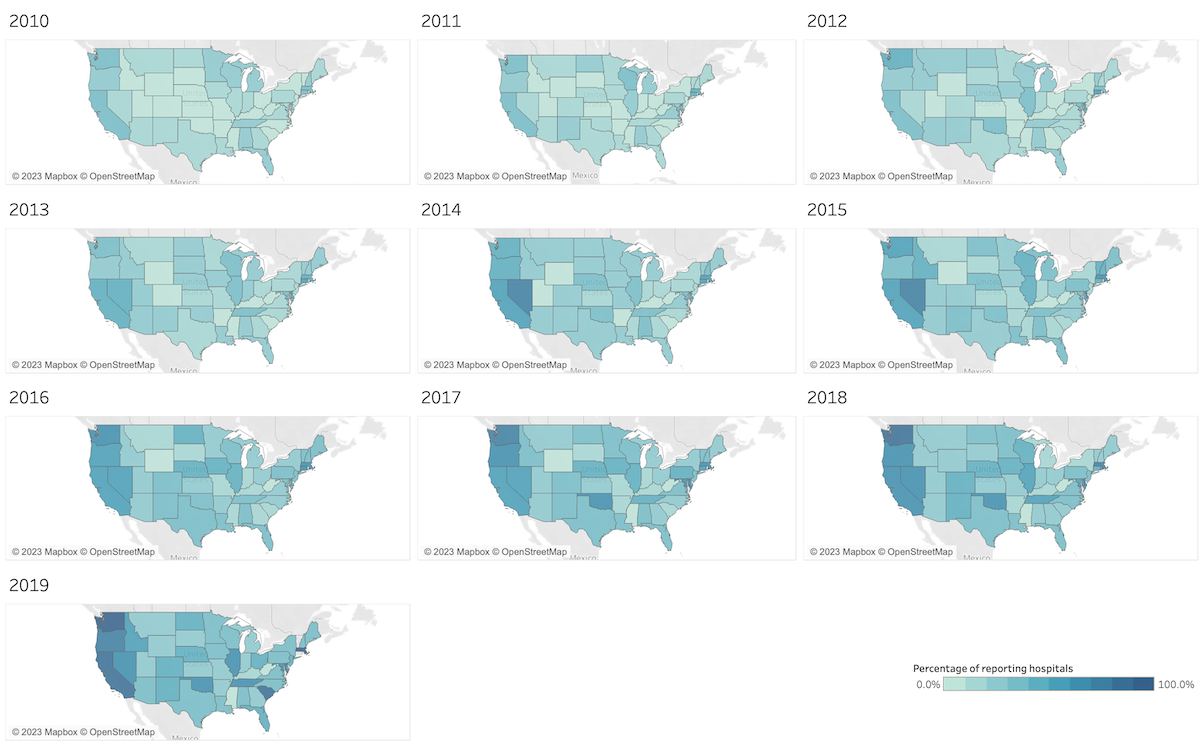
Geographic variation from 2010 to 2019 of the percentage of hospitals in each state using one or more key terms related to homelessness. A darker color indicates a higher use.

Theme use can vary across states and time. In Figure 3, we highlight the theme with the most visually clear example of change in geographic variability, *homelessness*. In 2010, the percentage of hospital reporting entities using one or more key terms related to *homelessness* was small and similar across states. From 2012 to 2015, the proportion of hospital reporting entities using one or more key terms related to *homelessness* increased in states on the West Coast. In 2018 and 2019, the majority of hospital reporting entities on the West Coast (2018: 99/168, 58.9%; 2019: 115/161, 71.4%) used one or more terms related to *homelessness*. The geographic maps for all the themes are available in Multimedia Appendix 4.

Although homelessness was the most visually striking theme for showing change across time, Table 1 highlights the results of the Moran test for spatial autocorrelation among themes in 2018. A total of 8 themes showed statistically significant (*equity*: *P*=.001; *health IT*: *P*=.02; *immigration*: *P*=.002; *LGBTQ*: *P*=.007; *oral health*: *P*=.04; *rural*: *P*<.001; *SDOH*: *P*<.001; *substance use*: *P*=.003) positive spatial autocorrelation, suggesting some degree of neighboring state clustering in the discussion of themes in F990H. The maps in Multimedia Appendix 4 help illustrate where clusters may occur. The *equity* theme was used in clusters of states on the East and West coasts, with less use in the Midwest, excluding a small cluster around Indiana. The *health IT* theme had higher use in a cluster of neighboring states, including Nebraska, South Dakota, Colorado, Montana, Utah, and Iowa as well as another cluster, including Indiana, Michigan, Ohio, Kentucky, and Illinois. The use of the *immigration* theme was clustered in the Pacific Northwest (Washington, Idaho, and Oregon) and a smaller cluster in the Northeast (Massachusetts, New York, New Jersey, and Connecticut). The *LGBTQ* theme was almost exclusively used in 3 clusters: West Coast (Washington, Oregon, and California), Midwest (Minnesota, Wisconsin, Illinois, Kentucky, and Oklahoma), and New England (Maine, Vermont, Massachuetts, and Connecticut), with some mention in New York and New Jersey. The *oral health* theme had a number of clusters, including Mountain West (Idaho, Montana, Wyoming, and Colorado), mid-Atlantic (Pennsylvania, New Jersey, Maryland, and Virginia), and New England (New Hampshire, Vermont, Massachusetts, Connecticut, and Rhode Island). The use of *rural* was higher in the Midwest (Wisconsin, Minnesota, Iowa, Missouri, Arkansas, Oklahoma, Kansas, North Dakota, and South Dakota) and Mountain West (Montana, Idaho, Utah, Colordado, and Oregon). The use of terms in the *SDOH* theme was higher in the West Coast (Washington, Oregon, and California) and North Atlantic (Maine, Massachusetts, New York, Rhode Island, New Jersey, and Maryland). Finally, the *substance use* theme had clusters in the Southwest (Utah, Arizona, New Mexico, and Colorado), Midwest (Wisoconsin, Ilinois, Missouri, Iowa, Ohio, Kentucky, and Tennessee) and on the East Coast (excluding North Carolina and Georgia).

**Table 1:** Results from Moran test for spatial autocorrelation among states by theme in 2018.

Theme	Moran I statistic	Variance	*P* value
Activities	0.006	0.002	.26
Affordability	0.035	0.003	.12
Child	–0.048	0.003	.70
Chronic illness	–0.006	0.003	.38
Criminal justice	0.047	0.003	.09
Data collection	–0.036	0.003	.61
Disability	0.012	0.003	.26
Environment	–0.019	0.003	.48
Equity	0.149	0.003	.001
Exercise	–0.036	0.003	.61
Firearms	–0.03	0.003	.56
Government organizations	–0.021	0.002	.50
Health IT	0.082	0.003	.02
Homelessness	0.055	0.003	.07
Immigration	0.118	0.002	.002
Insurance	–0.008	0.003	.40
Language	–0.049	0.003	.70
LGBTQ^a^	0.099	0.002	.007
Mental health	0.011	0.003	.26
Nutrition	–0.062	0.003	.78
Oral health	0.066	0.003	.04
Poverty	–0.03	0.003	.56
Race and ethnicity	0.038	0.003	.12
Rural	0.179	0.003	<.001
SDOH^b^	0.208	0.003	<.001
Senior	0.059	0.003	.05
Sexual health	0.063	0.003	.05
Substance use	0.12	0.003	.003
Tobacco	0.037	0.003	.12

^a^LGBTQ: lesbian, gay, bisexual, transgender, and queer.

^b^SDOH: social determinants of health.

### Google Trends

**Figure 4:**
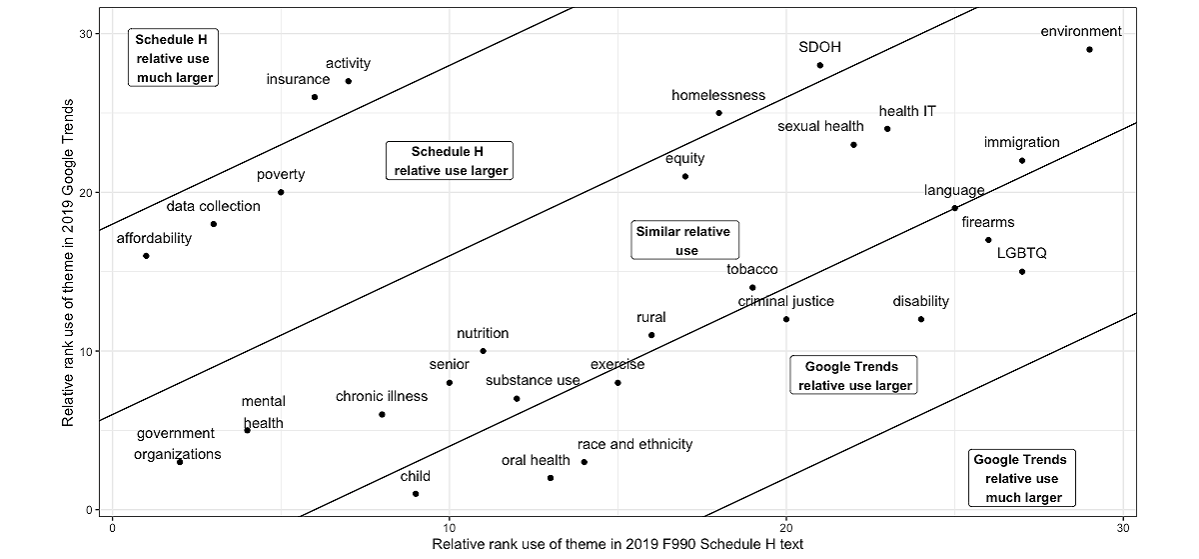
Relative rank of themes in 2019 in Google Trends versus F990 Schedule H. This figure considers the relative rank of a health equity or disparity theme in Google Trends versus F990H tax documentation in 2019. The figure is segmented into bands that indicate when Google Trends’ relative use in 2019 is larger than F990H’s relative use, relative use is similar, or relative use in F990H is larger than in Google Trends. LGBTQ: lesbian, gay, bisexual, transgender, and queer; SDOH: social determinants of health.

Figure 4 shows the relative rank of use of a theme in hospital F990H reporting versus Google Trends in 2019. A rank of 1 was the most used, whereas a rank of 29 was the least used. Items in the middle band (eg, *government organizations*, *chronic illness*, and *immigration*) reflect similar relative use. Items in the bands near the top-left corner of the figure (eg, *activity and insurance*) reflect themes where the relative rank in Schedule H reporting is higher than that in Google Trends. Items in the bands toward the bottom-right corner of the figure (eg, *LGBTQ*, *oral health, and disability*) reflect themes where the relative rank in Google Trends is higher than in Schedule H. A total of 2 themes (*government organizations* and *mental health*) ranked in the top 5 and 1 theme (*environment*) ranked in the bottom 5 for relative use in both Google Trends and Schedule H in 2019.

**Figure 5:**
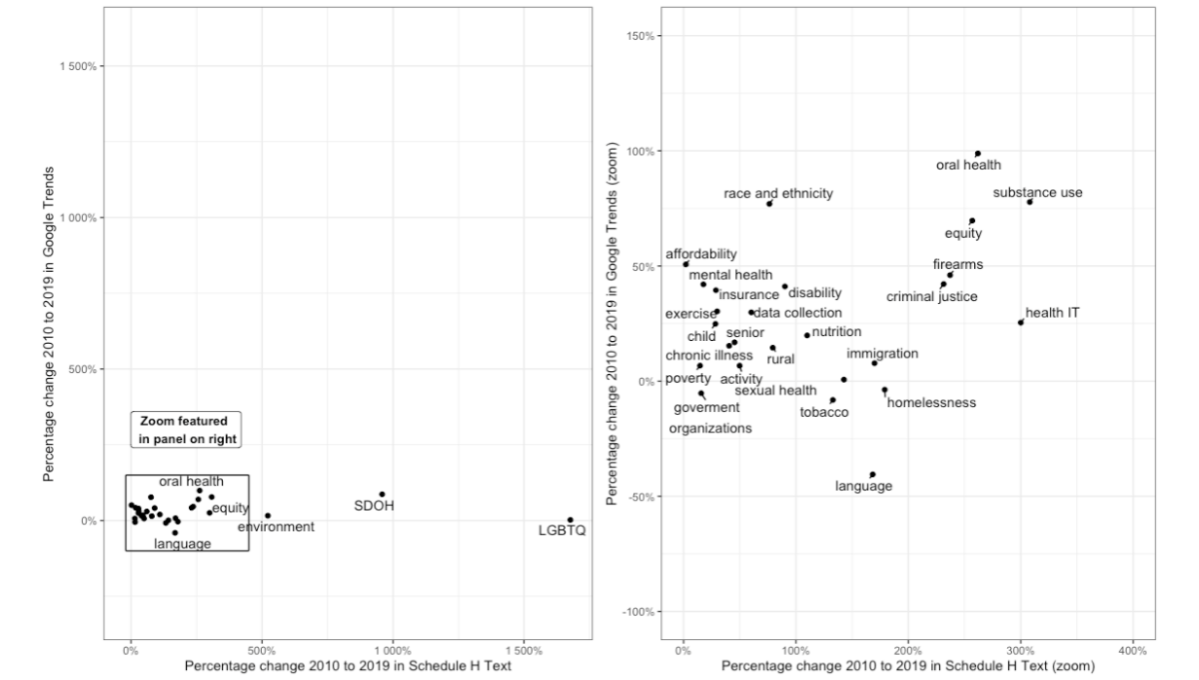
Percentage change in relative use of theme from 2010 to 2019 in Google Trends versus in F990 Schedule H. This plot compares the percentage change in the relative use of a health equity or disparity theme in Google Trends with the percentage change in relative use in F990 Schedule H forms from 2010 to 2019. The left panel shows all 29 themes. The right panel shows the 26 themes. LGBTQ: lesbian, gay, bisexual, transgender, and queer; SDOH: social determinants of health.

Figure 5 highlights the percentage change in relative use from 2010 to 2019 by hospitals for Google Trends versus F990H. Although the *LGBTQ* theme has greater use in Google Trends in Figure 4, this theme saw a much larger relative increase in use in the analysis timeframe in F990H. The *SDOH* and *environment* themes also increased considerably in use in F990H and saw some increase in use in Google Trends. The right panel in Figure 4 highlights that many themes had small and sometimes negative changes in Google Trends, even though the theme saw a substantial increase in use in F990H. *Oral health* and *substance use* were 2 themes with more than a 75% increase in Google Trends searches (oral health weighted average Google Trends increase: 98.9%, 2010 less frequent Google Trends terms average use: 16.7, 2019 less frequent Google Trends terms average use: 32.78, 2010 more frequent Google Trends terms average use: 14.6, 2019 more frequent Google Trends terms average use: 29.6; substance use weighted average Google Trends increase: 77.7%, 2010 less frequent Google Trends terms average use: 1.9, 2019 less frequent Google Trends terms average use: 4.8, 2010 more frequent Google Trends terms average use: 9.4, 2019 more frequent Google Trends terms average use: 10.9) and more than a 250% increase in use in F990H text from 2010 to 2019 (oral health 261.9%, 2010: 429/2328, 18.43%; 2019: 1085/1627, 66.69%; substance use: 308% 2010: 403/2328, 17.31%; 2019: 1149/1627, 70.62%).

### Semantic Search

Key findings from the semantic search are shown in Table 2 for the most common phrases in themes with large percentage point increases in F990 Schedule H use from 2010 to 2019. These results highlight the presence of both action and nonaction statements in the free-response text submitted by hospitals.

**Table 2:** Results from semantic search, separated into results that imply action and results that imply no action.

Theme	Statements implying action	Statements implying no action
LBGTQ^a^	“A LGBT+ business group was formed and is working on addressing diversity and inclusion in health care.”	None documented in search results.
LBGTQ	“We earned top marks for our commitment to equitable inclusive care for LGBT patients and their families.”	None documented in search results.
SDOH^b^	“This program tracked social determinant of health data.”	None documented in search results.
SDOH	“The model addresses social determinants of health.”	None documented in search results.
SDOH	“Social determinants of health were deemed an underlying current of all priorities.”	None documented in search results.
Environment	“Helped educate participants about the dangers of excessive heat and injuries caused by extensive heat.”	“The hospital will not address ‘climate and health’ because the topic is not the hospital’s area of expertise.”
Environment	“For many years, we have been conducting lead risk assessments.”	“We have concluded that addressing these environmental issues falls outside of our mission.”
Environment	“The program addressed enforcement of pest control and regular air filter changes with the housing authority in appropriate housing units.”	“Improvement of air quality is not a part of the hospital’s mission.”
Substance use	“These programs target substance abuse prevention treatment and justice efforts.”	“Will not take action on mental health and substance abuse.”
Substance use	“Promote mental health and prevent substance abuse.”	“The only need not being address is substance abuse.”
Substance use	“Provide in-kind leadership and support to the implementation of the substance abuse action plan developed by the—behavioral health collaborative.”	“The organization has chosen not to address alcoholism and alcohol abuse in adults.”
Oral health	“Reduce the number of oral health related emergency department visits.”	“Also not being addressed at this time will be oral health.”
Oral health	“A consensus was reached that ongoing oral health efforts should be sustained.”	“Oral health: the dental provider of the institution left the organization in FY18.”
Oral health	“Providers promote the importance of oral health to patients and where they can seek services in the area.”	“Limited resources excluded this as an area chosen for action: oral health”
Nutrition	“In 2014 the group introduced and implemented interventions to promote nutrition and reduce childhood obesity.”	“The nutrition services provided are no longer covered by insurance or Medicare.”
Nutrition	“This intervention aimed to increase activity levels and nutrition education among students at title 1 schools.”	“Access to healthy food: this priority will not be addressed.”

^a^LGBTQ: lesbian, gay, bisexual, transgender, and queer.

^b^SDOH: social determinants of health.

## Discussion

### Principal Findings

Given the increased attention paid to health equity and disparities [27], it is gratifying to see greater use by hospital reporting entities of language related to these terms across all 29 health equity and disparity themes in this analysis. This increased use may suggest an improved hospital awareness of these issues or community needs related to these issues. It is worth noting that this increased use of words and phrases related to these specific themes is, with 2 exceptions, not required by the IRS and is therefore mostly voluntary on the part of hospitals.

The 2 exceptions to voluntary reporting are the *affordability* and *data collection* themes. Both topics require explicit descriptions in community benefit documentation. *Data collection* is of particular interest, because the 2010 Affordable Care Act required the implementation of CHNAs, including data collection and focus groups, by 2013. *Data collection* was one of the themes with the largest percentage point increase, with a particularly large jump (approximately 22 percentage points) between 2012 and 2013. This reflects the impact that data collection legislation can have on both the implementation and documentation of meeting requirements.

Some of the relative changes in theme use in the F990 documentation were parallel to national events. *Substance use* was a theme with a substantial percentage and percentage increase from 2010 to 2019 in F990H. This corresponds to the second wave of the US opioid epidemic, which began in 2010 with a rapid increase in heroin overdoses, and the third wave, which began in 2013 with significant overdoses resulting from synthetic opioids [28]. Heroin overdoses continued to increase in 2016, whereas synthetic opioid overdoses continued to increase in 2019. *Nutrition,* a theme that includes the term *obesity*, also increased from 2010 to 2019 in F990H. This use likely reflects an increase in obesity in the United States, and obesity-related conditions are among the leading causes of preventable premature death [29]. *SDOH* gained increased attention in 2010 when the World Health Organization published the “Conceptual Framework for Action on the Social Determinants of Health” [30]. The substantial increase in use during a similar timeframe suggests growing awareness among hospitals regarding the SDOH framework.

Variation in use can also be attributed to geography. Figure 3 shows that the clearest depiction of geographic differences as the percentage of reporting entities in states on the West Coast, using key terms related to homelessness, grew substantially between 2010 and 2019. This may reflect on the fact that WA, OR, and CA were among the top 10 states with the highest rates of homelessness in 2019 [31]. However, of these 3 states, only CA was also among the states with a high percentage increase in homelessness from 2010 to 2019. Other states with a large increase in documented homelessness from 2010 to 2019 included NY, SD, KS, and MA, but among these, only MA saw a corresponding increase in *homelessness* terms in F990H [31]. Chen et al [12] also documented lower mentions of investment in housing in F990H, with only 12 of 47 hospital organizations in 5 cities with high rates of homelessness reporting housing-related spending between 2015 and 2017. These findings suggest a lack of concordance in community needs and hospital community benefits spending in regions with high current or markedly increasing rates of homelessness. Chen et al [12] suggested that hospitals should be provided with evidence-based strategies from early adopters of homelessness strategies to see how housing may fit within their purview and that F990H instructions should be updated so that hospitals are getting adequate credit for housing investment.

The Moran results in Table 1 highlight other themes with discrepancies in F990H theme use between neighboring states and regions with a documented need. For example, *oral health* has clusters of F990H use in the Mountain West, mid-Atlantic, and New England, but all the states in the clusters with higher F990H *oral health* use are also among states with medium to high proportions of adults reporting in 2018 that they had visited a dentist or dental clinic within the past year [32]. As regular dental visits are important to both oral health and overall wellness, it is notable that no state among those with the lowest proportion of previous year adult dental visits was among the states with the highest mentions of oral health in F990H [33]. The F990H use of the *immigration* theme was clustered in the Pacific Northwest and mid-Atlantic and distinctly lower in the 3 states where nearly half (45%) of US immigrants live: California, Texas, and Florida [34]. This insight aligns with the documented finding that adult immigrants, regardless of immigration and citizenship status, are underserved in the US health care system [35]. Not all F990H use was misaligned—the use of *rural* was high in states from more rural regions (Midwest and Mountain West) and showed some overlap with the *health IT* theme, potentially reflecting the pre–COVID-19 pandemic emphasis on the use of health IT approaches such as telehealth in rural communities [36,37]. The *substance use* theme was highest in the Southwest, Midwest, and East Coast, all regions where most states maintained similar opioid overdose rates between 2017 and 2018 [38]. However, the remaining themes with statistically significant geographic clustering—*equity,* *LGBTQ,* and *SDOH*—are all themes with national applicability that transcend the few clusters with higher F990H use. The clustered use in the West Coast and North Atlantic, with some sporadic clustered use in the Midwest, suggests that these terms may be politically charged and more commonly used in politically liberal states [39].

Themes including *LGBTQ*, *disability, oral health,* and *race and ethnicity* are more highly ranked in Google Trends than in F990H, suggesting a degree of misalignment in general public interests and hospital activities as described in F990H. Individuals may be more interested in how these personal topics impact them, whereas hospital reporting entities discuss *insurance* and *activity* themes much more in F990H than in the broader population. This is likely expected with the current version of F990H as it includes specific questions related to how hospitals address uninsured patients but has ambiguous questions requesting information on how a hospital is addressing “significant needs” as identified in a CHNA or why a significant need is not being addressed. Given that a major motivation of community benefits reporting is to ensure that nonprofit hospitals are addressing community needs, the misalignment in ranking of some themes in Google Trends health searches and F990H free-response text suggests that a revamp of F990H with more explicit and granular community needs questions may generate greater accountability for addressing health equity and disparities.

A notable limitation of the Google Trends comparison is that the trends are from national searches, whereas hospital reporting entities generally aim to align with local community needs. Although national searches may not always be applicable to a local community, attention to larger trends may still help hospital entities that are missing opportunities to address important rising topics that are still relevant to their community. For example, the increased attention in the past decade to LGBTQ rights and disparities by race and ethnicity suggests that both *LGBTQ* and *race and ethnicity* have national applicability, and the Google Trends results suggest that they are currently more prioritized by the public than by hospitals. More hospitals could seek to discuss how they are addressing these themes in the F990H.

The increased use of terms related to health equity and disparities is promising, but the semantic search results in Table 2 make it clear that increased use of terms may not necessarily correspond to increased community benefit programming. Of the 6 themes with the largest relative or raw increase, 4 themes had results with hospitals explicitly stating their inaction. A common reason for lack of action often appears to be a lack of resources or services or misalignment with the hospital’s mission. Young et al [7] found that most benefit-related expenditures were related to patient care, rather than community health improvement. Sapirstein et al [9] found no evidence of dramatic shifts in community benefit spending from 2014 to 2019. Hospitals may be mentioning community-related themes more but may not actually take additional action or allocate funding to new community benefits themes. Governing authorities such as the IRS could better scrutinize these hospital statements, such as the inconsistency in Table 2, where 1 hospital says that improvement of air quality does not align with a hospital’s mission, whereas a different hospital says that it provides air filters to a housing authority. Similar to the recommendation of Chen et al [12] for improved reporting of efforts to address homelessness in F990H, the IRS should clarify that providing air filters to meet a community need is mission-aligned and credit hospitals reporting efforts such as this on F990H [12].

Although it is exciting to see that hospitals use more language related to health equity and disparities, it cannot be presumed that there is a corresponding increase in activities related to these needs; mentions in text alone cannot prove action. Policy makers should consider additional language in F990H that requires a clear description of health equity and disparities, including explicit recognition of work on SDOH as a community benefit [10,11]. The Government Accountability Office has called for updating Form 990, including Schedule H, to more clearly, consistently, and comprehensively describe community benefit activities, as well as for Congress to specify which services and activities are sufficient to meet community benefit standards [5]. These updates could be used to improve auditing of community benefits, contribute to efforts to score hospitals on community benefits programming, or highlight innovative hospitals providing exemplary community benefits. Greater transparency, documentation of activities, and community benefits–specific IRS audit processes for F990H could lead to increased accountability and action by hospitals to address community health equity and disparities. In their study, Chen et al [12] also recommended that the IRS seek to ensure greater alignment of F990H activities with CHNAs [12]. The study by Carlton and Singh [40] found that joint CHNAs with hospital-local health department collaboration encouraged greater hospital investment in community health improvement activities. Further research can use text analytics to explore the programs that hospitals describe in F990H and assess their alignment with implementation plans in CHNAs.

### Conclusions

We created a health equity and disparities term list and showed increased use of terms across all 29 themes by hospital reporting entities in free-response text submitted annually from 2010 to 2019 in F990H. We found variations across years and geographies. We suggest that hospitals demonstrate an increased awareness of health equity and disparities yet also show potential misalignment with public interests, as demonstrated through Google Trends and varying changes in action or programming with semantic search. Further research can continue to explore the degree to which hospitals have satisfactorily addressed community needs, as described in the free-response text. Policy changes to the F990H could improve transparency and accountability related to hospital community benefit efforts to address health equity and disparities.

## Appendix

Multimedia Appendix 1 Details the requirements of Form 990 Schedule H Part V Section C and Schedule H Part VI.

Multimedia Appendix 2 Additional details on the Google Trends methodology.

Multimedia Appendix 3 Details of the percentage of hospital reporting entities with one or more uses of a word or phrase in each theme in a given tax year. The figures are sorted according to the percentage of hospitals using one or more terms in 2019.

Multimedia Appendix 4 The figures provide additional details of the geographic variation for each theme by illustrating the percentage of hospital reporting entities by state with one or more uses of a particular theme.

## Abbreviations

CHNA: community health needs assessment
IRS: Internal Revenue Service
LGBTQ: lesbian, gay, bisexual, transgender, and queer
SDOH: social determinants of health
