# Supplementary material for: Text Analysis of Trends in Health Equity and Disparities From the Internal Revenue Service Tax Documentation Submitted by US Nonprofit Hospitals Between 2010 and 2019: Exploratory Study
Source: J Med Internet Res. 2023 May 24;25:e44330. doi: 10.2196/44330 (PMC10248774; doi:10.2196/44330)
Supplement: Multimedia Appendix 1 [file jmir_v25i1e44330_app1.docx]

**Details on Form 990 Schedule H Text Requirements**

The free response text was obtained from Form 990 Schedule H Part V Section C and Schedule H Part VI. The following Table 1 summarizes the information described in the IRS Instructions for Schedule H.

Table 1: Guidance from IRS Instructions for Schedule H for Part V Section C and Part IV

| Part | Description from IRS Instructions | Relevant Schedule H Lines |
| --- | --- | --- |
| Form 990 Schedule H Part V Section C: Facility Information | “In Part V, the organization must list all of its hospital facilities in Section A, complete separate Sections B and C for each of its hospital facilities or facility reporting groups listed in Section A, and list its non-hospital health care facilities in Section D.”  “Section C. Supplemental Information for Part V, Section B. Provide descriptions required for Part V, Section B, lines 2, 3j, 5, 6a, 6b, 7d, 11, 13b, 13h, 15e, 16j, 18e, 19e, 20a, 20b, 20c, 20d, 20e, 21c, 21d, 23, and 24.” | Line 2: Was the hospital facility acquired or placed into service as a tax-exempt hospital in the current tax year or the immediately preceding tax year? If “Yes,” provide details of the acquisition in Section C  Line 3j: During the tax year or either of the two immediately preceding tax years, did the hospital facility conduct a community health needs assessment (CHNA)? If “Yes,” indicate what the CHNA report describes; Other (describe in Section C)  Line 5: In conducting its most recent CHNA, did the hospital facility take into account input from persons who represent the broad interests of the community served by the hospital facility, including those with special knowledge of or expertise in public health? If “Yes,” describe in Section C how the hospital facility took into account input from persons who represent the community, and identify the persons the hospital facility consulted  Line 6a: Was the hospital facility’s CHNA conducted with one or more other hospital facilities? If “Yes,” list the other hospital facilities in Section C  Line 6b: Was the hospital facility’s CHNA conducted with one or more organizations other than hospital facilities? If “Yes,” list the other organizations in Section C  Line 7d: Did the hospital facility make its CHNA report widely available to the public? If “Yes,” indicate how the CHNA report was made widely available; Other (describe in Section C)  Line 11: Describe in Section C how the hospital facility is addressing the significant needs identified in its most recently conducted CHNA and any such needs that are not being addressed together with the reasons why such needs are not being addressed.  Line 13b: Explained eligibility criteria for financial assistance, and whether such assistance included free or discounted care? If “Yes,” indicate the eligibility criteria explained in the FAP; Income level other than FPG (describe in Section C)  Line 13h: Explained eligibility criteria for financial assistance, and whether such assistance included free or discounted care? If “Yes,” indicate the eligibility criteria explained in the FAP; Other (describe in Section C)  Line 15e: Explained the method for applying for financial assistance? If “Yes,” indicate how the hospital facility’s FAP or FAP application form (including accompanying instructions) explained the method for applying for financial assistance; Other (describe in Section C)  16j: Was widely publicized within the community served by the hospital facility? If “Yes,” indicate how the hospital facility publicized the policy; Other (describe in Section C)  18e: Check all of the following actions against an individual that were permitted under the hospital facility’s policies during the tax year before making reasonable efforts to determine the individual’s eligibility under the facility’s FAP; Other similar actions (describe in Section C)  19e: Did the hospital facility or other authorized party perform any of the following actions during the tax year before making reasonable efforts to determine the individual’s eligibility under the facility’s FAP? Other similar actions (describe in Section C)  20: Indicate which efforts the hospital facility or other authorized party made before initiating any of the actions listed (whether or not checked) in line 19: Provided a written notice about upcoming ECAs (Extraordinary Collection Action) and a plain language summary of the FAP at least 30 days before initiating those ECAs (if not, describe in Section C); Made a reasonable effort to orally notify individuals about the FAP and FAP application process (if not, describe in Section C); Processed incomplete and complete FAP applications (if not, describe in Section C); Made presumptive eligibility determinations (if not, describe in Section C); Other (describe in Section C)  21: Did the hospital facility have in place during the tax year a written policy relating to emergency medical care that required the hospital facility to provide, without discrimination, care for emergency medical conditions to individuals regardless of their eligibility under the hospital facility’s financial assistance policy? The hospital facility limited who was eligible to receive care for emergency medical conditions (describe in Section C); Other (describe in Section C)  23: During the tax year, did the hospital facility charge any FAP-eligible individual to whom the hospital facility provided emergency or other medically necessary services more than the amounts generally billed to individuals who had insurance covering such care? If “Yes,” explain in Section C.  24: During the tax year, did the hospital facility charge any FAP-eligible individual an amount equal to the gross charge for any service provided to that individual? If “Yes,” explain in Section C. |
| Form 990 Schedule H Part VI: Supplemental Information | “Use Part VI to provide the narrative explanations required by the following questions, and to supplement responses to other questions on Schedule H (Form 990). In addition, use Part VI to make disclosures described in section 7 of Rev. Proc. 2015-21. Identify the specific part, section, and line number that the response supports, in the order in which they appear on Schedule H (Form 990). Part VI can be duplicated if more space is needed.” | 1 Required descriptions: Provide the descriptions required for Part I, lines 3c, 6a, and 7; Part II and Part III, lines 2, 3, 4, 8, and 9b.   - Part I Line 3c: If applicable, describe the criteria used for determining eligibility for free or discounted care under the organization's FAP. Also, describe whether the organization uses an asset test or other threshold, regardless of income, to determine eligibility for free or discounted care. - Part I Line 6a: If the organization's community benefit report is in a report prepared by a related organization, and not in a separate report prepared by the organization, identify the related organization and list its EIN. - Part I Line 7: If applicable, describe if the organization included as subsidized health services any costs attributable to a physician clinic, and enter such costs the organization included. - Part II: Describe how the organization’s community building - activities, as reported in Part II, promote the health of the - community or communities the organization serves. - Part III Line 2: Describe the methodology used to determine the amount on Part III, line 2, including how the organization accounts for discounts and payments on patient accounts in determining bad debt expense. - Part III Line 3: Describe the methodology used to determine the amount entered on line 3. Also, describe the rationale, if any, for including any portion of bad debt as community benefit. - Part III Line 4: Provide, if applicable, the text of the footnote to the organization's financial statements that describes bad debt expense, or enter the page number(s) of the organization's most recent audited financial statements on which the footnote appears. - Part III Line 8: Describe the costing methodology used to determine the Medicare allowable costs entered on Part III, line 6. Describe, if applicable, the extent to which any shortfall entered on Part III, line 7, should be treated as a community benefit, and the rationale for the organization's position. - Part III Line 9b: If the organization has a written debt collection policy and answered “Yes,” to Part III, line 9b, describe the collection practices in the policy that apply to patients who it knows qualify for financial assistance, whether the practices apply specifically to such patients or also cover other types of patients.   2 Needs assessment. Describe how the organization assesses the health care needs of the communities it serves, in addition to any CHNAs reported in Part V, Section B.  3 Patient education of eligibility for assistance. Describe how the organization informs and educates patients and persons who may be billed for patient care about their eligibility for assistance under federal, state, or local government programs or under the organization’s financial assistance policy.  4 Community information. Describe the community the organization serves, taking into account the geographic area and demographic constituents it serves.  5 Promotion of community health. Provide any other information important to describing how the organization’s hospital facilities or other health care facilities further its exempt purpose by promoting the health of the community (for example, open medical staff, community board, use of surplus funds, etc.).  6 Affiliated health care system. If the organization is part of an affiliated health care system, describe the respective roles of the organization and its affiliates in promoting the health of the communities served.  7 State filing of community benefit report. If applicable, identify all states with which the organization, or a related organization, files a community benefit report |
