# Supplementary material for: Text Analysis of Trends in Health Equity and Disparities From the Internal Revenue Service Tax Documentation Submitted by US Nonprofit Hospitals Between 2010 and 2019: Exploratory Study
Source: J Med Internet Res. 2023 May 24;25:e44330. doi: 10.2196/44330 (PMC10248774; doi:10.2196/44330)
Supplement: Multimedia Appendix 2 [file jmir_v25i1e44330_app2.docx]

**Google Trends Methodology**

Google Trends returns the relative use of a term on a scale of 0 to 100 and allows for comparison of up to five terms. Since we have more than five terms, we used the most common Google Trends term (*cancer*) as the benchmark term for comparison. We noticed that several terms occurred less frequently with entirely zero usage in the time period relative to the initial benchmark term, so we used a second benchmark term (*teeth*) that was the most frequent Google Trend among those with <1 usage relative to the primary term as a comparison for the less frequent entries.

Google Trends returns relative usage by month. We averaged across months for each word or phrase, and then averaged across words or phrases in each year for the 29 themes. Since we used two benchmark terms, we separately averaged themes. We calculated the rank order of usage and relative change in usage by theme for more and less frequent usage. We then took a weighted average to determine the final rank usage of the theme in 2019 and the weighted relative change in usage from 2010 to 2019.
