# Supplementary material for: Text Analysis of Trends in Health Equity and Disparities From the Internal Revenue Service Tax Documentation Submitted by US Nonprofit Hospitals Between 2010 and 2019: Exploratory Study
Source: J Med Internet Res. 2023 May 24;25:e44330. doi: 10.2196/44330 (PMC10248774; doi:10.2196/44330)
Supplement: Multimedia Appendix 3 [file jmir_v25i1e44330_app3.docx]

**Detailed Figure Results by Key Term for Each Theme**

The following figures provide the percentage of hospital reporting entities with one or more uses of a word or phrase in each theme in a given tax year. Figures are sorted by percent of hospitals using one or more terms in 2019. Darker coloration is used as a visual aid to identify larger percentages as denoted by the legend below.


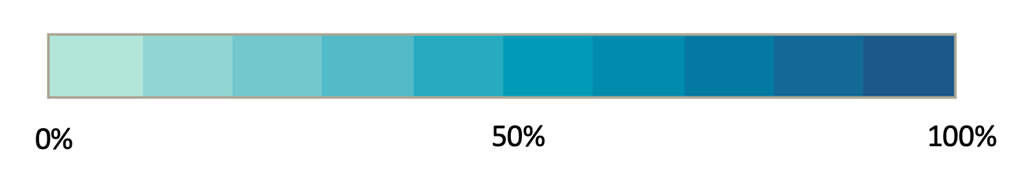


**Figure 1. Affordability Theme**


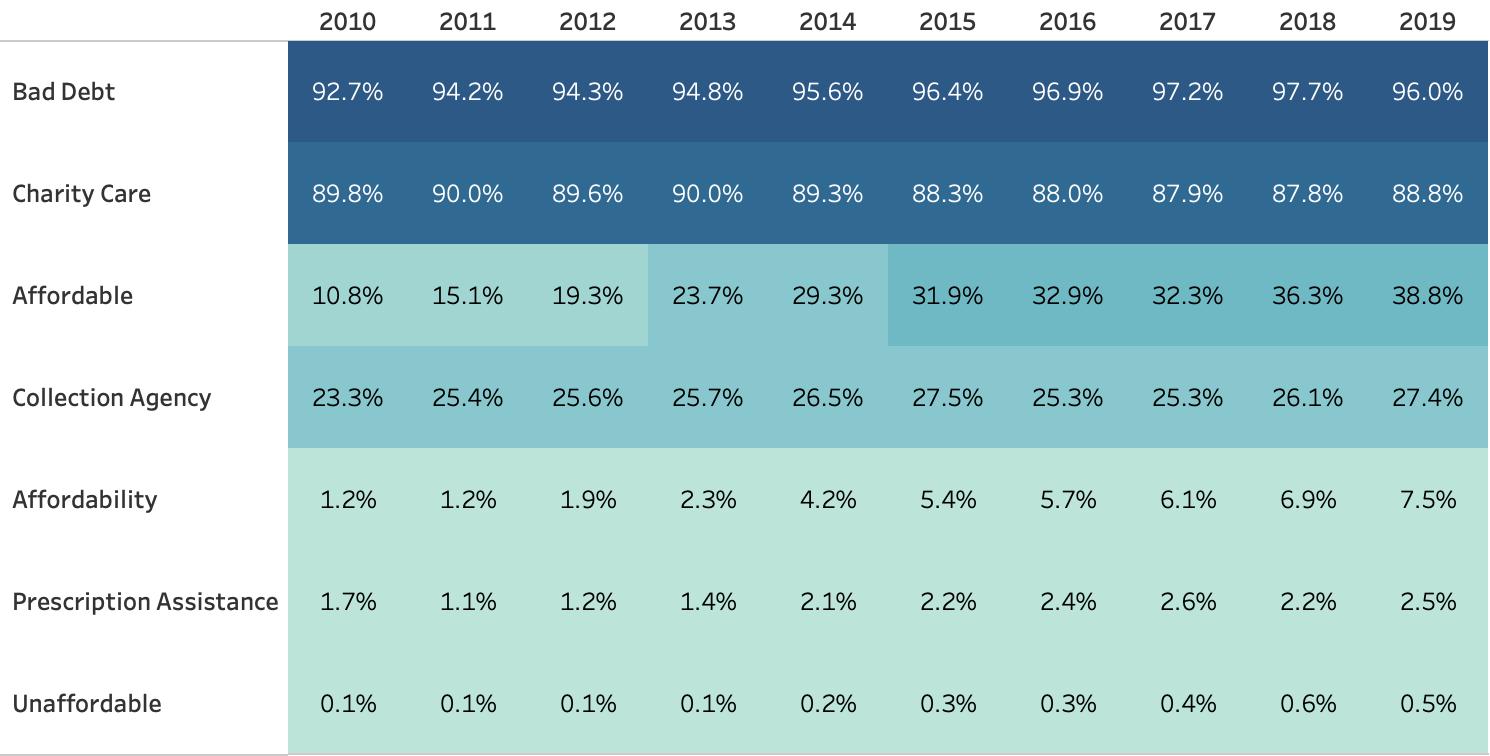


Percentage of hospital reporting entities with one or more uses of phrases in the *affordability* theme by year.

**sFigure 2. Government Organizations Theme**


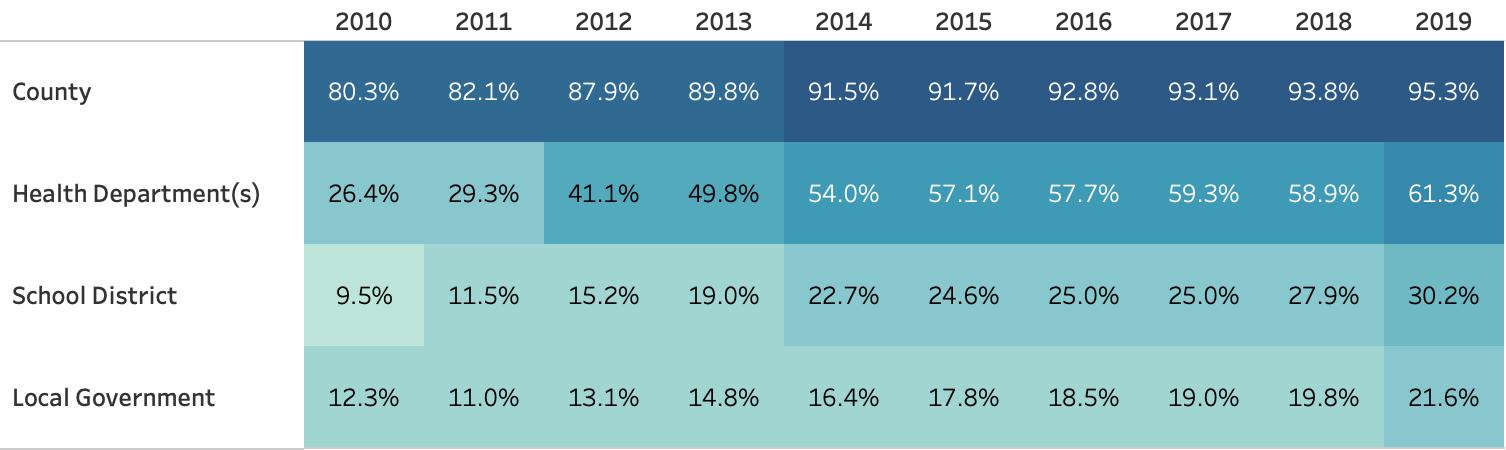


Percentage of hospital reporting entities with one or more uses of phrases in the *government organizations* theme by year.

**sFigure 3. Data Collection Theme**


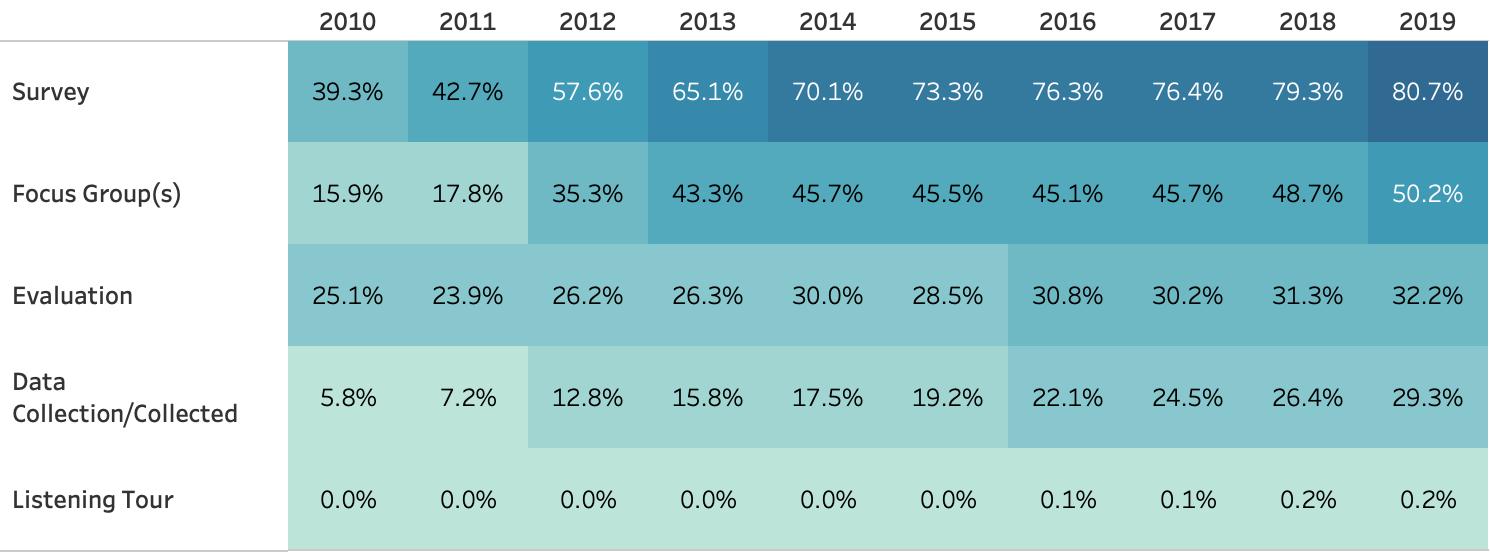


Percentage of hospital reporting entities with one or more uses of phrases in the *data collection* theme by year.

**sFigure 4. Mental Health Theme**


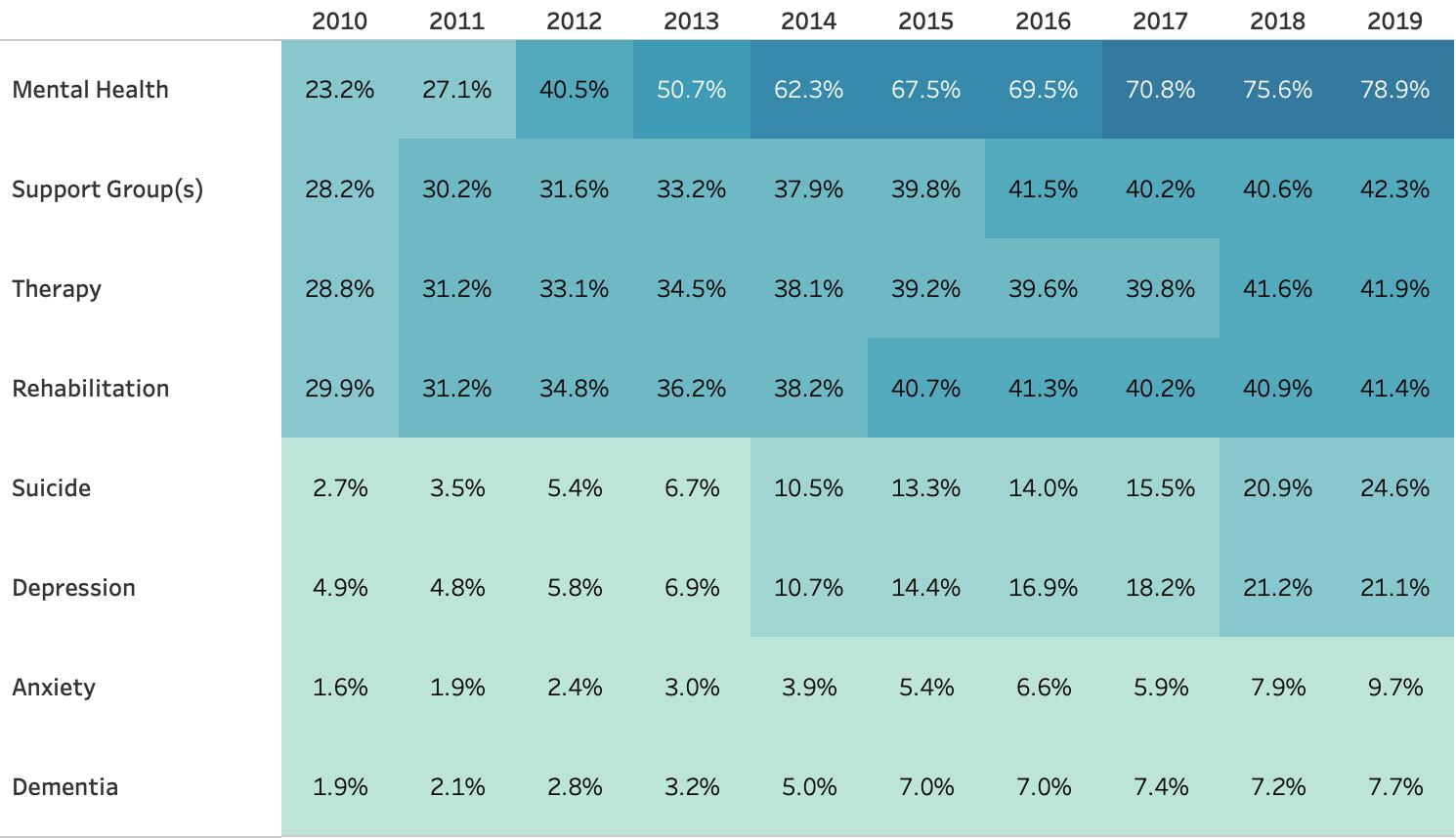


Percentage of hospital reporting entities with one or more uses of phrases in the *mental health* theme by year.

**sFigure 5. Poverty Theme**


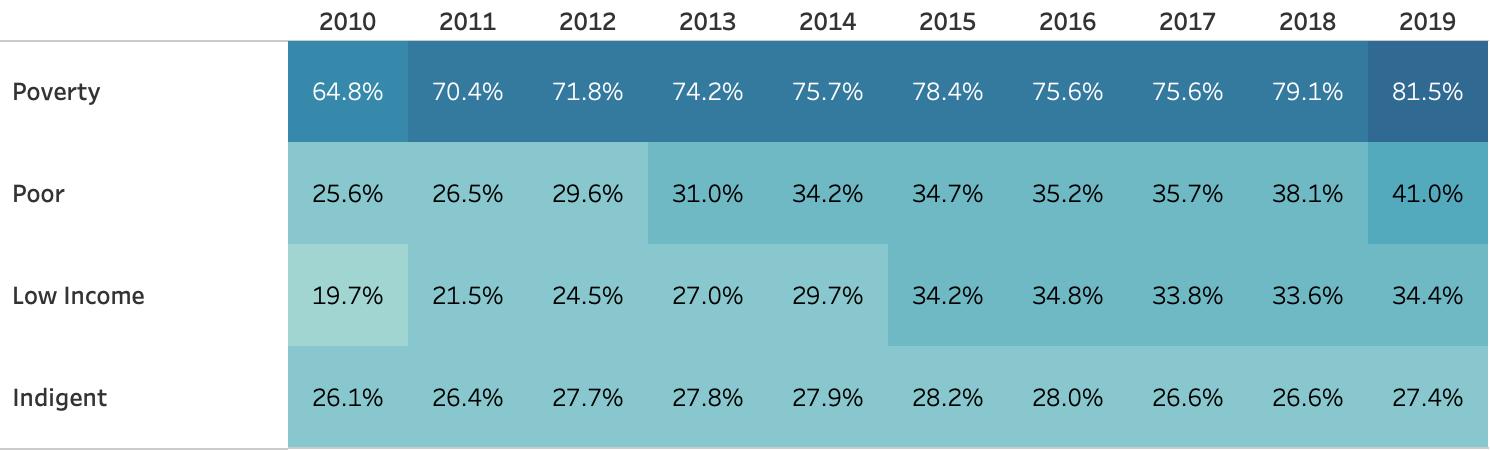


Percentage of hospital reporting entities with one or more uses of phrases in the *poverty* theme by year.

**sFigure 6. Insurance Theme**


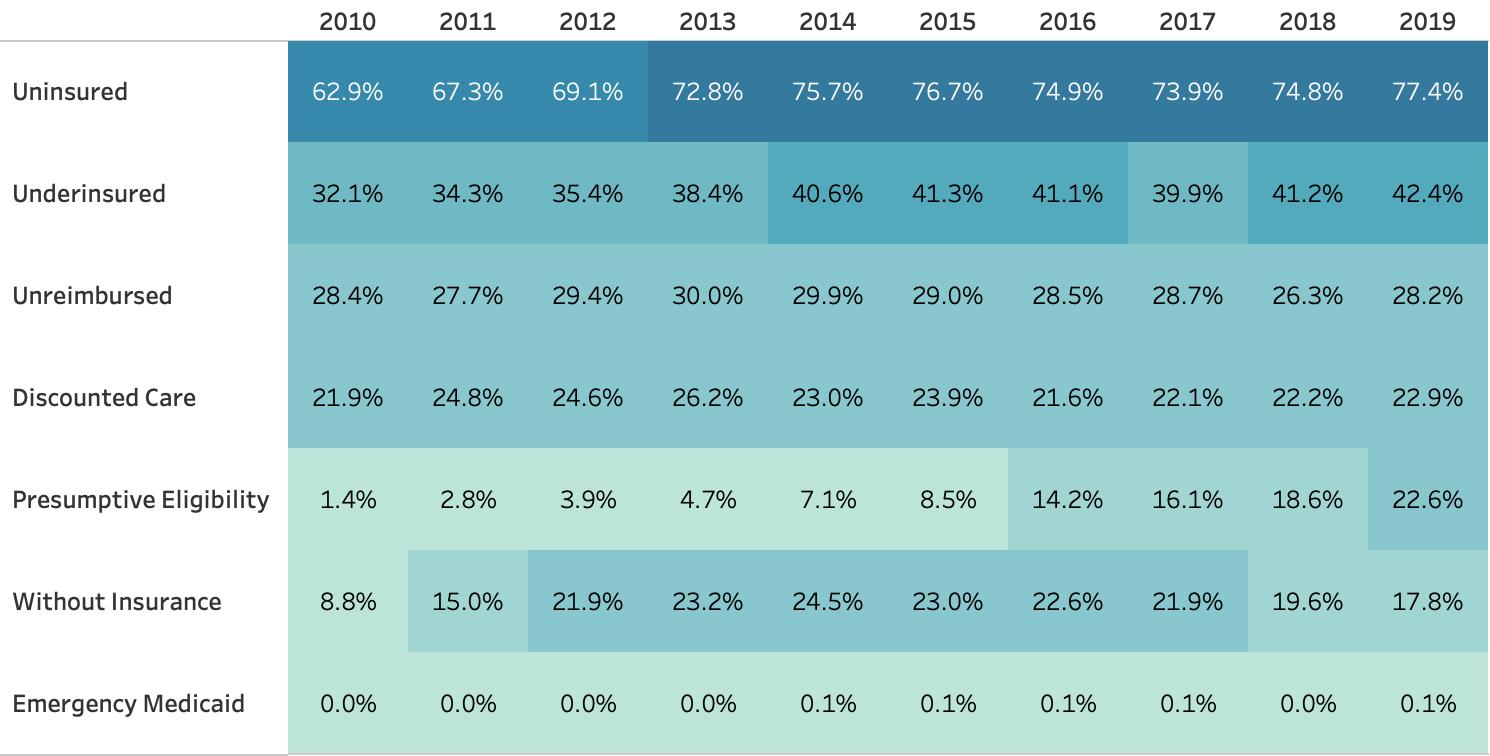


Percentage of hospital reporting entities with one or more uses of phrases in the *insurance* theme by year.

**sFigure 7. Activity Theme**


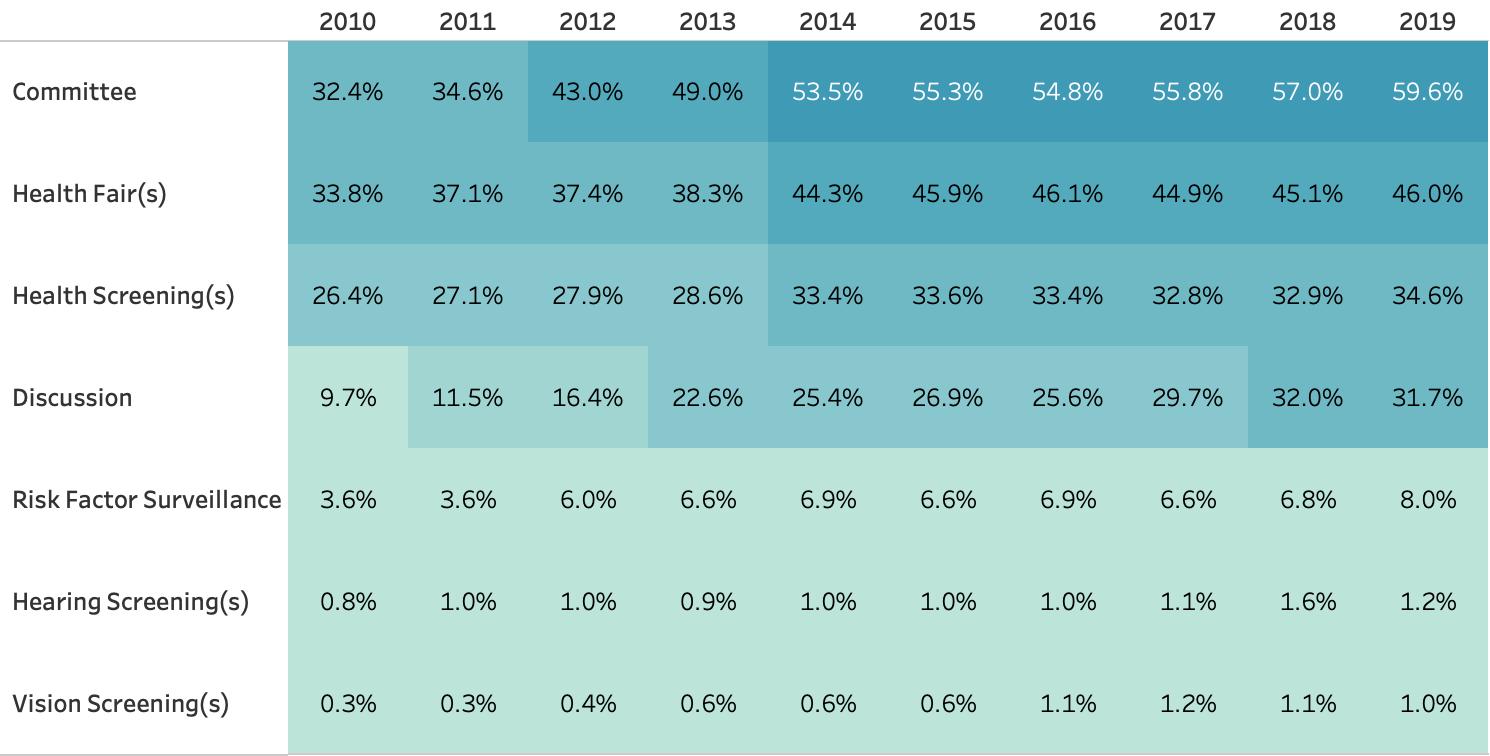


Percentage of hospital reporting entities with one or more uses of phrases in the *activity* theme by year.

**sFigure 8. Chronic Illness Theme**


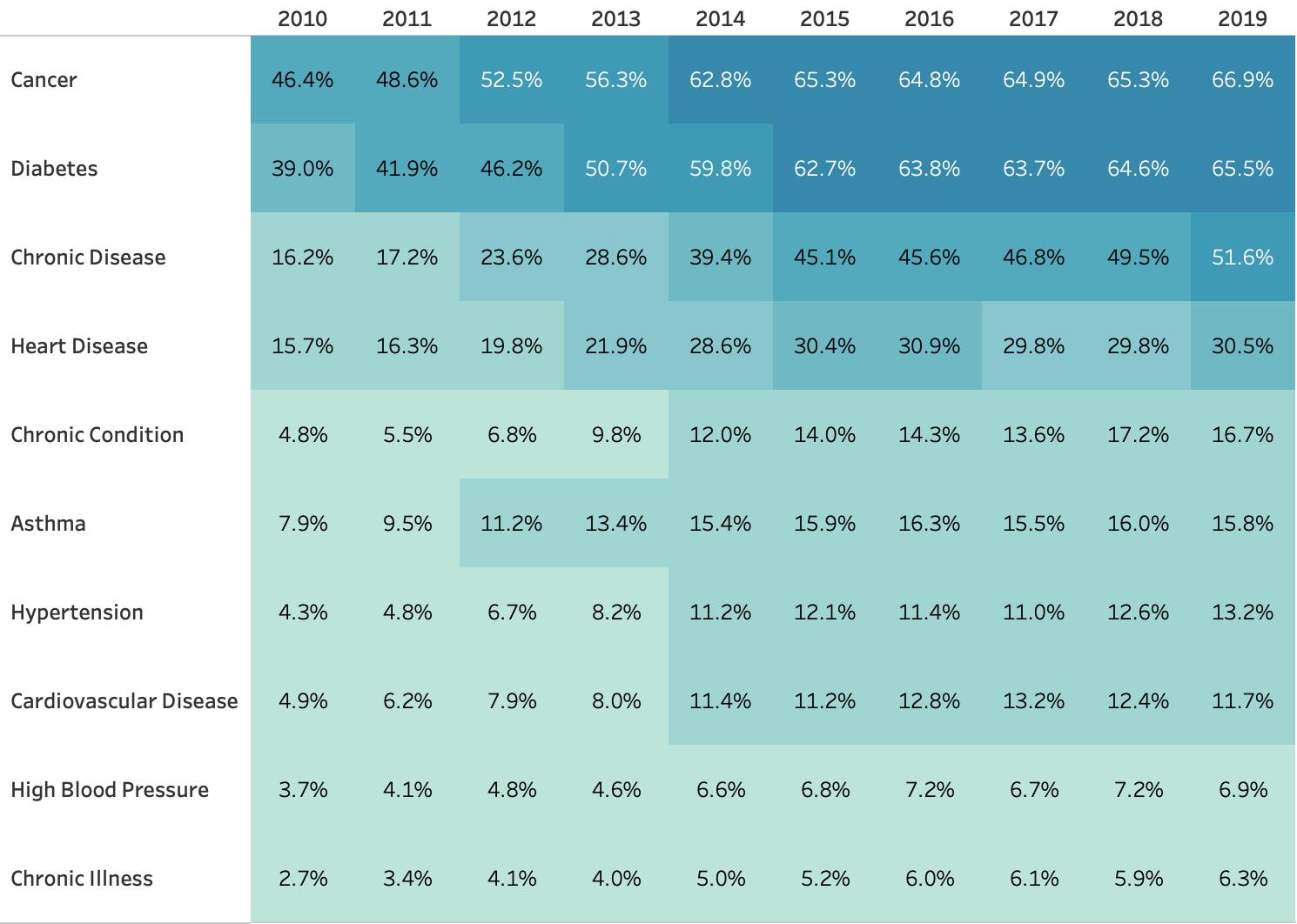


Percentage of hospital reporting entities with one or more uses of phrases in the *chronic illness* theme by year.

**sFigure 9. Child Theme**


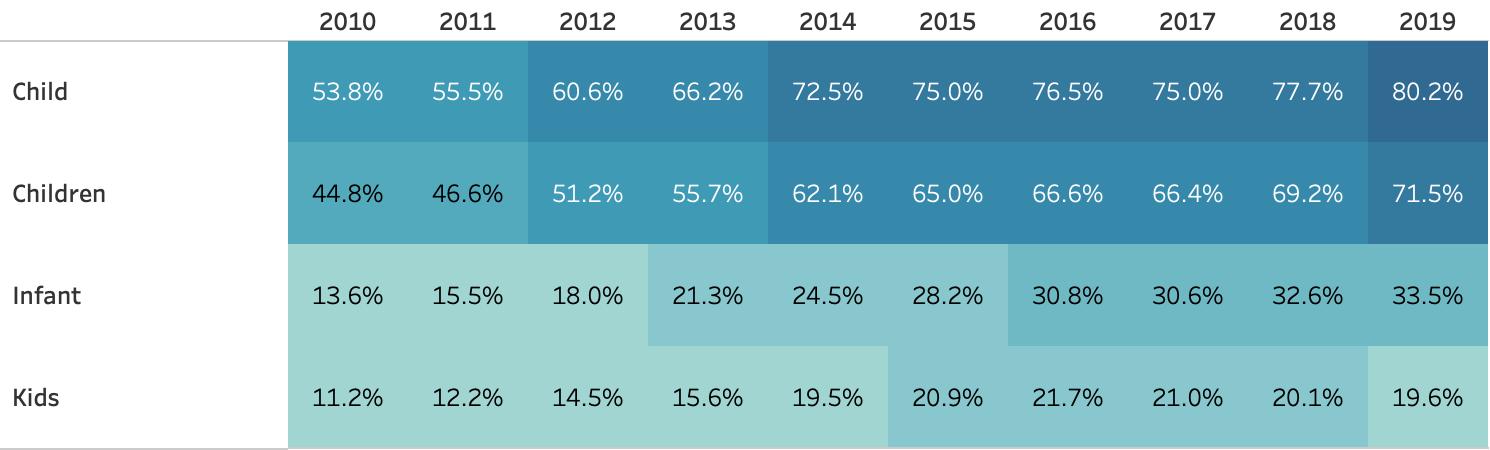


Percentage of hospital reporting entities with one or more uses of phrases in the *child* theme by year.

**sFigure 10. Senior Theme**


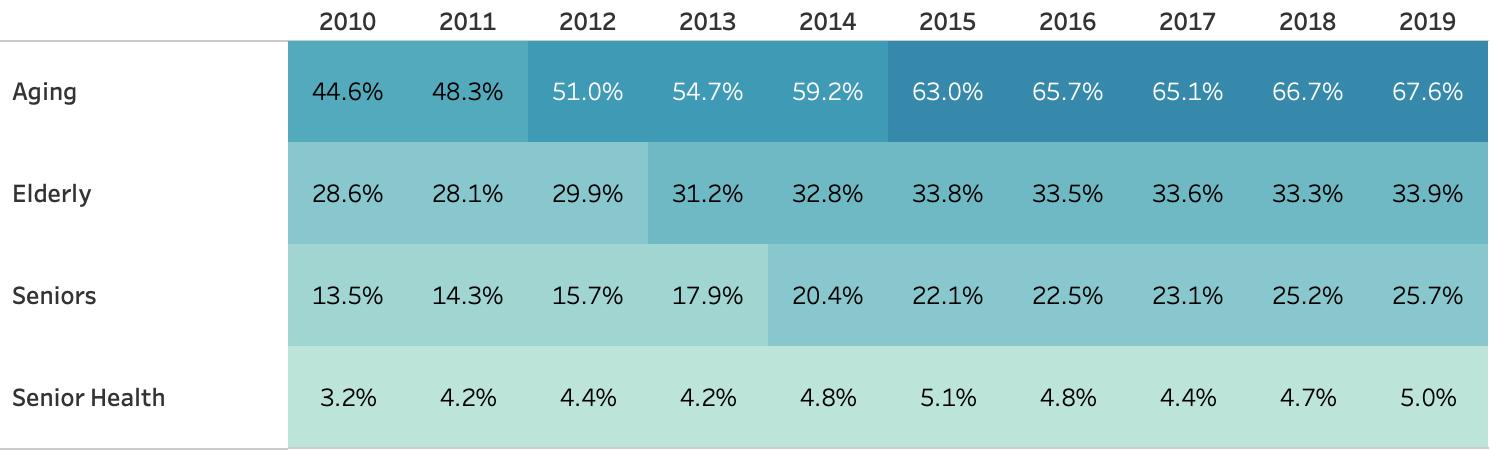


Percentage of hospital reporting entities with one or more uses of phrases in the *senior* theme by year.

**sFigure 11. Nutrition Theme**


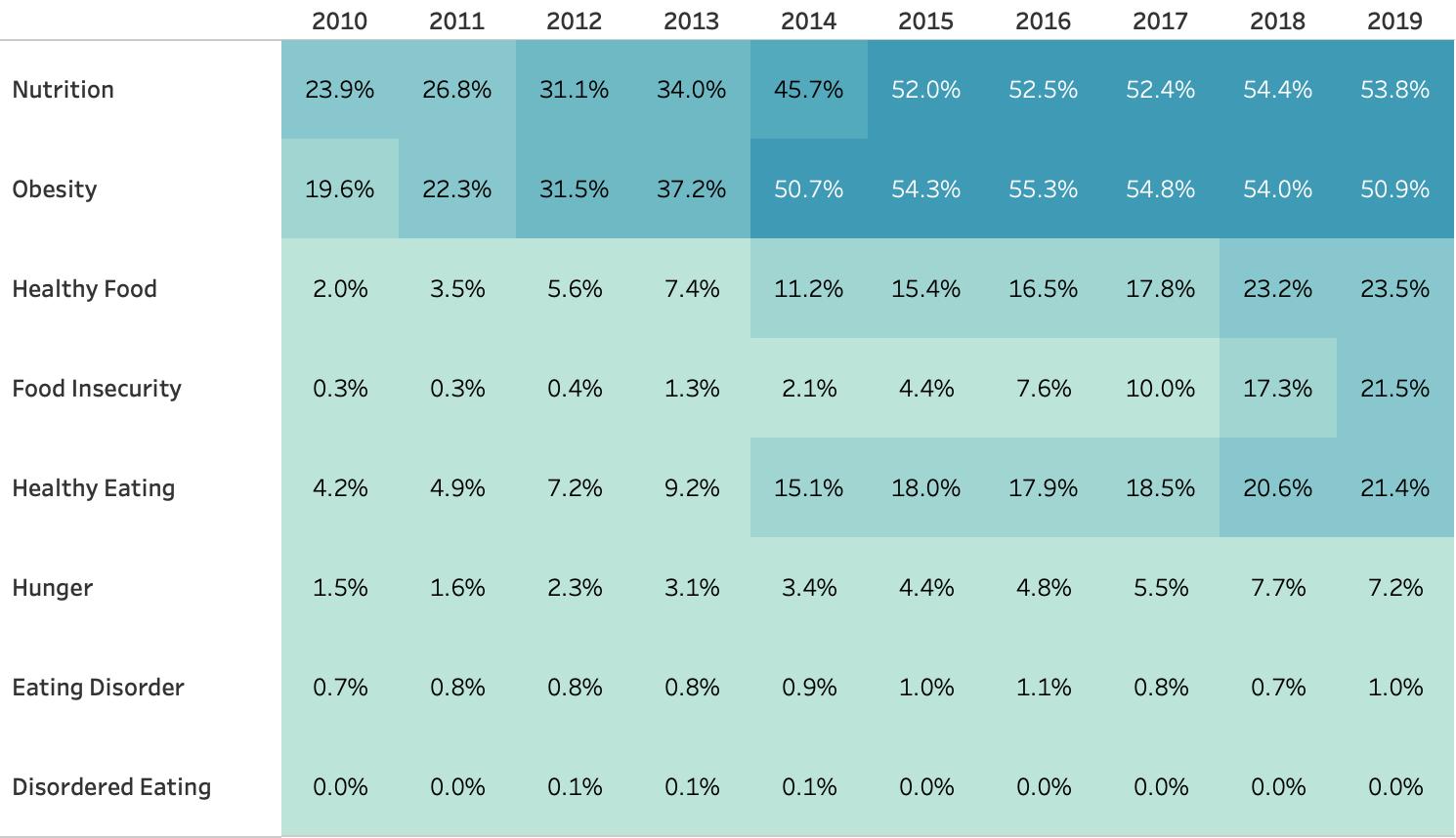


Percentage of hospital reporting entities with one or more uses of phrases in the *nutrition* theme by year.

**sFigure 12. Substance Use Theme**


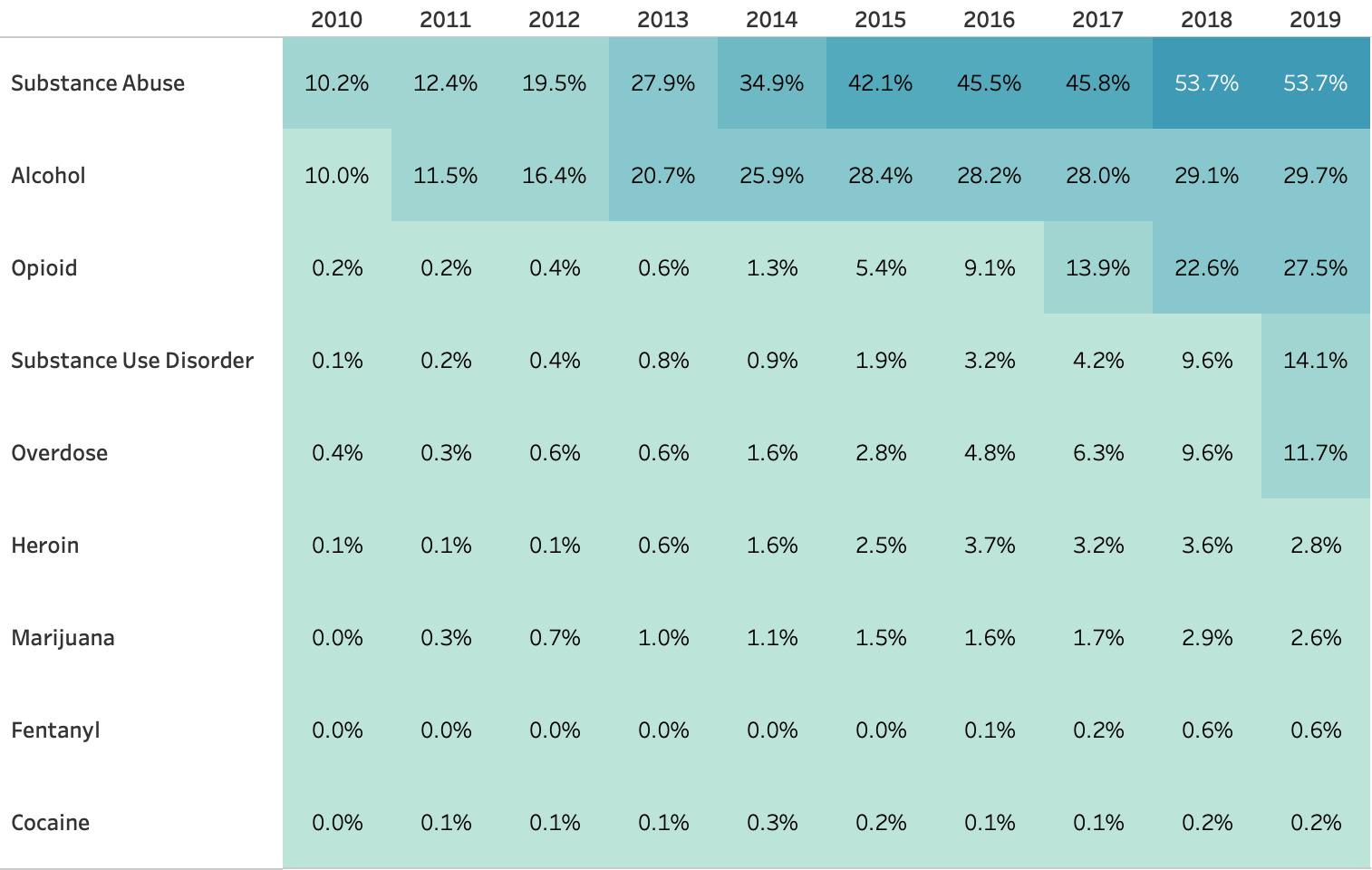


Percentage of hospital reporting entities with one or more uses of phrases in the *substance use* theme by year.

**sFigure 13. Oral Health Theme**


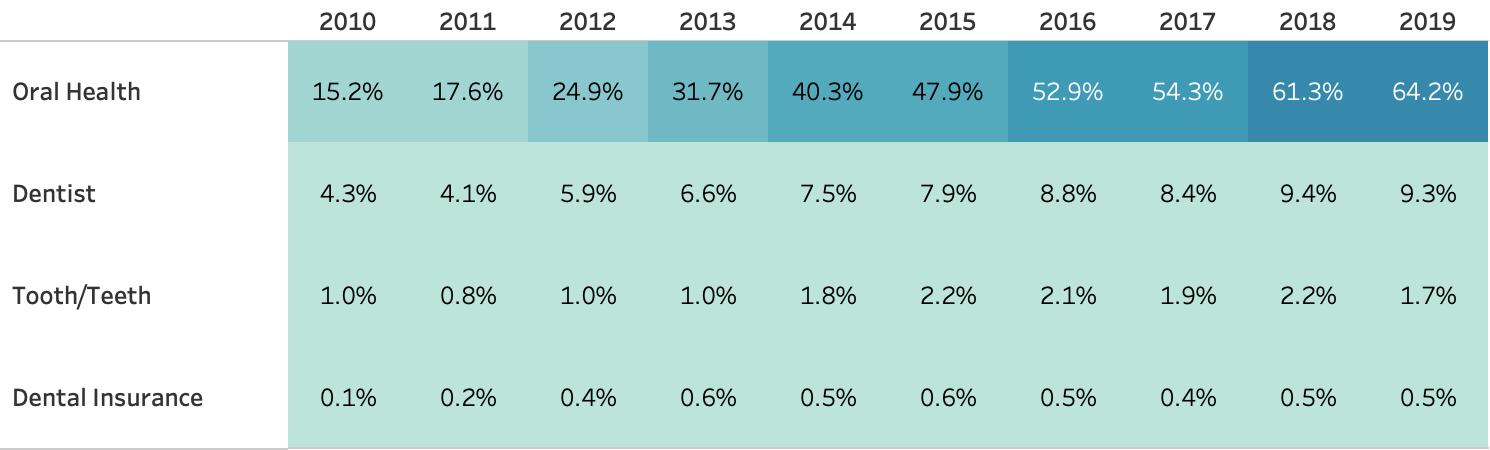


Percentage of hospital reporting entities with one or more uses of phrases in the *oral health* theme by year.

**sFigure 14. Race and Ethnicity Theme**


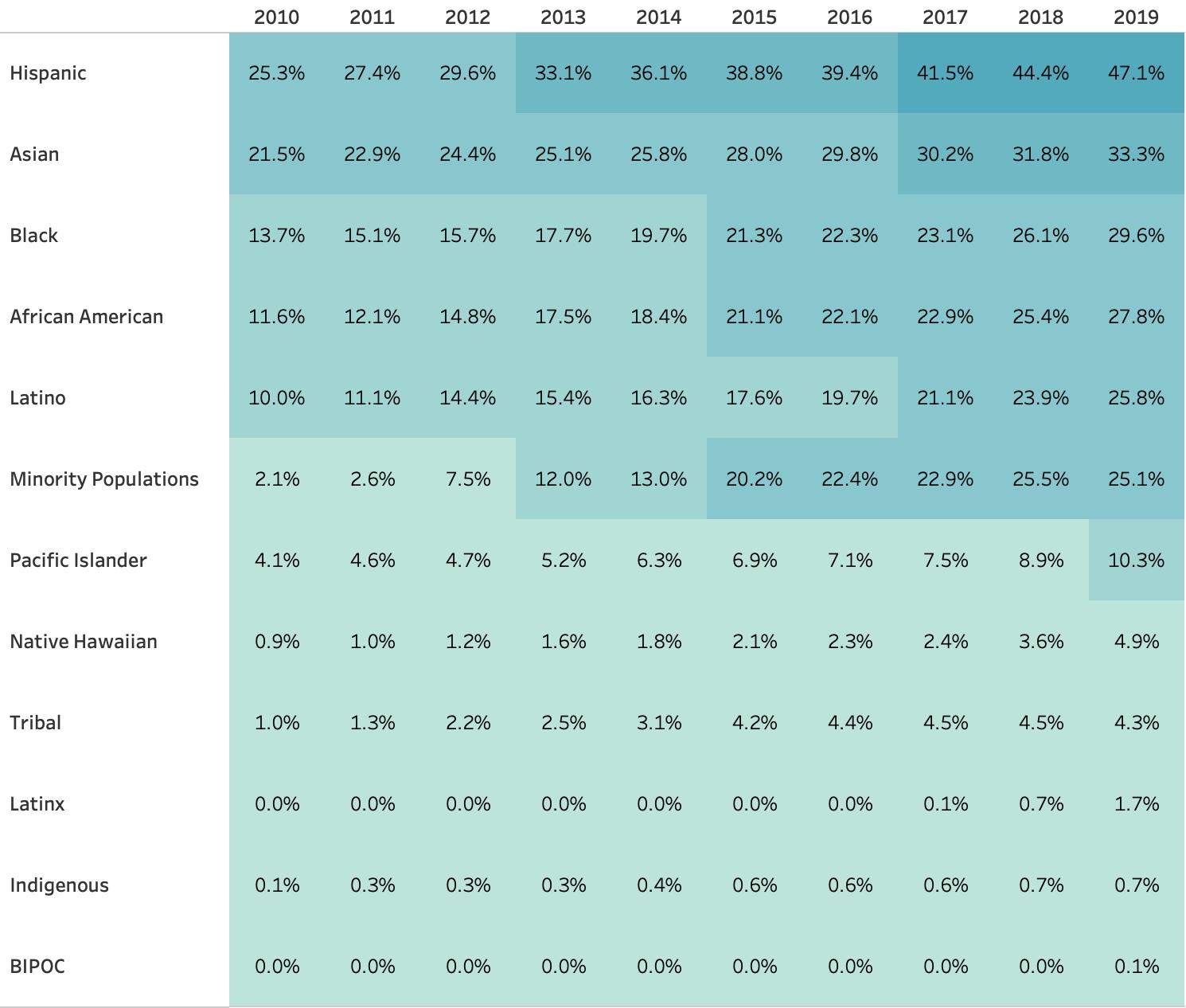


Percentage of hospital reporting entities with one or more uses of phrases in the *race and ethnicity* theme by year.

**sFigure 15. Exercise Theme**


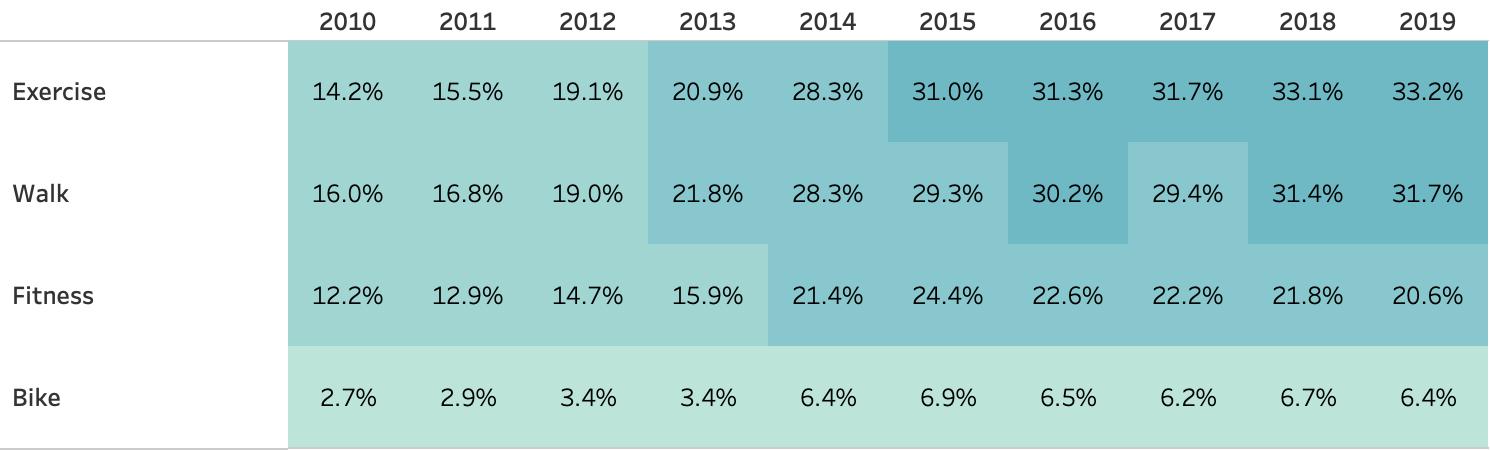


Percentage of hospital reporting entities with one or more uses of phrases in the *exercise* theme by year.

Rural (#16) is skipped as it has only one term.

**sFigure 17. Equity Theme**


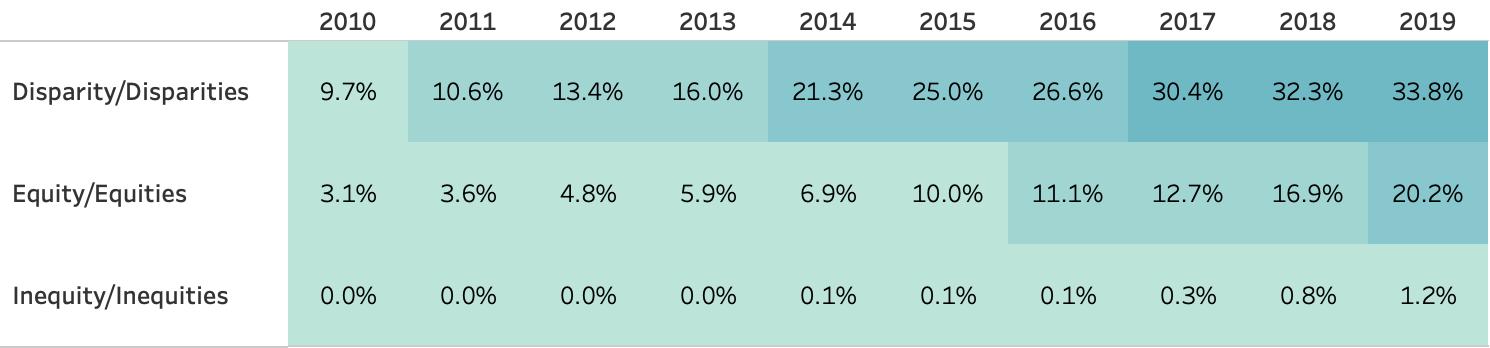


Percentage of hospital reporting entities with one or more uses of phrases in the *equity* theme by year.

**sFigure 18. Homelessness Theme**


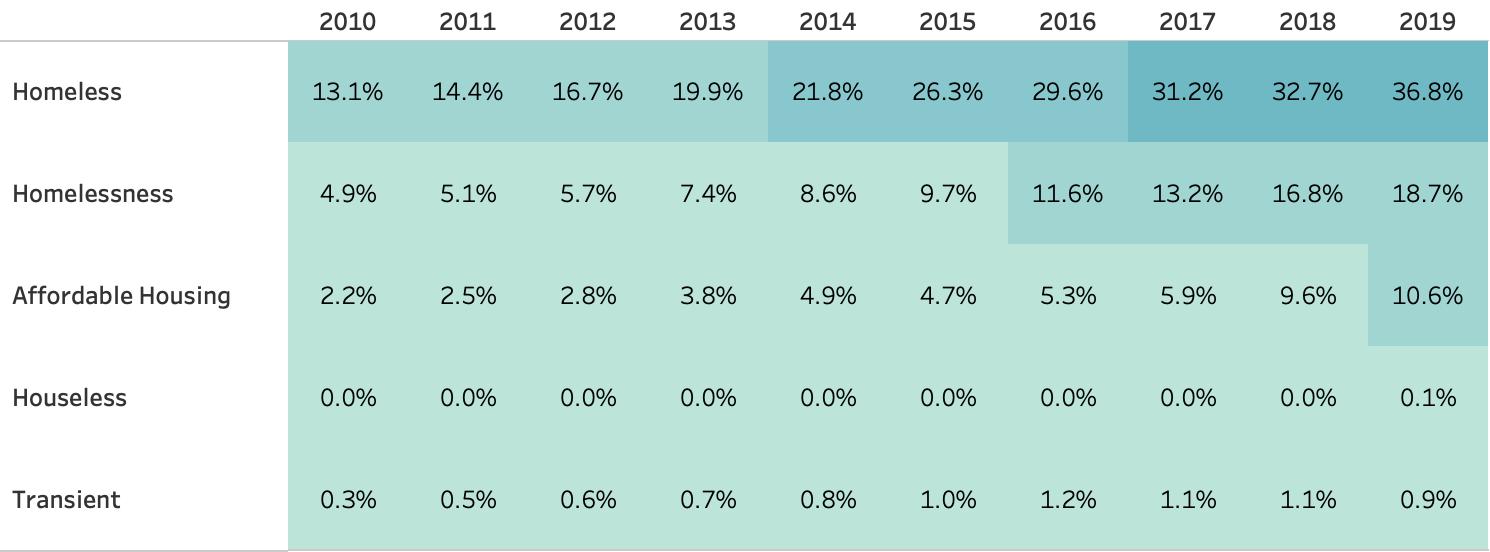


Percentage of hospital reporting entities with one or more uses of phrases in the *homelessness* theme by year.

**sFigure 19. Tobacco Theme**


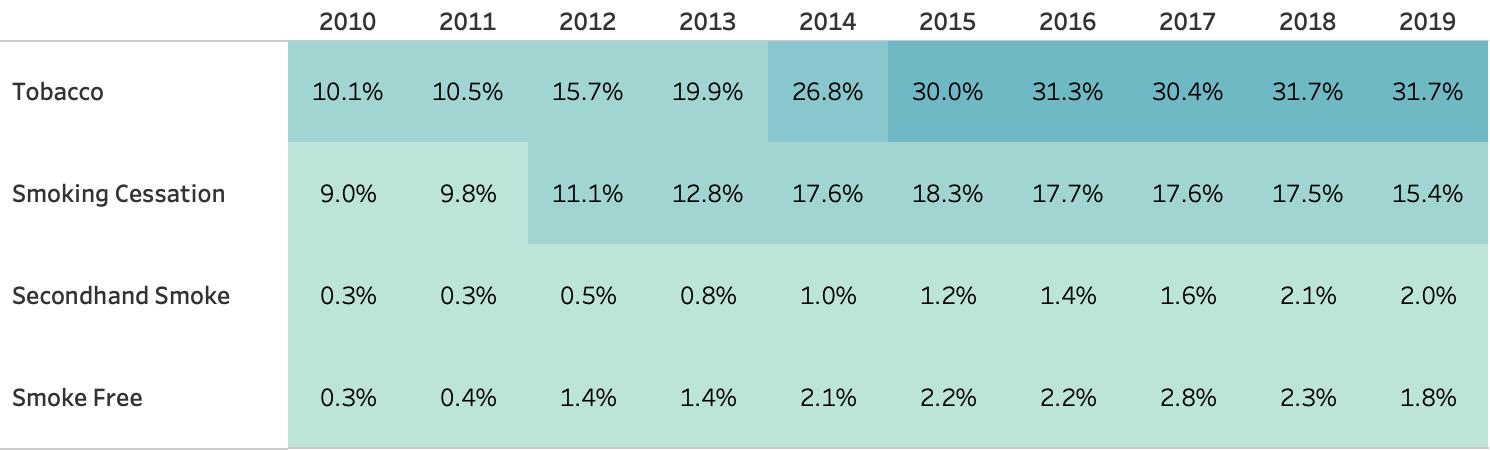


Percentage of hospital reporting entities with one or more uses of phrases in the *tobacco* theme by year.

**sFigure 20. Criminal Justice Theme**


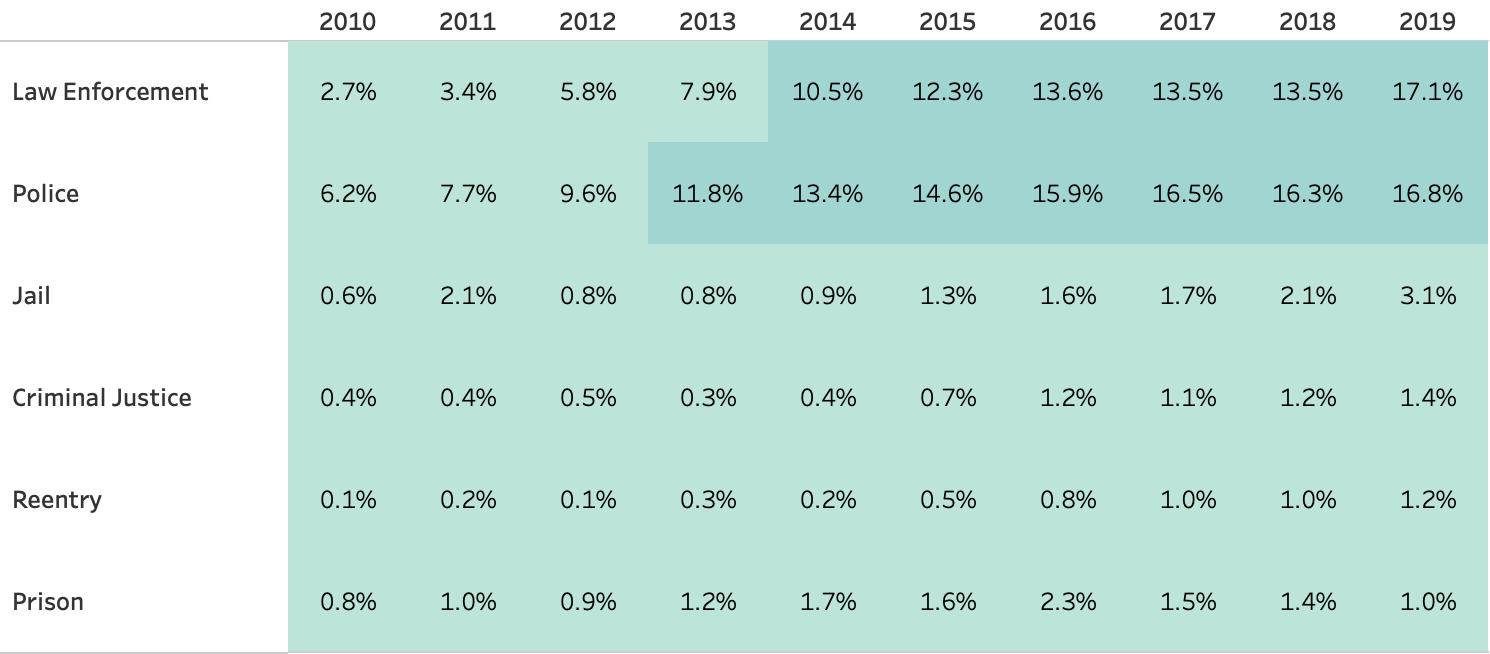


Percentage of hospital reporting entities with one or more uses of phrases in the *criminal justice* theme by year.

**sFigure 21. SDOH Theme**


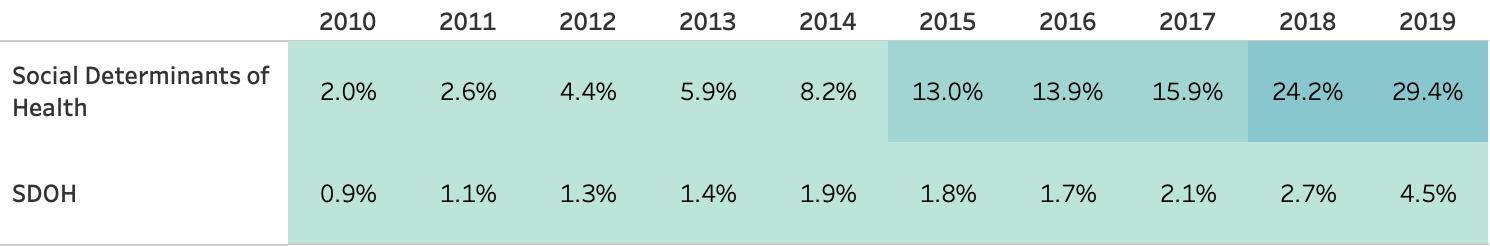


Percentage of hospital reporting entities with one or more uses of phrases in the *SDOH* theme by year.

**sFigure 22. Health IT Theme**


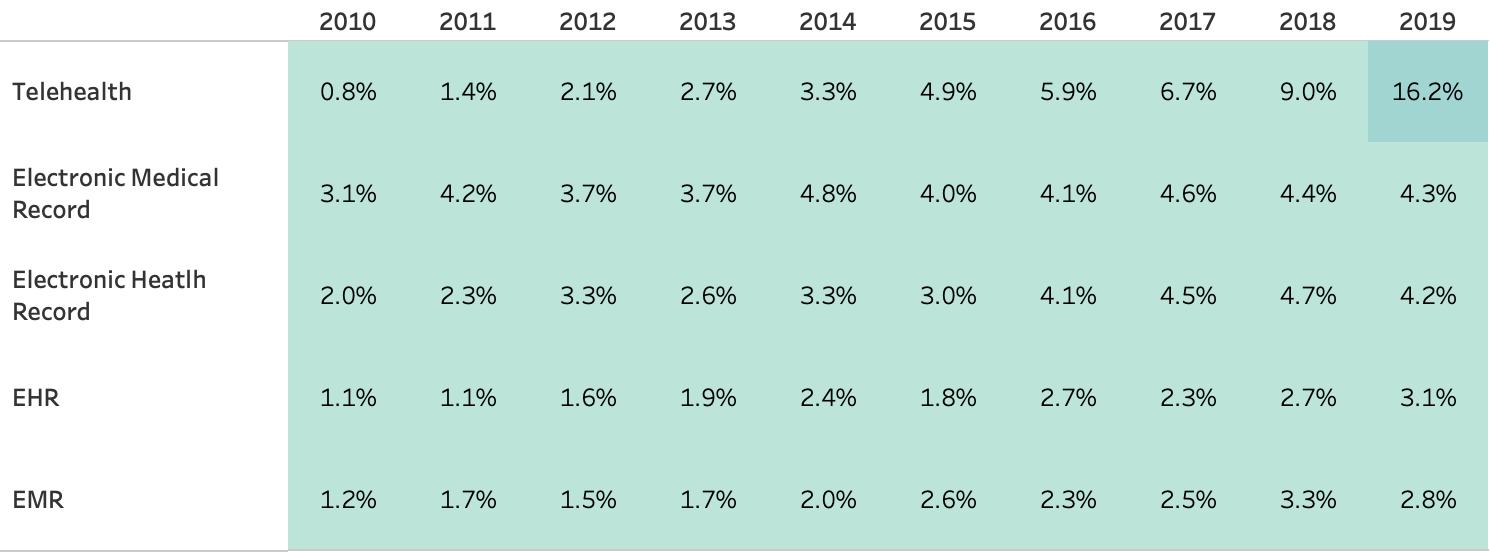


Percentage of hospital reporting entities with one or more uses of phrases in the *health IT* theme by year.

**sFigure 23. Sexual Health Theme**


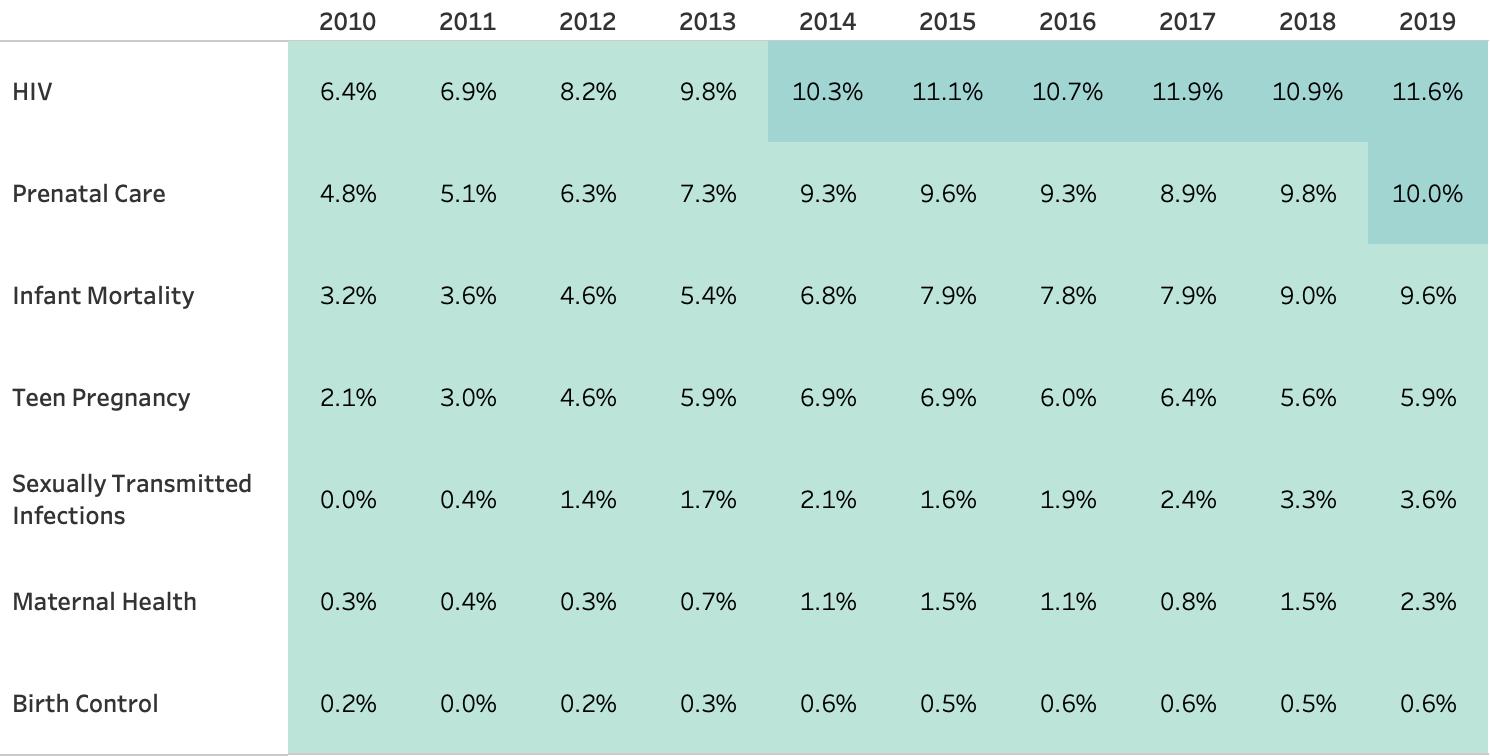


Percentage of hospital reporting entities with one or more uses of phrases in the *sexual health* theme by year.

**sFigure 24. Disability Theme**


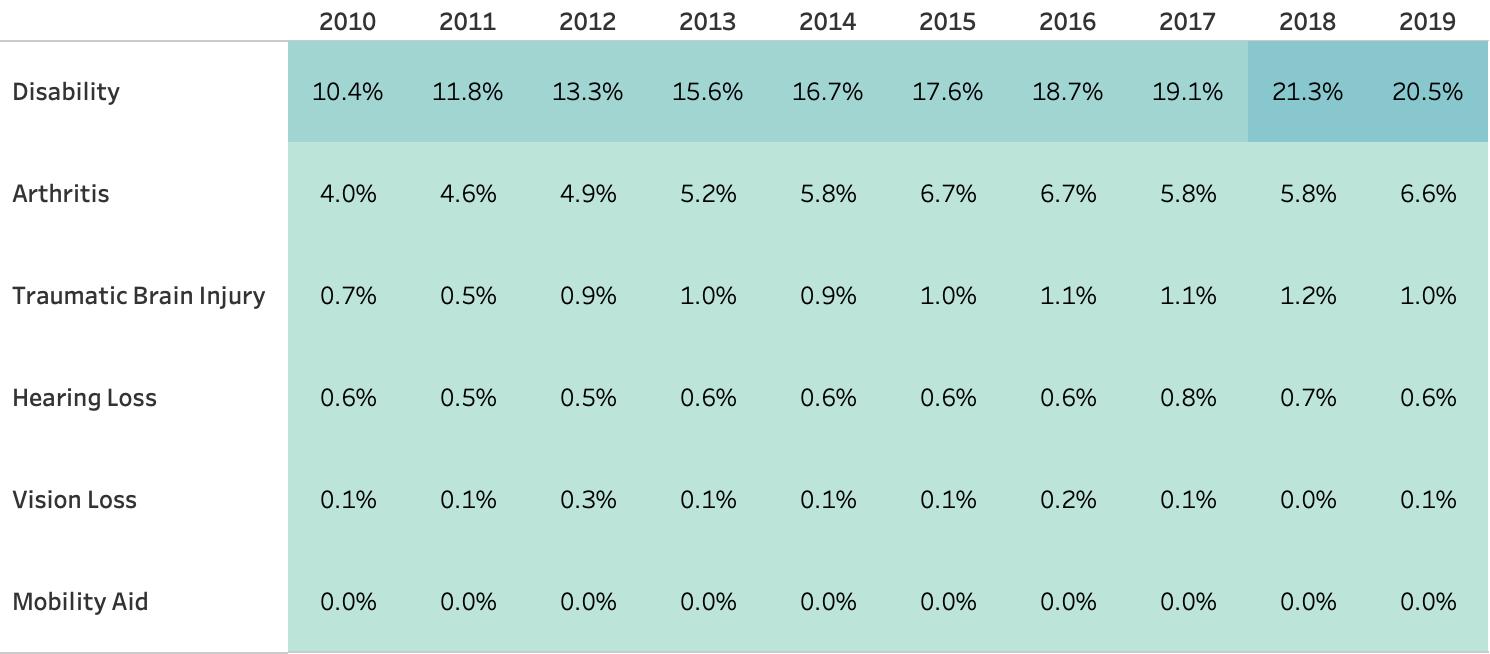


Percentage of hospital reporting entities with one or more uses of phrases in the *disability* theme by year.

**sFigure 25. Language Theme**


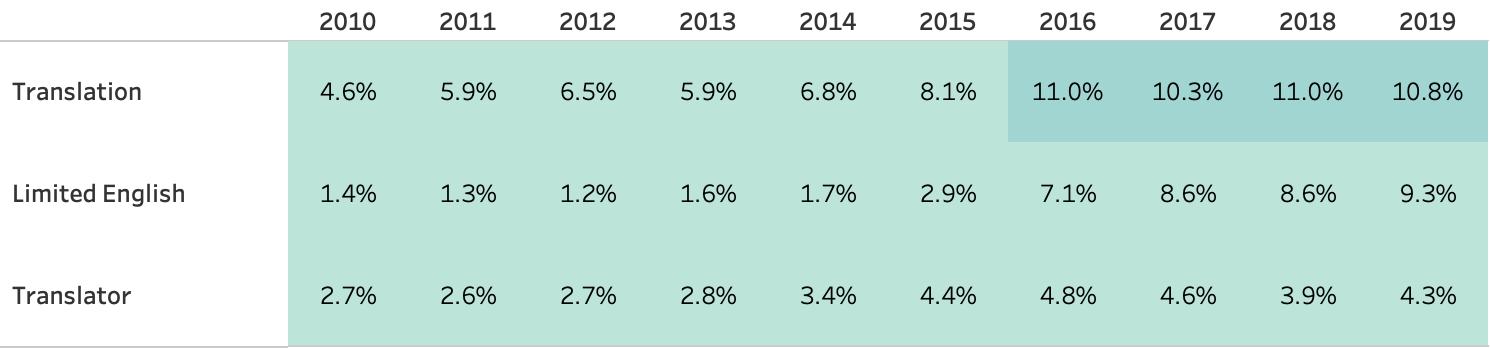


Percentage of hospital reporting entities with one or more uses of phrases in the *language* theme by year.

**sFigure 26. Firearms Theme**


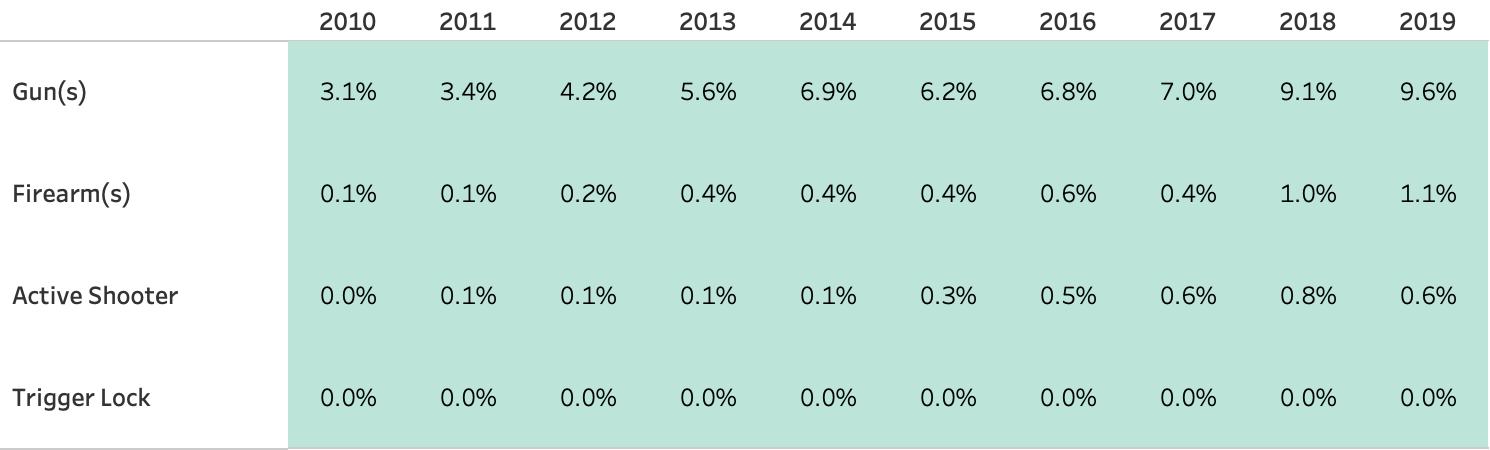


Percentage of hospital reporting entities with one or more uses of phrases in the *firearms* theme by year.

**sFigure 27. Immigration Theme**


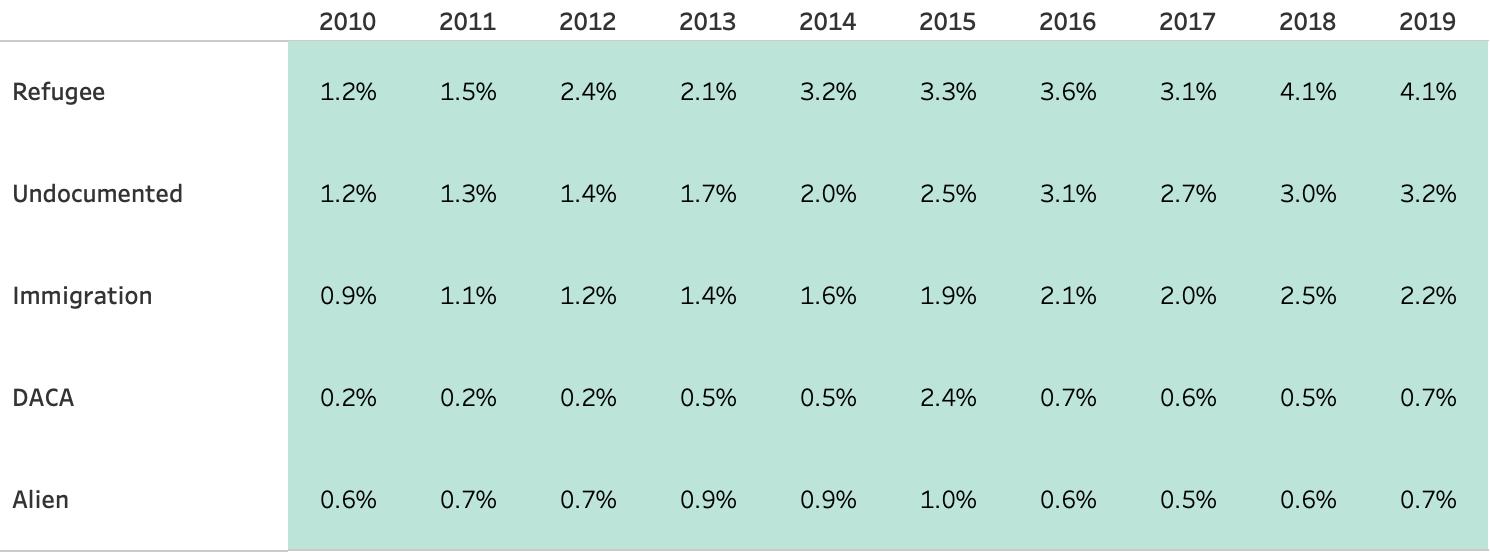


Percentage of hospital reporting entities with one or more uses of phrases in the *immigration* theme by year.

**sFigure 28. LGBTQ Theme**


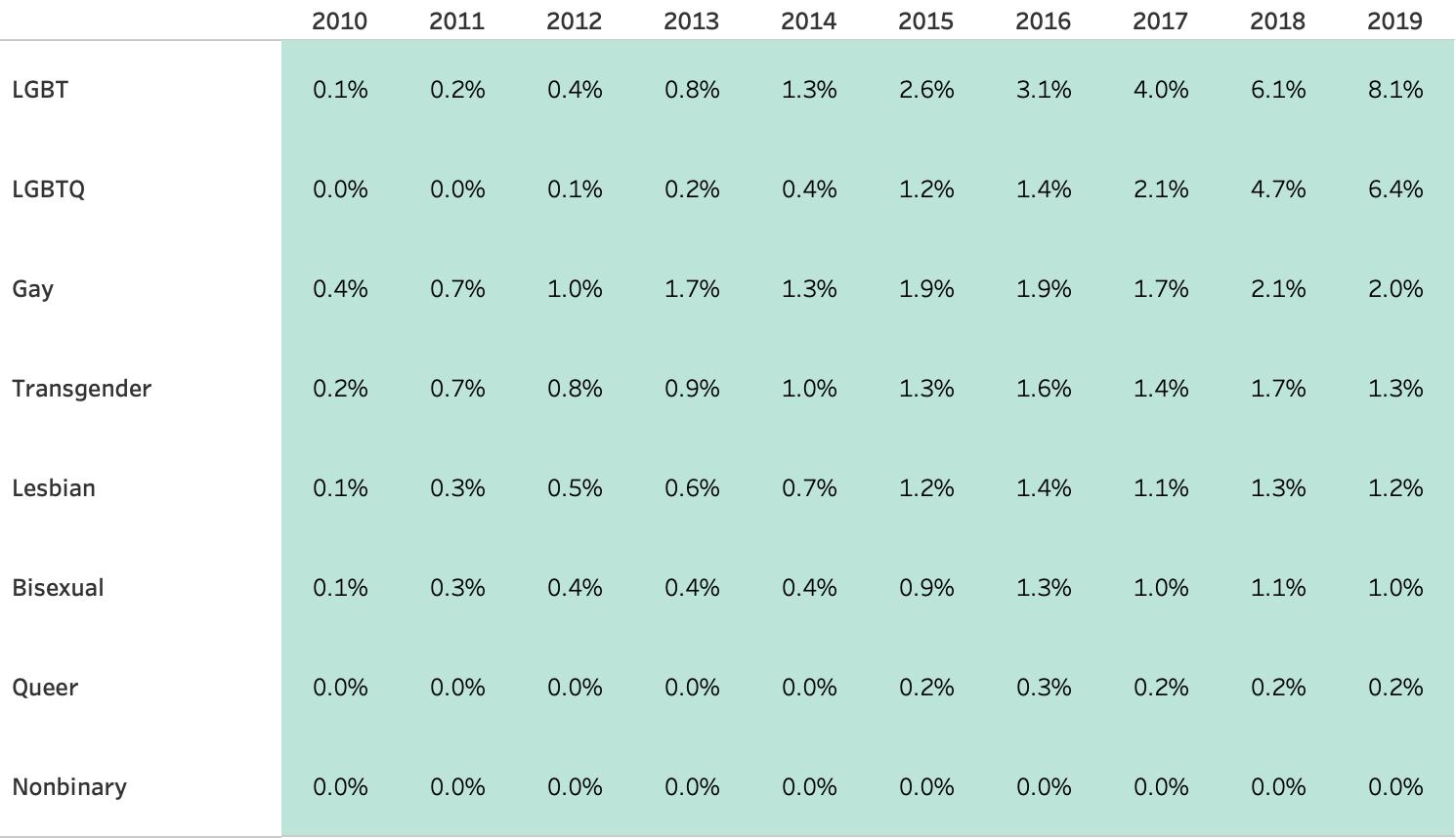


Percentage of hospital reporting entities with one or more uses of phrases in the *LGBTQ* theme by year.

**sFigure 29. Environment Theme**


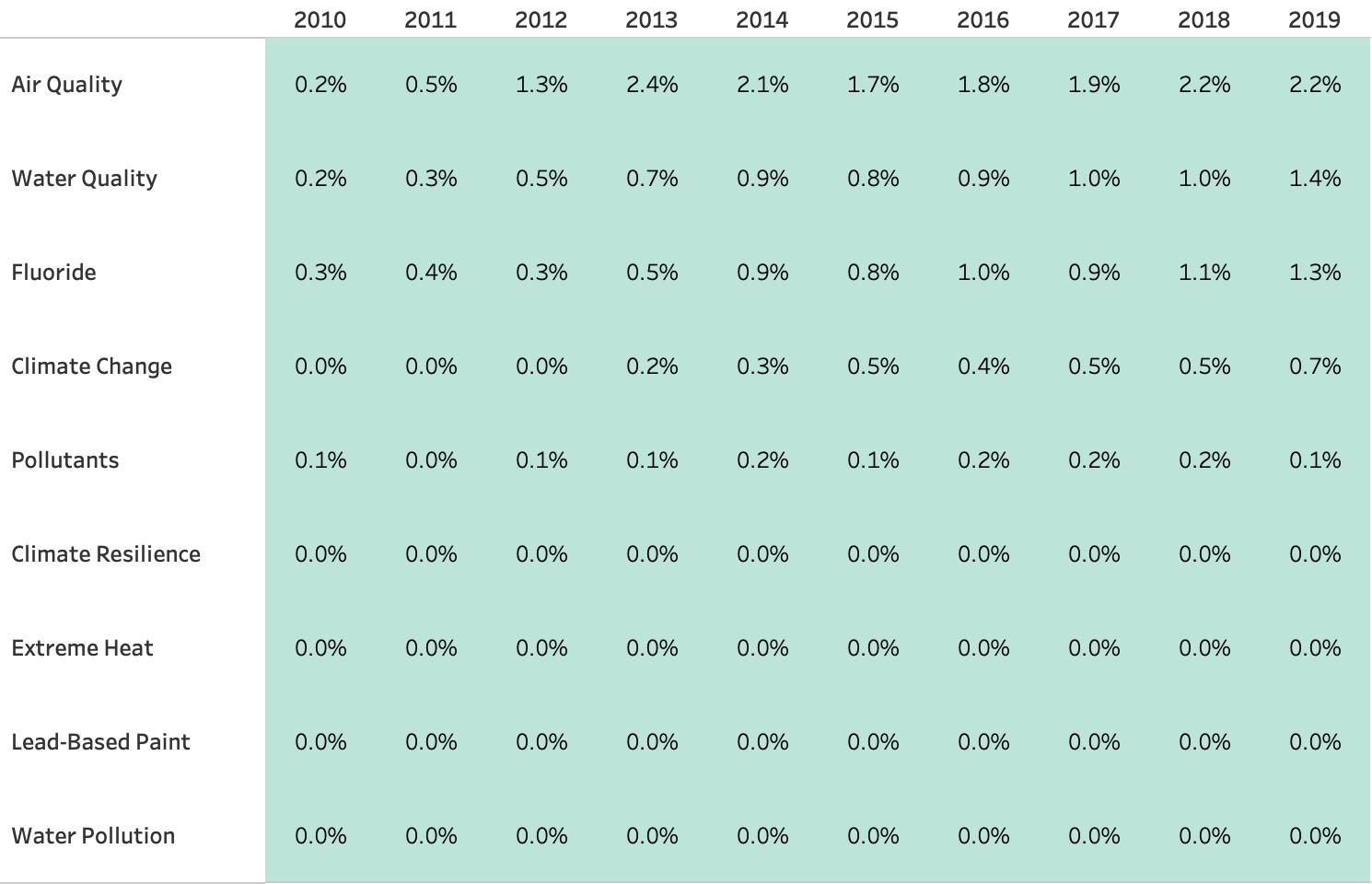


Percentage of hospital reporting entities with one or more uses of phrases in the *environment* theme by year.
