# Supplementary material for: Text Analysis of Trends in Health Equity and Disparities From the Internal Revenue Service Tax Documentation Submitted by US Nonprofit Hospitals Between 2010 and 2019: Exploratory Study
Source: J Med Internet Res. 2023 May 24;25:e44330. doi: 10.2196/44330 (PMC10248774; doi:10.2196/44330)
Supplement: Multimedia Appendix 4 [file jmir_v25i1e44330_app4.docx]

**S2.2 Detailed Geographic Variation from 2010 – 2019 for Each Theme**

The following figures provide additional detail of geographic variation for each theme by illustrating the percent of hospital reporting entities by state with one or more uses of a particular theme. A table of counts of number of reporting hospital entities by year is below.

**sTable 2 Number of Reporting Hospital Entities by State from 2010 – 2019**

|  | **Tax Year** | | | | | | | | | |
| --- | --- | --- | --- | --- | --- | --- | --- | --- | --- | --- |
| **State** | **2010** | **2011** | **2012** | **2013** | **2014** | **2015** | **2016** | **2017** | **2018** | **2019** |
| **AK** | 5 | 5 | 5 | 5 | 5 | 5 | 4 | 5 | 5 | 5 |
| **AL** | 20 | 20 | 19 | 17 | 16 | 16 | 15 | 16 | 16 | 12 |
| **AR** | 40 | 40 | 41 | 40 | 39 | 38 | 40 | 41 | 43 | 36 |
| **AZ** | 31 | 32 | 31 | 29 | 26 | 24 | 23 | 22 | 21 | 15 |
| **CA** | 113 | 111 | 112 | 111 | 113 | 114 | 112 | 109 | 103 | 79 |
| **CO** | 26 | 26 | 26 | 25 | 24 | 24 | 24 | 24 | 24 | 24 |
| **CT** | 31 | 31 | 31 | 31 | 30 | 30 | 27 | 27 | 28 |  |
| **DC** | 6 | 6 | 6 | 6 | 6 | 6 | 6 | 6 | 6 | 5 |
| **DE** | 5 | 5 | 5 | 5 | 5 | 5 | 5 | 5 | 5 | 5 |
| **FL** | 69 | 68 | 69 | 70 | 66 | 66 | 69 | 68 | 70 | 39 |
| **GA** | 68 | 68 | 65 | 66 | 64 | 66 | 64 | 68 | 65 | 53 |
| **HI** | 7 | 7 | 7 | 7 | 7 | 7 | 7 | 7 | 6 | 7 |
| **IA** | 46 | 46 | 46 | 46 | 46 | 47 | 46 | 47 | 46 | 45 |
| **ID** | 14 | 13 | 15 | 15 | 14 | 15 | 14 | 15 | 15 | 7 |
| **IL** | 110 | 111 | 114 | 113 | 111 | 106 | 99 | 102 | 98 | 76 |
| **IN** | 54 | 57 | 63 | 64 | 64 | 65 | 64 | 61 | 62 | 57 |
| **KS** | 55 | 54 | 53 | 52 | 51 | 50 | 49 | 48 | 48 | 35 |
| **KY** | 52 | 52 | 52 | 51 | 50 | 48 | 46 | 45 | 43 | 30 |
| **LA** | 27 | 26 | 26 | 30 | 31 | 32 | 33 | 32 | 31 | 13 |
| **MA** | 51 | 44 | 42 | 41 | 41 | 40 | 39 | 39 | 37 | 1 |
| **MD** | 43 | 43 | 42 | 44 | 44 | 45 | 45 | 45 | 45 | 43 |
| **ME** | 34 | 34 | 34 | 32 | 32 | 31 | 31 | 31 | 31 | 13 |
| **MI** | 104 | 98 | 98 | 85 | 81 | 86 | 86 | 88 | 87 | 67 |
| **MN** | 77 | 78 | 80 | 78 | 75 | 72 | 65 | 69 | 69 | 60 |
| **MO** | 51 | 52 | 52 | 52 | 53 | 53 | 53 | 55 | 56 | 47 |
| **MS** | 25 | 25 | 28 | 29 | 29 | 30 | 29 | 29 | 30 | 12 |
| **MT** | 44 | 44 | 45 | 45 | 45 | 44 | 47 | 48 | 47 | 46 |
| **NC** | 60 | 58 | 58 | 58 | 53 | 54 | 51 | 50 | 50 | 26 |
| **ND** | 39 | 39 | 37 | 37 | 37 | 37 | 37 | 37 | 35 | 30 |
| **NE** | 43 | 43 | 43 | 43 | 44 | 44 | 44 | 45 | 46 | 40 |
| **NH** | 23 | 23 | 23 | 23 | 23 | 23 | 23 | 22 | 21 | 9 |
| **NJ** | 52 | 50 | 48 | 48 | 45 | 43 | 39 | 38 | 36 | 25 |
| **NM** | 9 | 8 | 9 | 9 | 9 | 9 | 9 | 9 | 9 | 9 |
| **NV** | 7 | 7 | 6 | 7 | 7 | 7 | 6 | 6 | 6 | 6 |
| **NY** | 158 | 156 | 154 | 154 | 149 | 149 | 146 | 144 | 141 | 123 |
| **OH** | 112 | 108 | 106 | 106 | 89 | 85 | 83 | 83 | 82 | 73 |
| **OK** | 32 | 35 | 34 | 35 | 36 | 33 | 34 | 34 | 35 | 23 |
| **OR** | 28 | 28 | 28 | 28 | 28 | 28 | 28 | 27 | 27 | 24 |
| **PA** | 126 | 127 | 121 | 119 | 118 | 107 | 105 | 109 | 104 | 98 |
| **RI** | 11 | 11 | 10 | 10 | 7 | 7 | 7 | 7 | 6 |  |
| **SC** | 19 | 19 | 20 | 20 | 19 | 20 | 21 | 20 | 20 | 6 |
| **SD** | 32 | 31 | 31 | 31 | 30 | 30 | 30 | 30 | 30 | 29 |
| **TN** | 46 | 45 | 38 | 37 | 38 | 41 | 41 | 41 | 41 | 32 |
| **TX** | 94 | 94 | 101 | 101 | 101 | 101 | 100 | 98 | 95 | 78 |
| **UT** | 5 | 4 | 4 | 5 | 5 | 5 | 5 | 5 | 5 | 4 |
| **VA** | 43 | 44 | 45 | 44 | 43 | 44 | 43 | 43 | 38 | 32 |
| **VT** | 14 | 14 | 14 | 14 | 14 | 14 | 14 | 14 | 14 |  |
| **WA** | 30 | 29 | 30 | 30 | 30 | 30 | 29 | 32 | 31 | 29 |
| **WI** | 101 | 101 | 101 | 101 | 101 | 95 | 93 | 92 | 89 | 71 |
| **WV** | 32 | 32 | 33 | 33 | 32 | 31 | 32 | 31 | 28 | 23 |
| **WY** | 3 | 3 | 3 | 3 | 3 | 3 | 3 | 4 | 4 | 4 |
| **No State Reported** | 1 | 1 | 1 | 2 | 4 | 1 | 1 | 1 | 1 | 1 |

**sFigure 30. Affordability Theme Geographic Variation from 2010 – 2019**


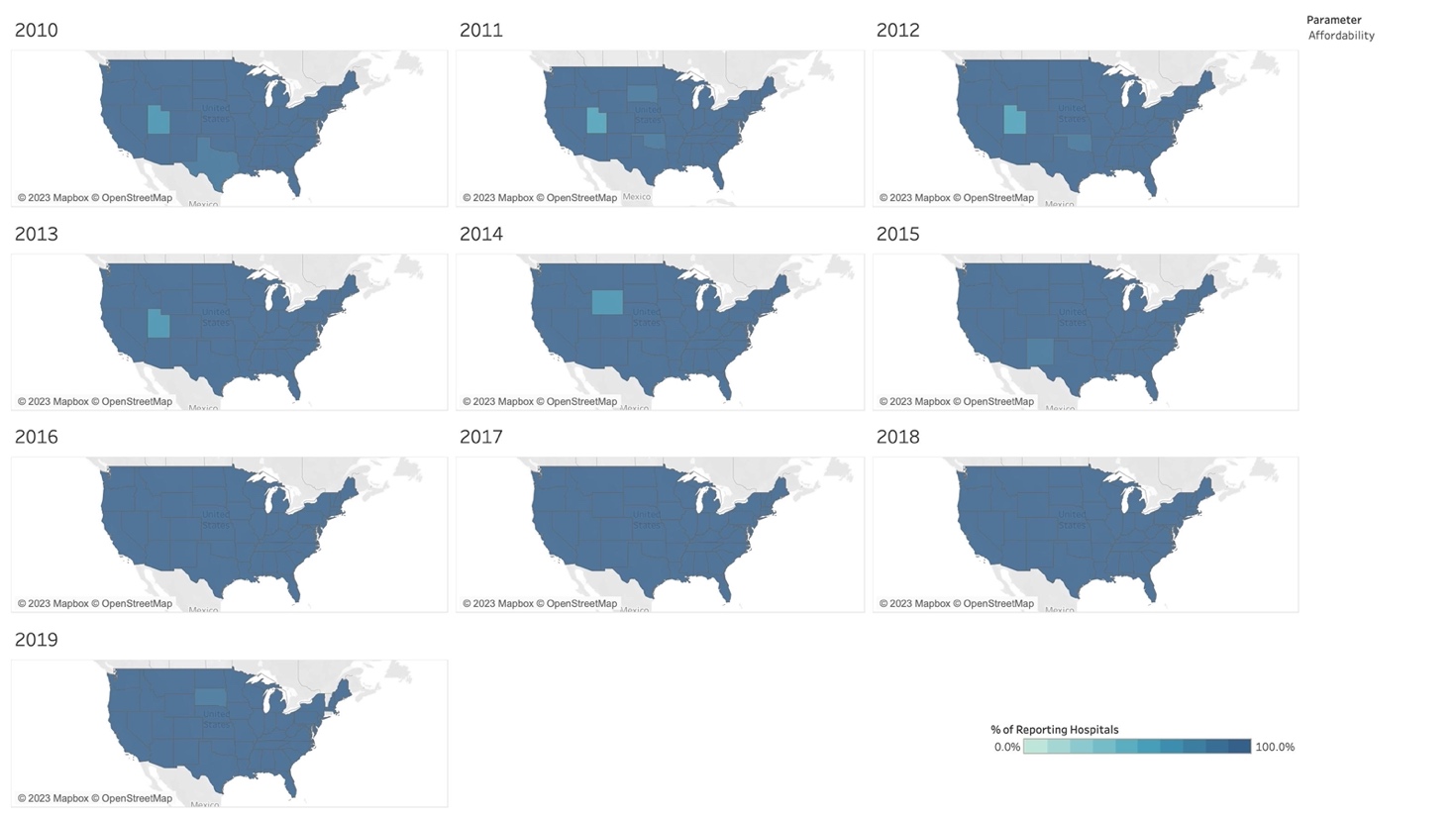


Percentage of hospital reporting entities in each state with one or more uses of phrases in the *affordability* theme by year.

**sFigure 31. Government Organizations Theme**


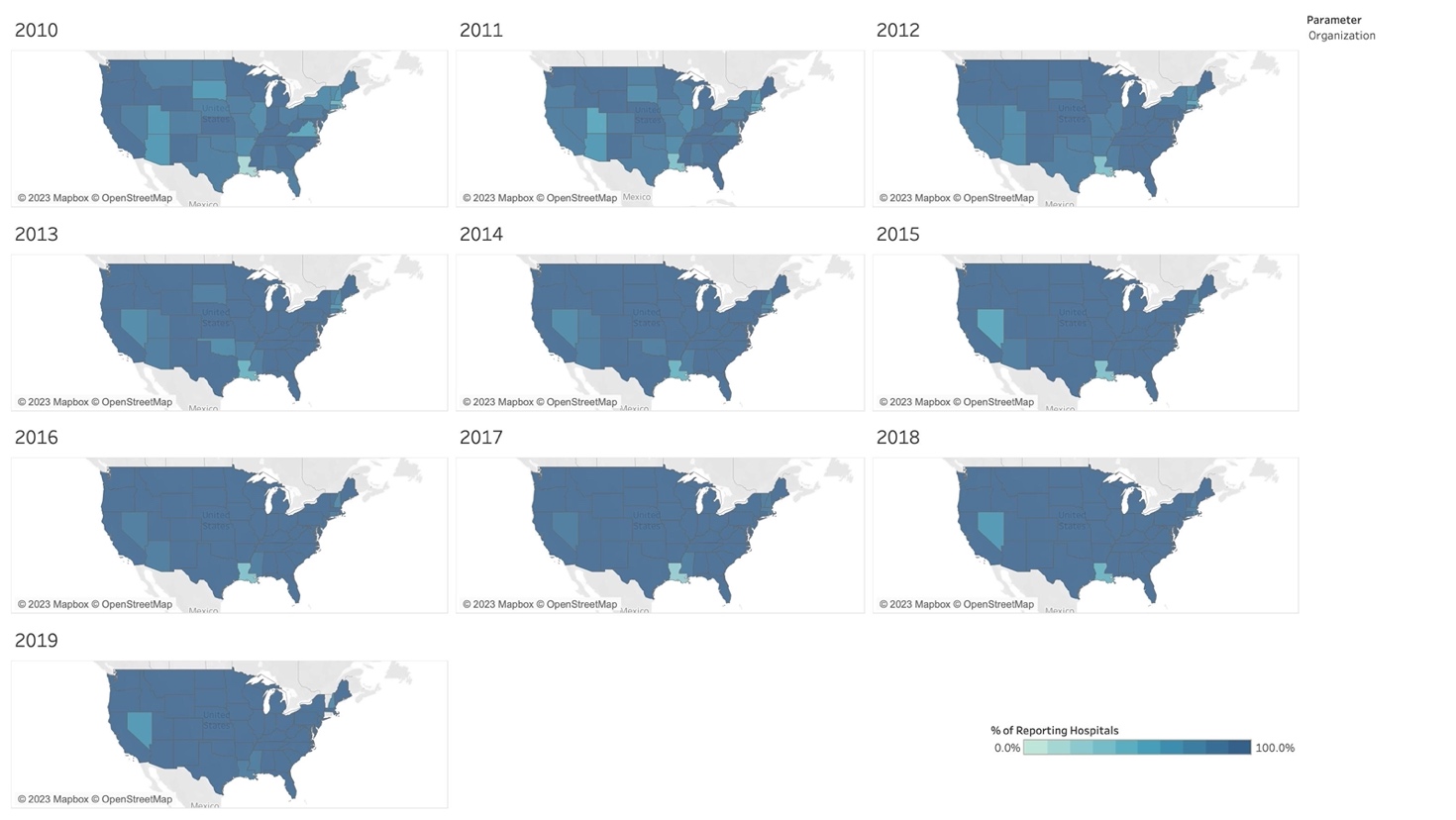


Percentage of hospital reporting entities in each state with one or more uses of phrases in the *government organizations* theme by year.

**sFigure 32. Data Collection Theme**


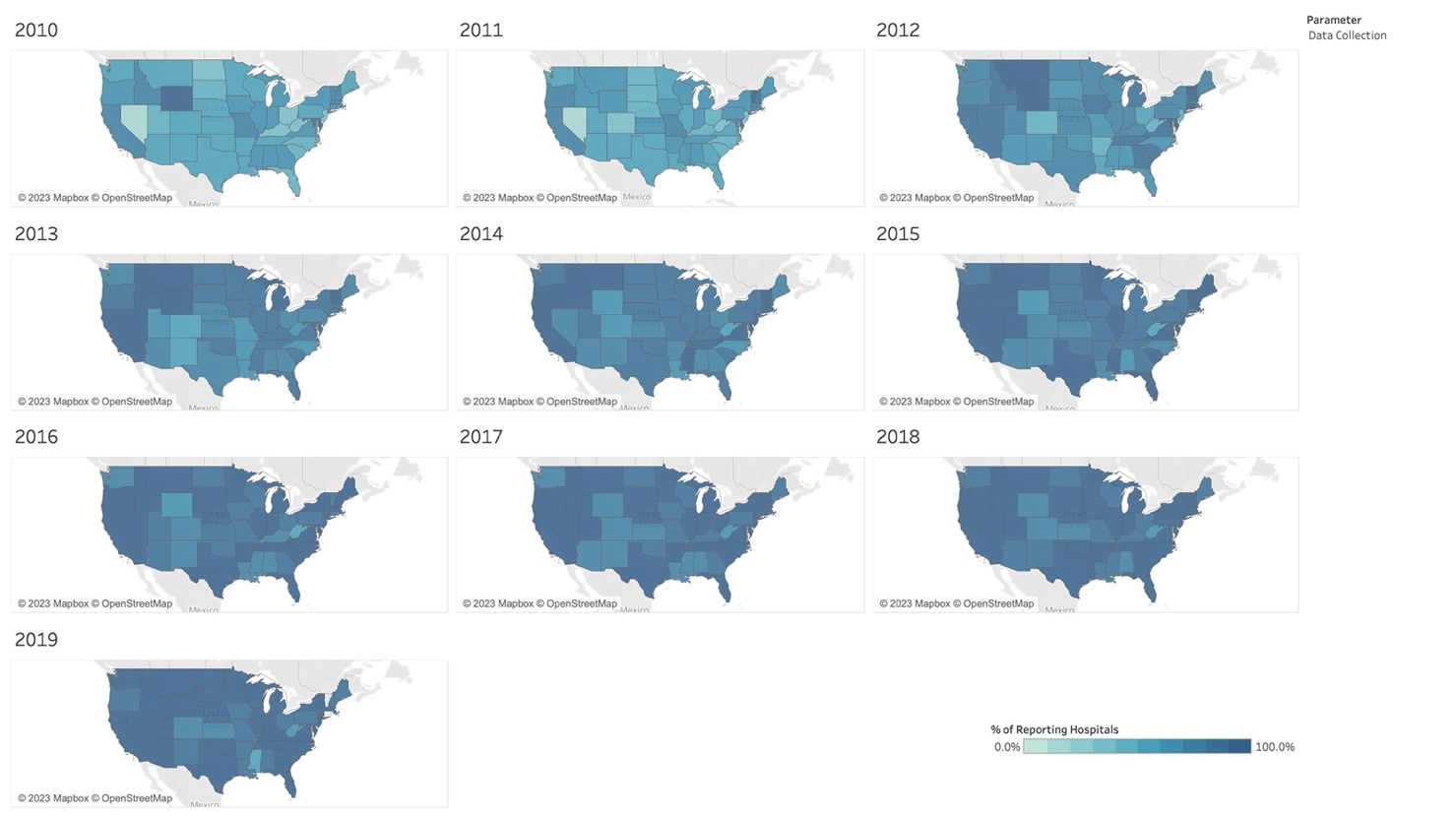


Percentage of hospital reporting entities in each state with one or more uses of phrases in the *data collection* theme by year.

**sFigure 33. Mental Health Theme**


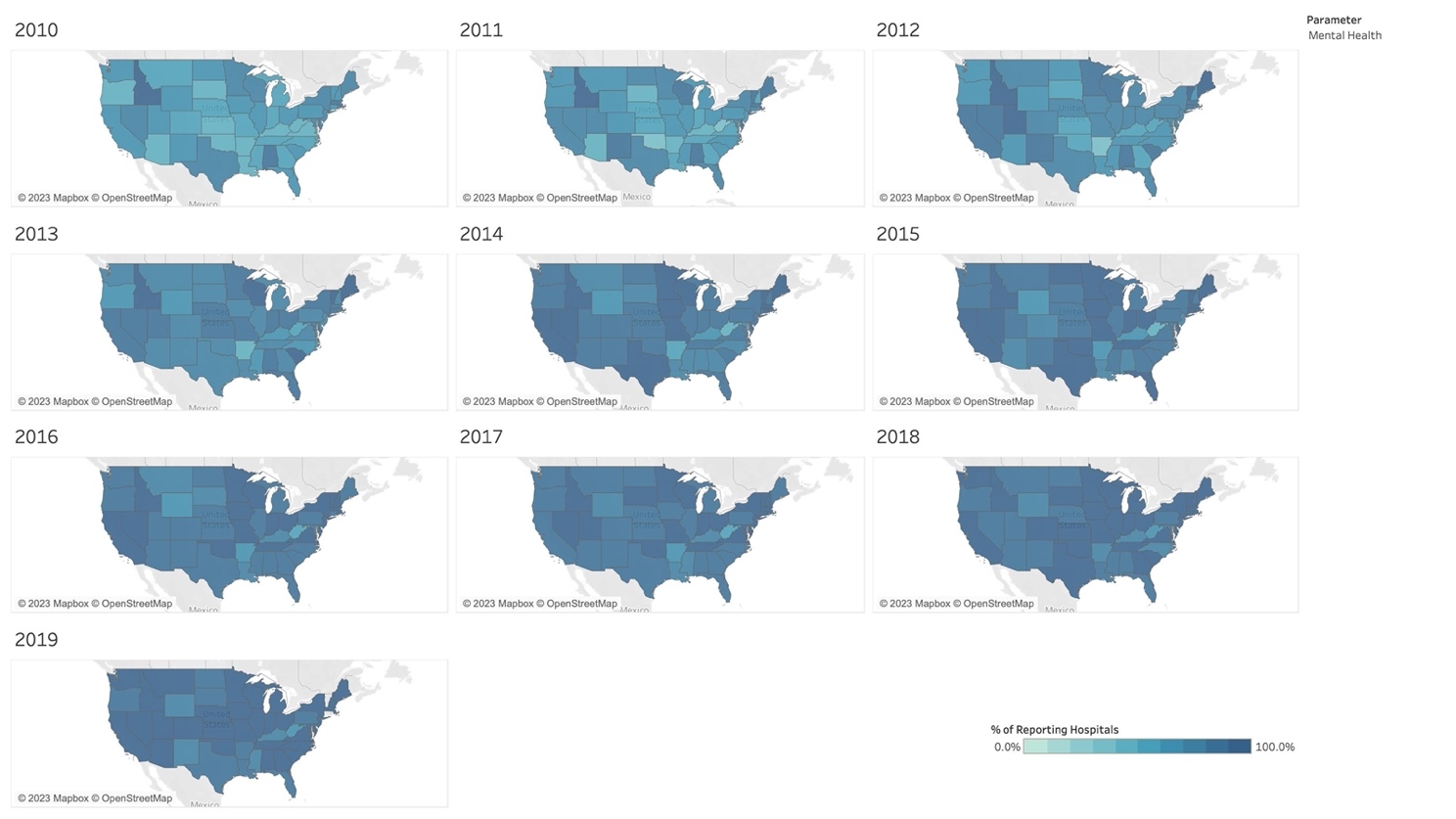


Percentage of hospital reporting entities in each state with one or more uses of phrases in the *mental health* theme by year.

**sFigure 34. Poverty Theme**


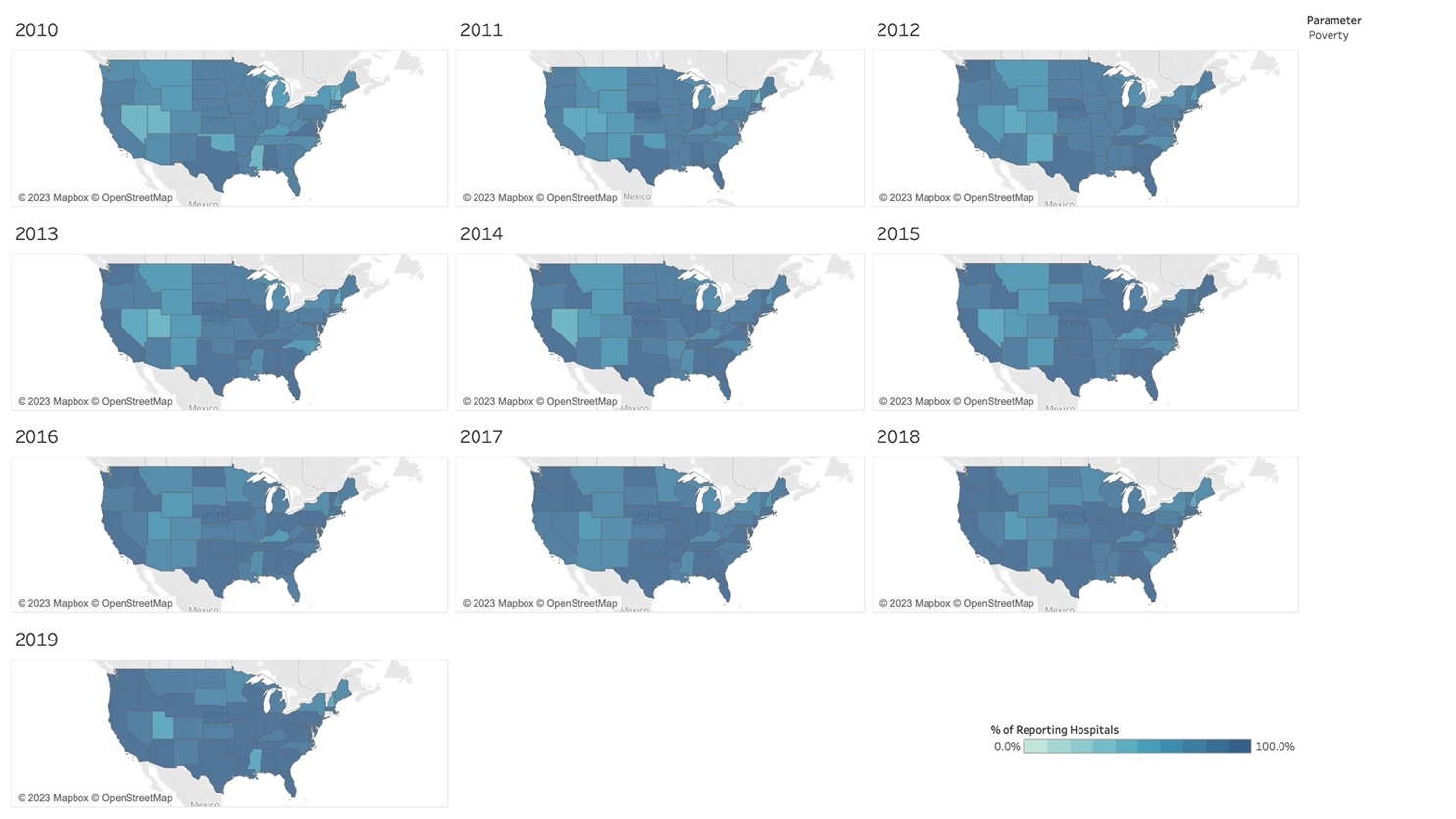


Percentage of hospital reporting entities in each state with one or more uses of phrases in the *poverty* theme by year.

**sFigure 35. Insurance Theme**


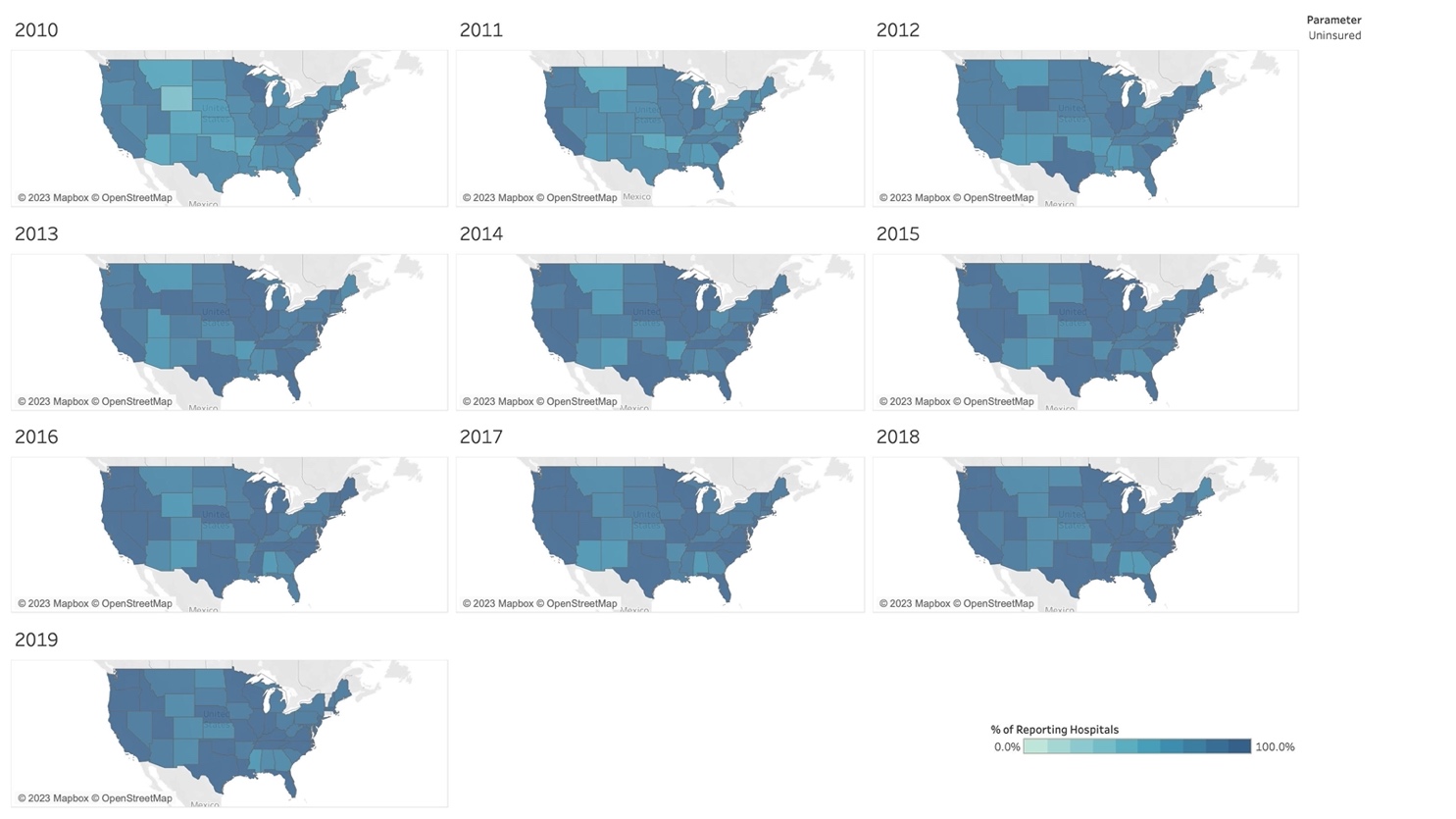


Percentage of hospital reporting entities in each state with one or more uses of phrases in the *insurance* theme by year.

**sFigure 36. Activity Theme**


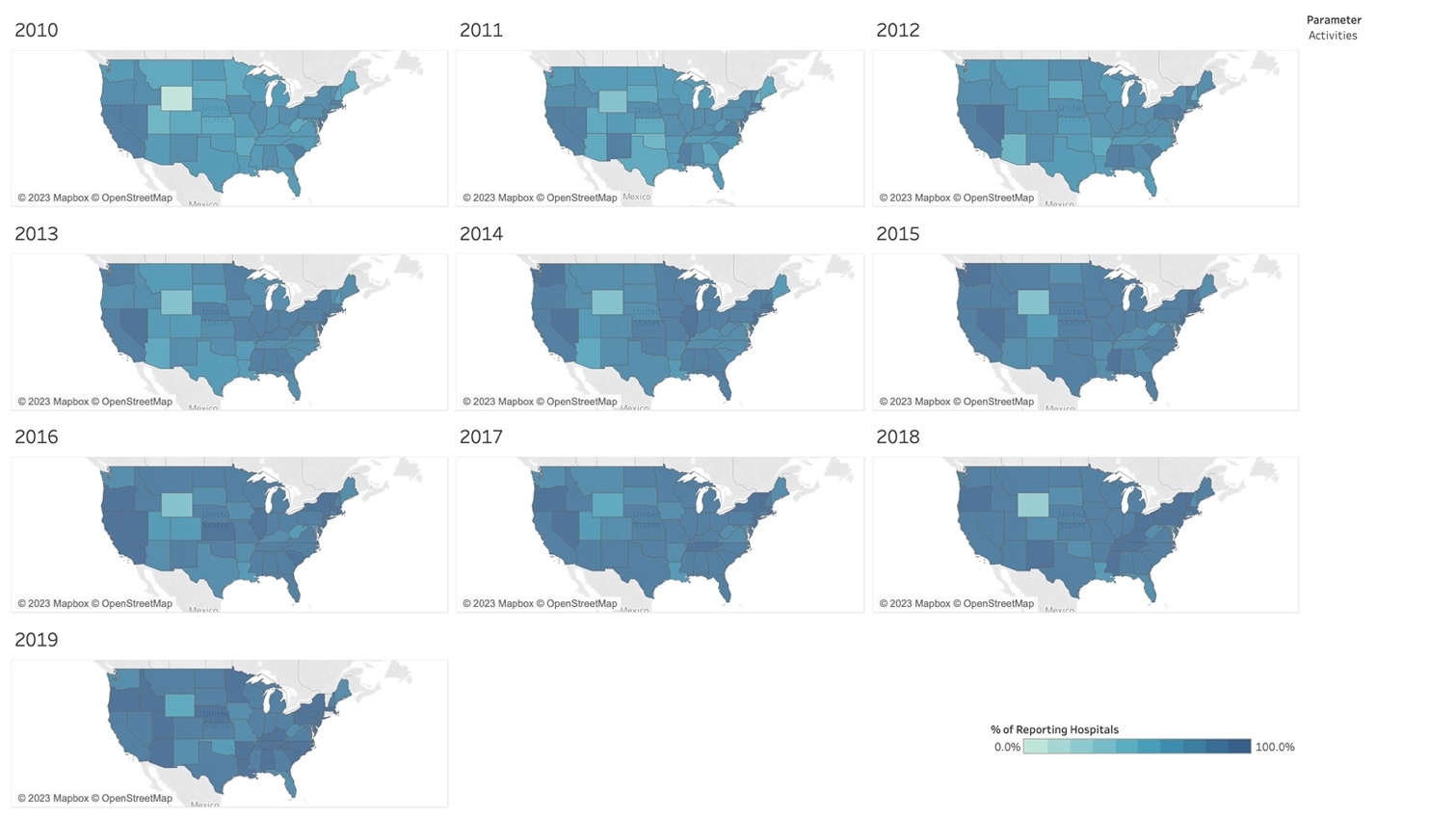


Percentage of hospital reporting entities in each state with one or more uses of phrases in the *activity* theme by year.

**sFigure 37. Chronic Illness Theme**


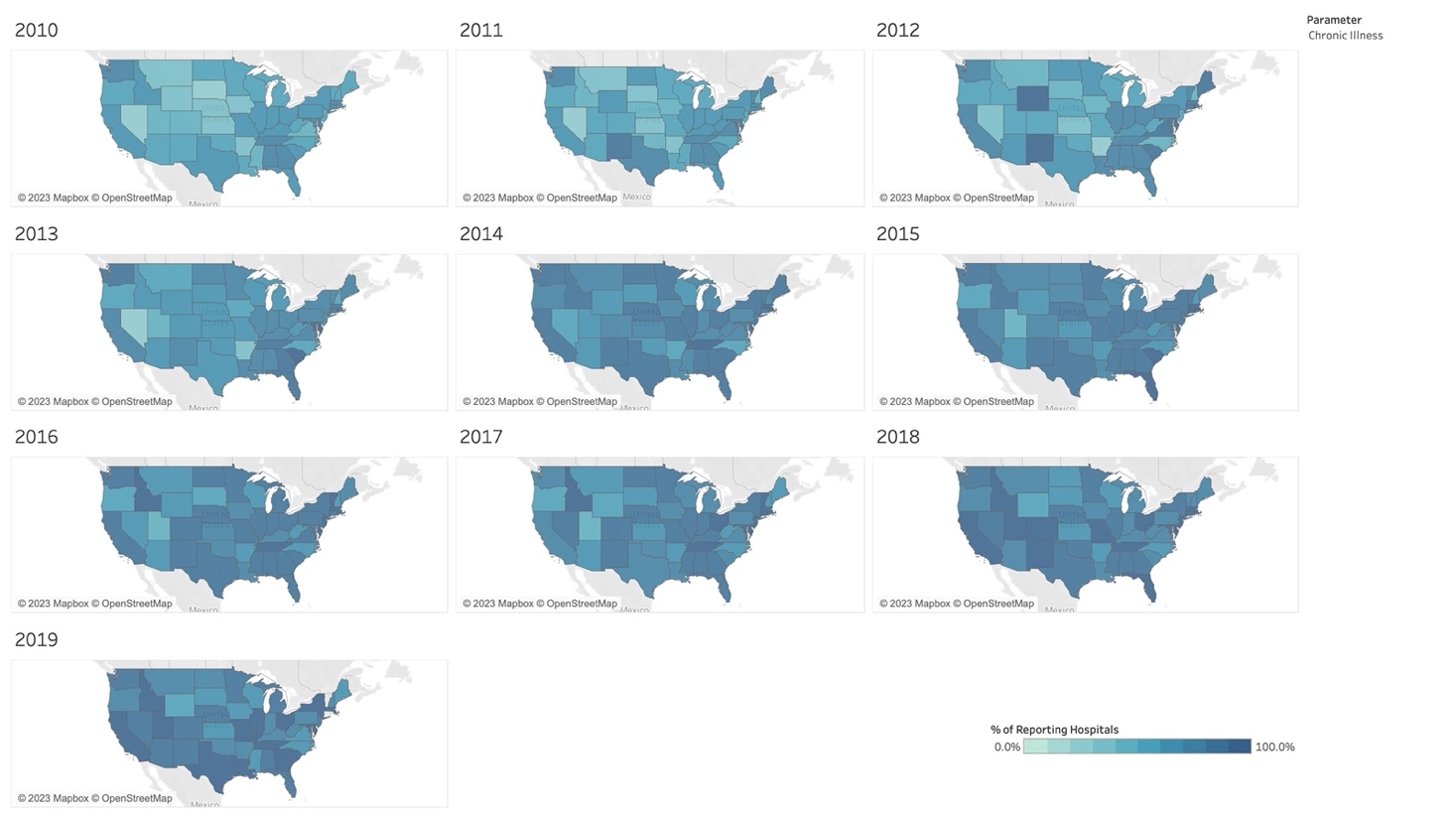


Percentage of hospital reporting entities by state with one or more uses of phrases in the *chronic illness* theme by year.

**sFigure 38. Child Theme**


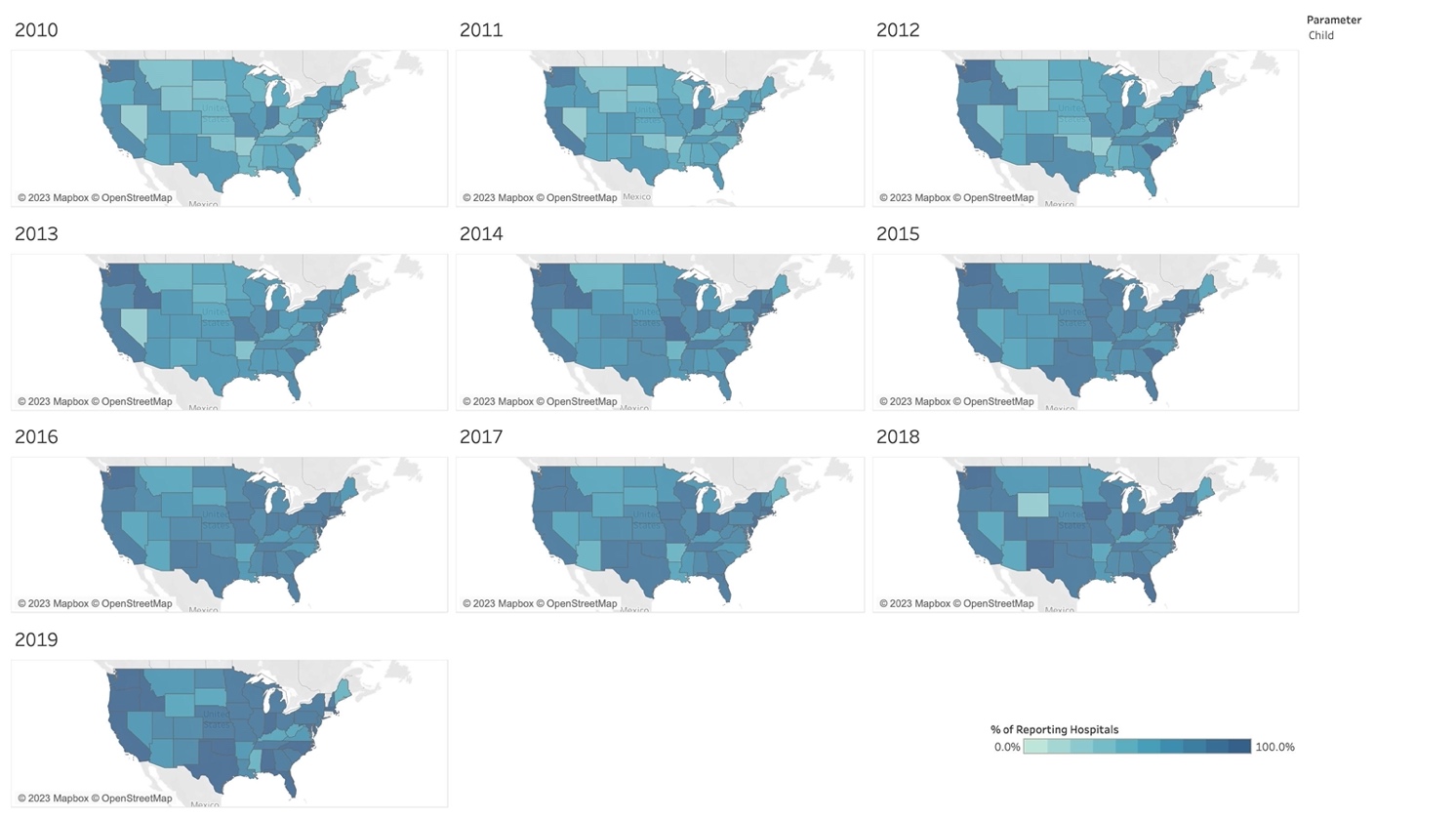


Percentage of hospital reporting entities by state with one or more uses of phrases in the *child* theme by year.

**sFigure 39. Senior Theme**


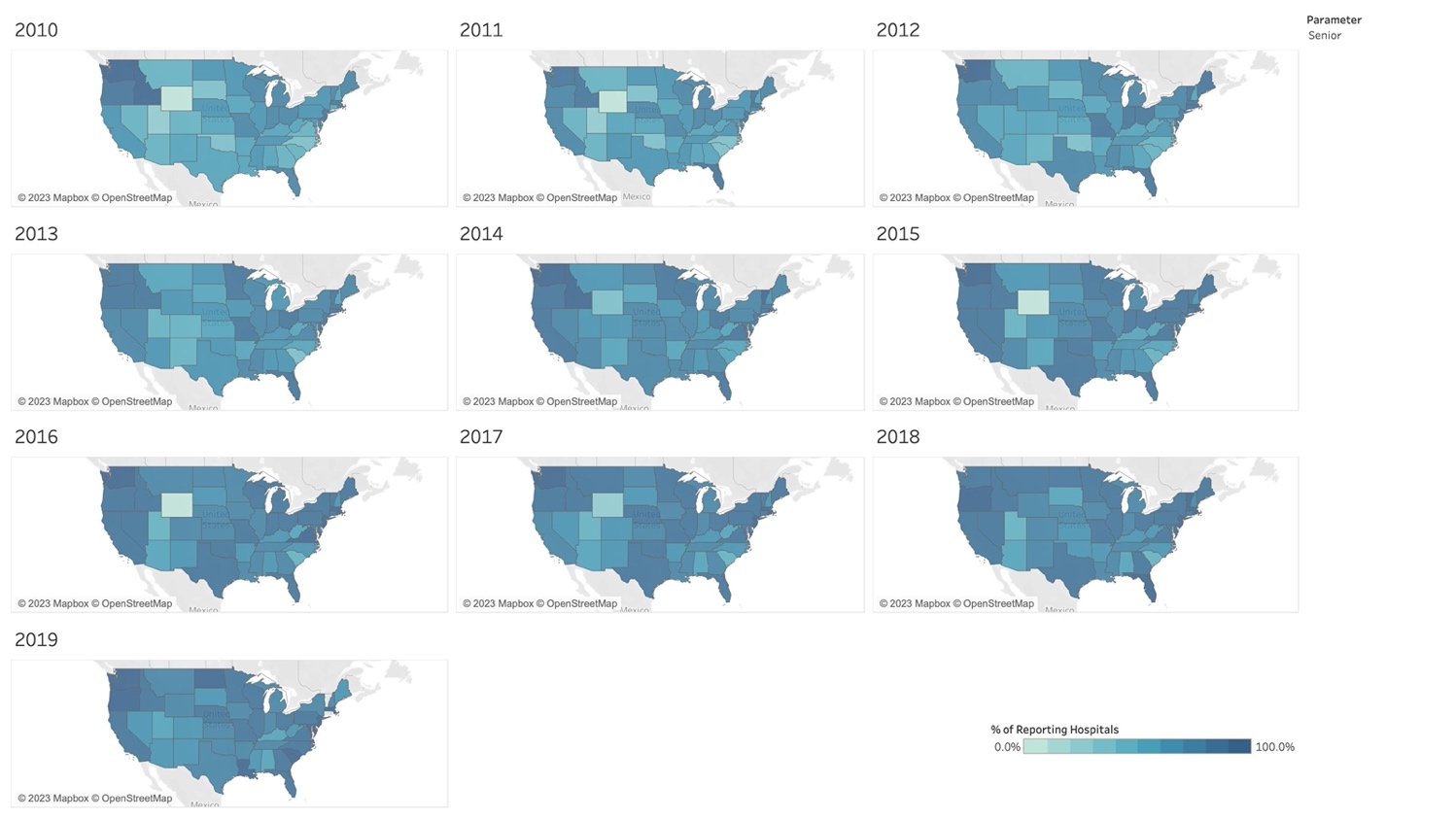


Percentage of hospital reporting entities by state with one or more uses of phrases in the *senior* theme by year.

**sFigure 40. Nutrition Theme**


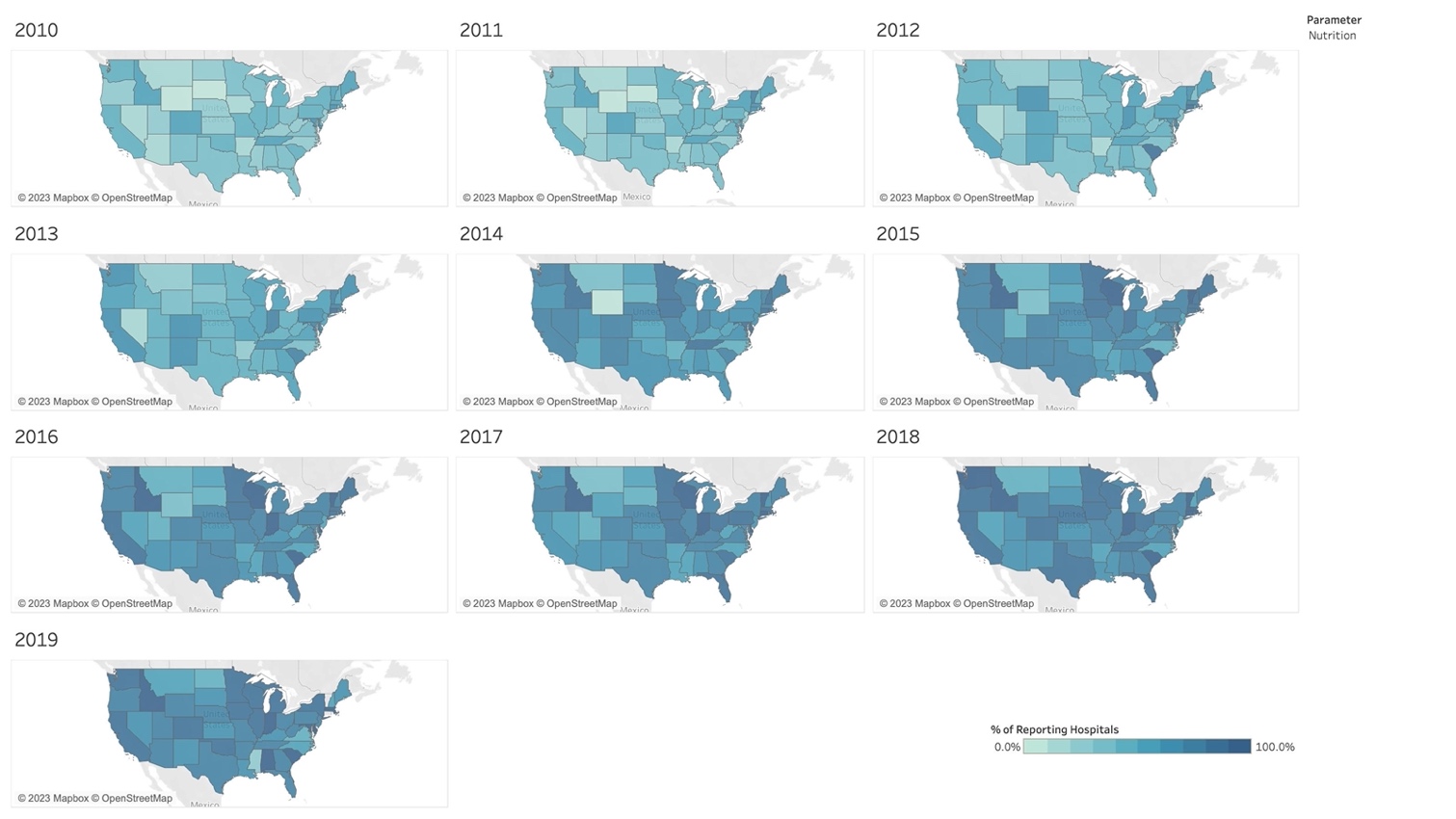


Percentage of hospital reporting entities by state with one or more uses of phrases in the *nutrition* theme by year.

**sFigure 41. Substance Use Theme**


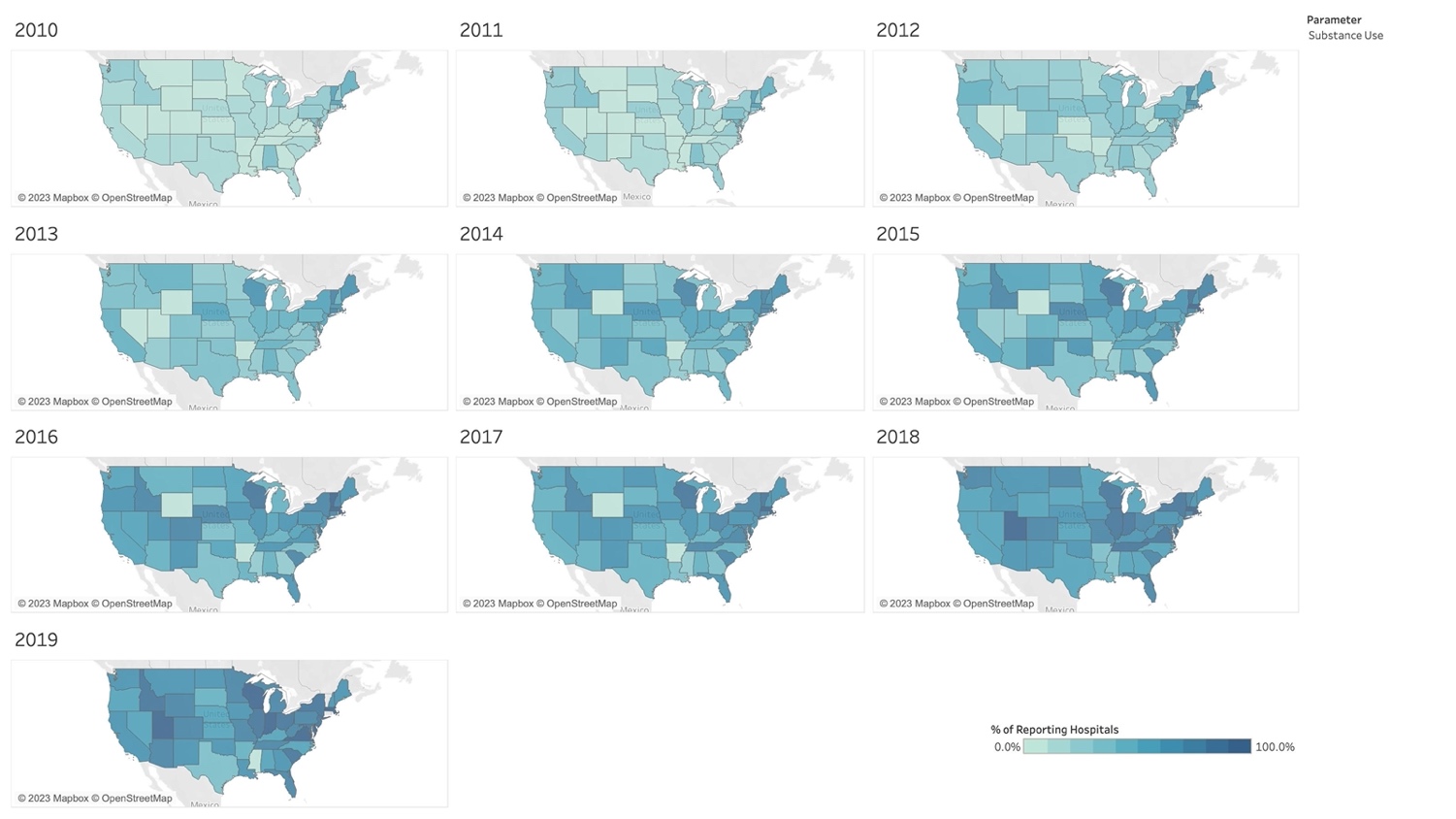


Percentage of hospital reporting entities by state with one or more uses of phrases in the *substance use* theme by year.

**sFigure 42. Oral Health Theme**


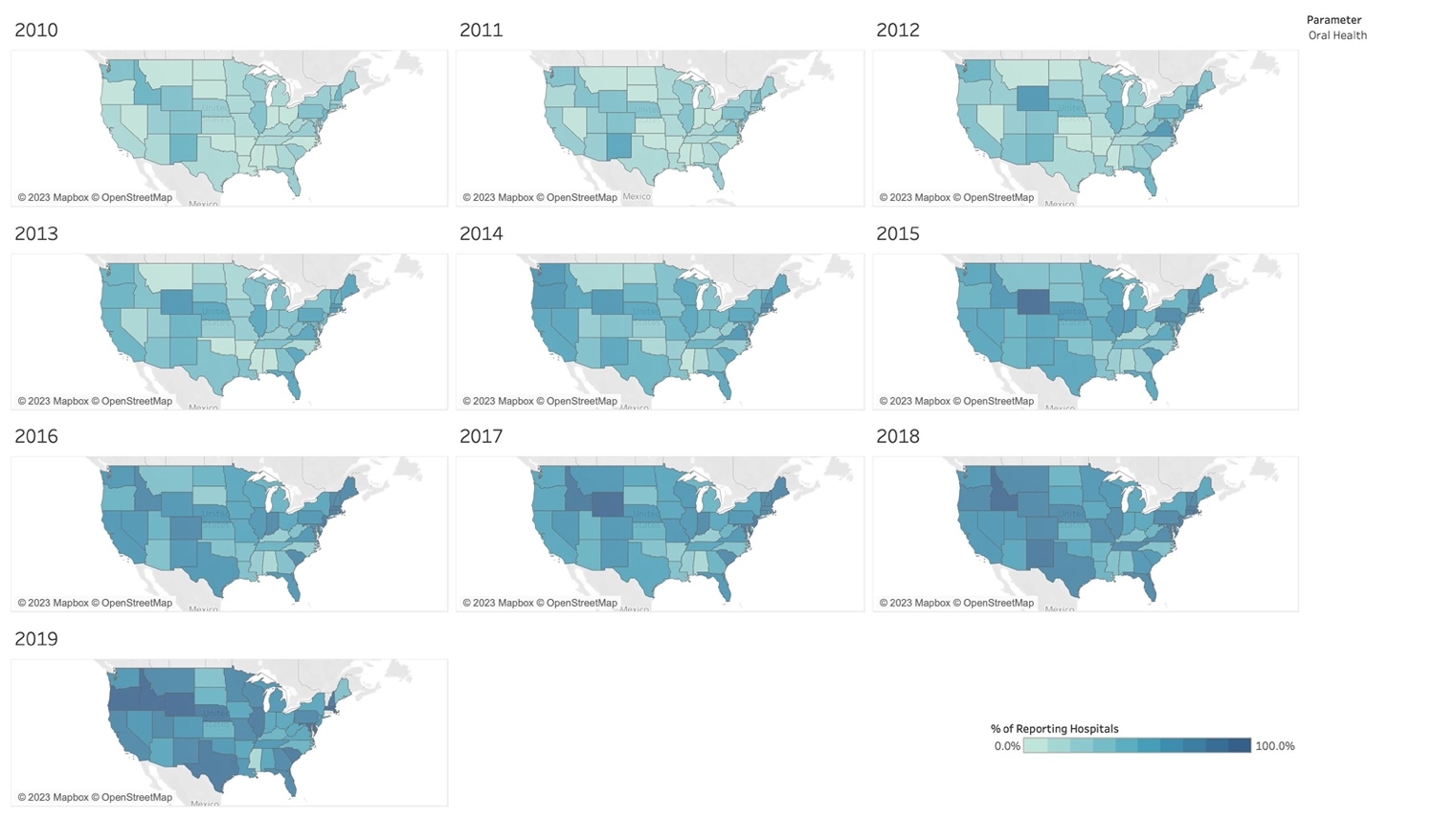


Percentage of hospital reporting entities by state with one or more uses of phrases in the *oral health* theme by year.

**sFigure 43. Race and Ethnicity Theme**


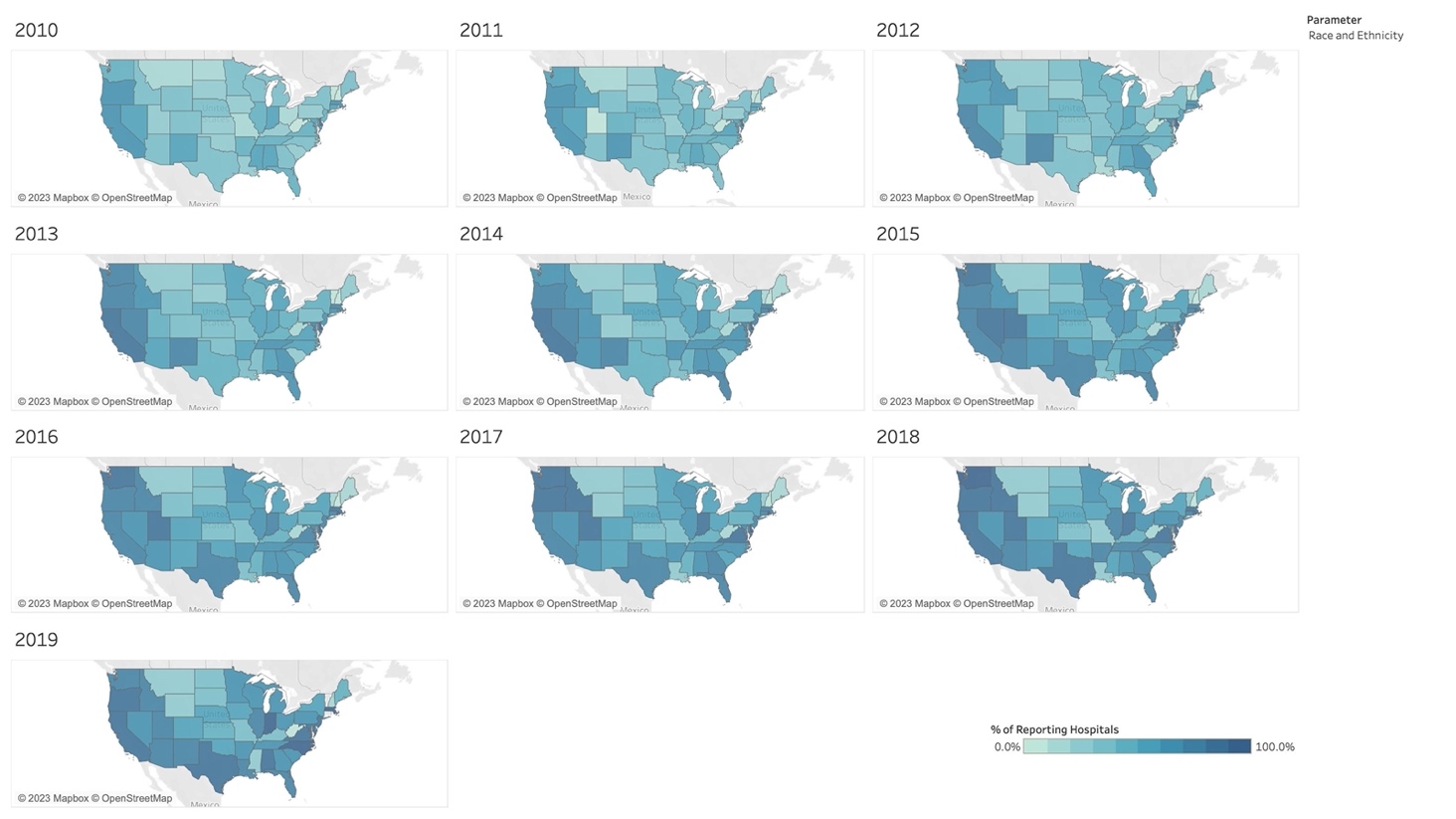


Percentage of hospital reporting entities by state with one or more uses of phrases in the *race and ethnicity* theme by year.

**sFigure 44. Exercise Theme**


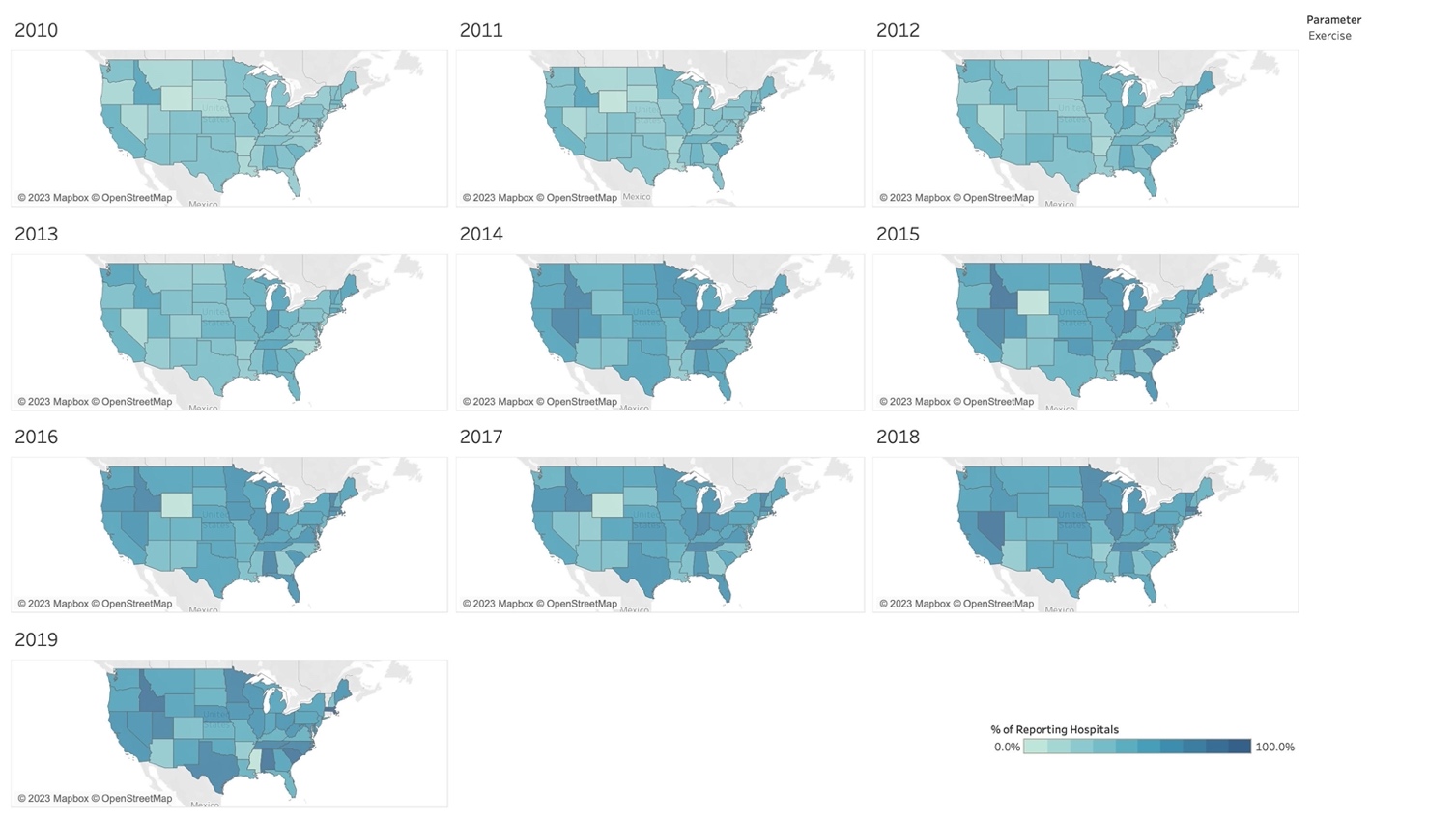


Percentage of hospital reporting entities by state with one or more uses of phrases in the *exercise* theme by year.

**sFigure 45. Rural Theme**

**
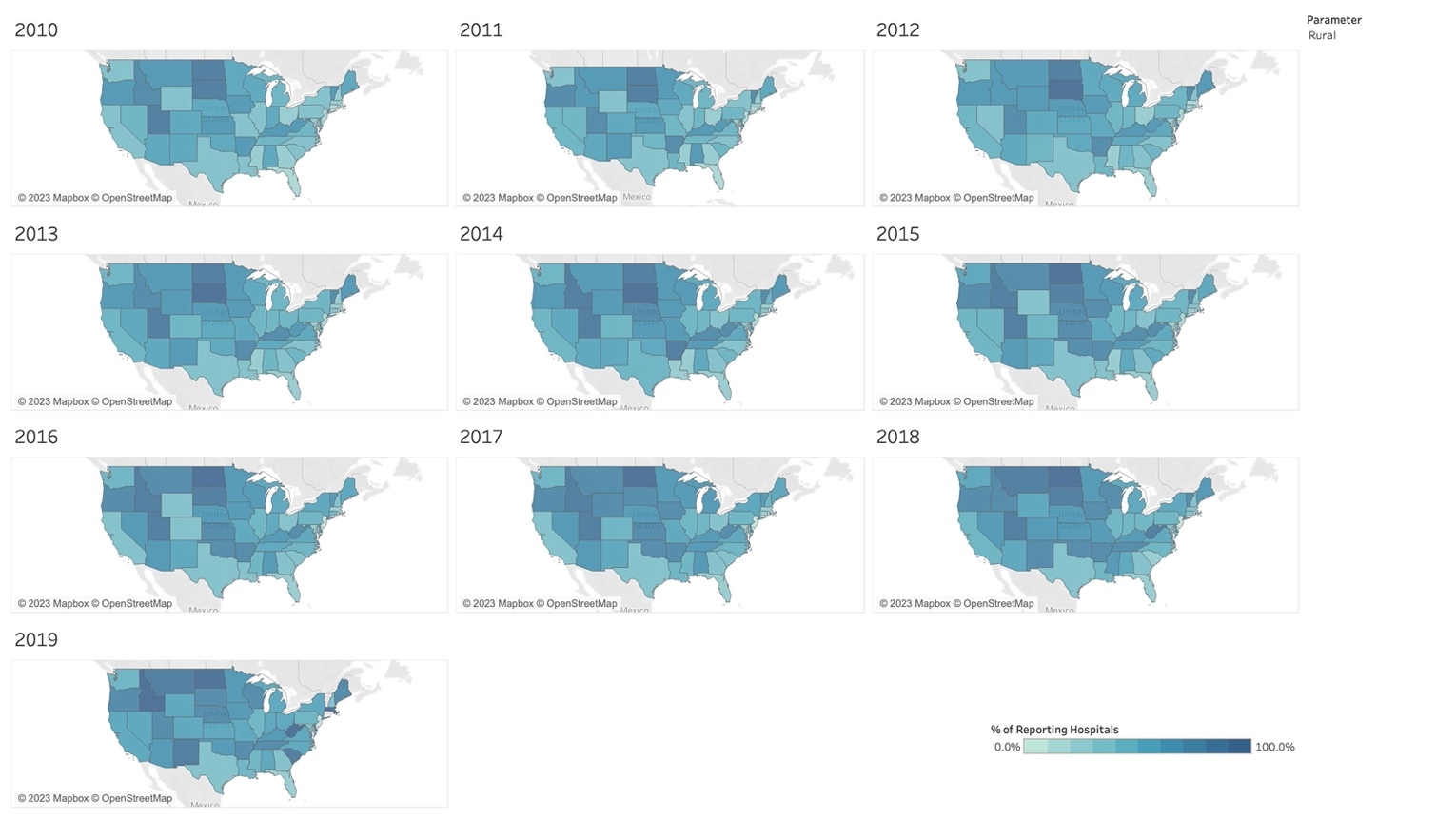
**

Percentage of hospital reporting entities by state with one or more uses of phrases in the *rural* theme by year.

**sFigure 46. Equity Theme**


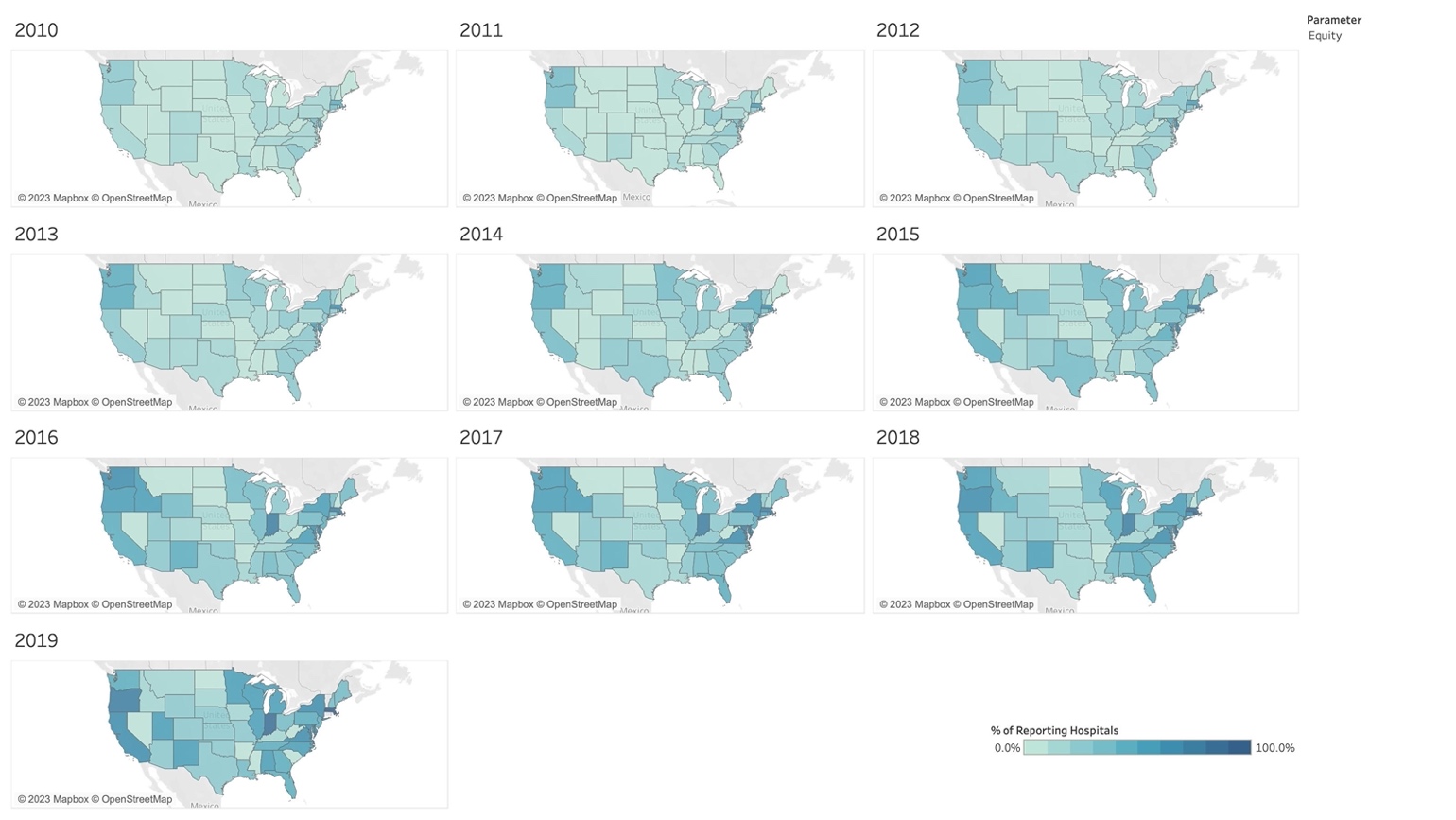


Percentage of hospital reporting entities by state with one or more uses of phrases in the *equity* theme by year.

**sFigure 47. Homelessness Theme**


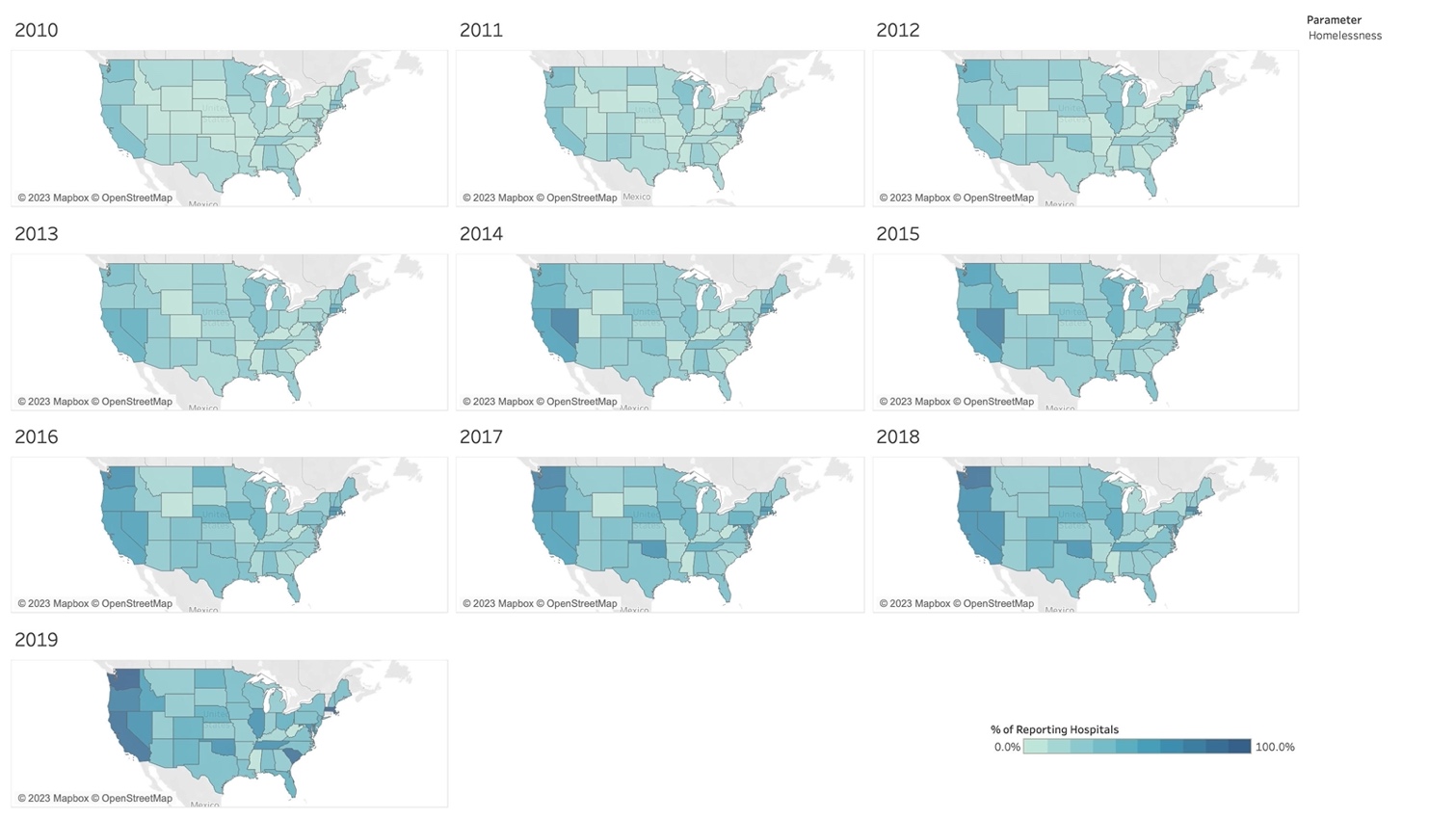


Percentage of hospital reporting entities by state with one or more uses of phrases in the *homelessness* theme by year.

**sFigure 48. Tobacco Theme**


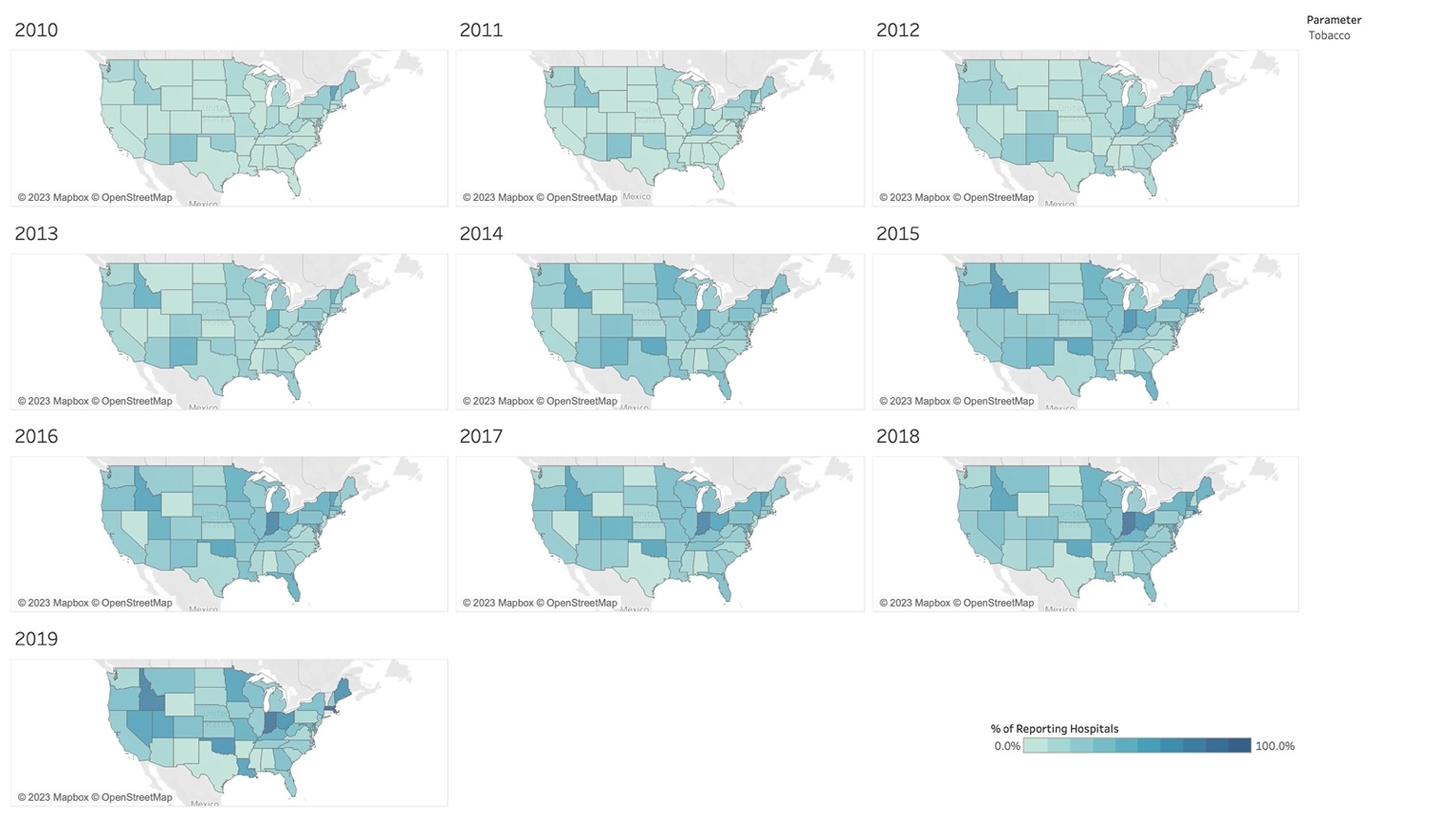


Percentage of hospital reporting entities by state with one or more uses of phrases in the *tobacco* theme by year

**sFigure 49. Criminal Justice Theme**


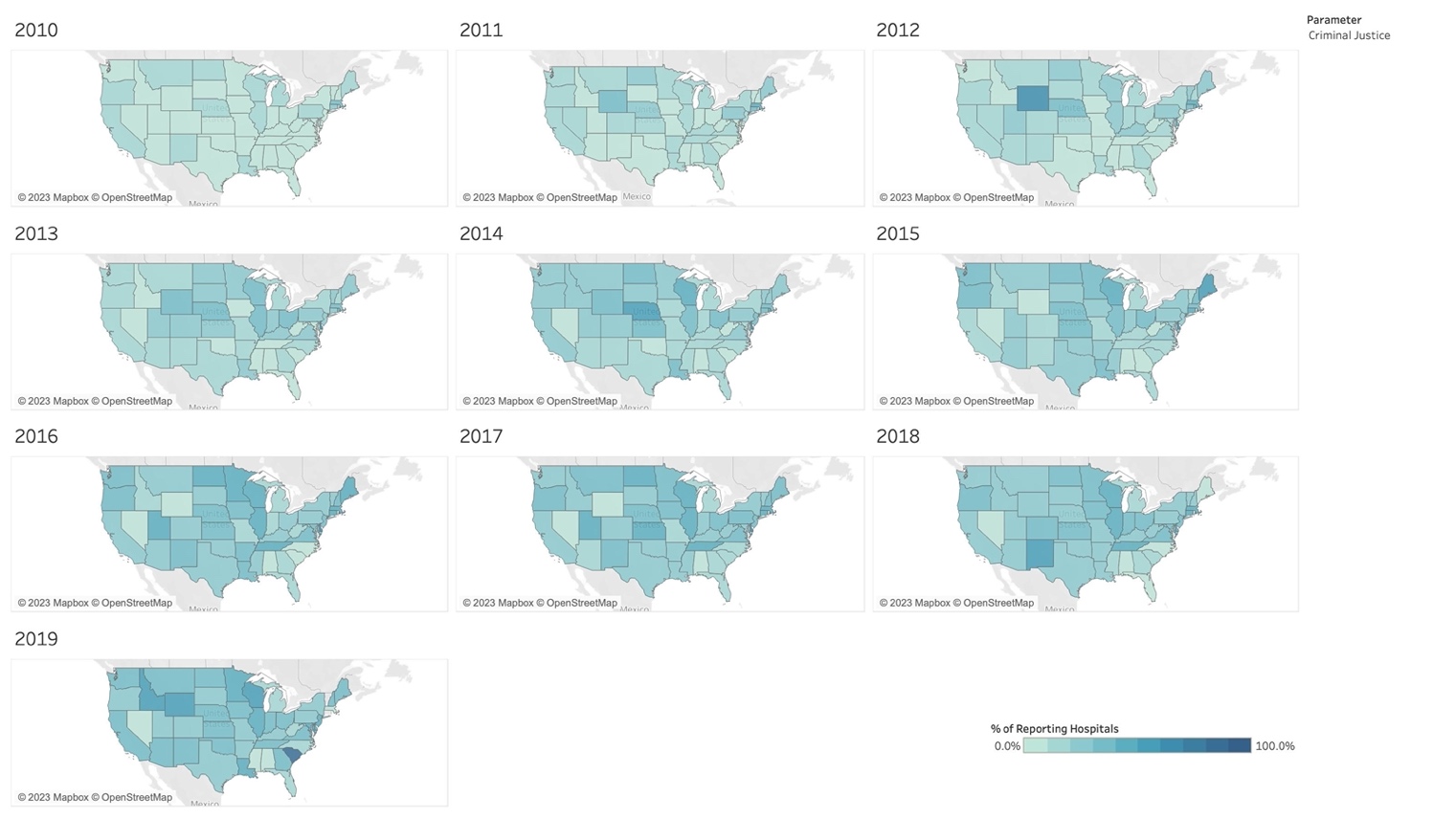


Percentage of hospital reporting entities by state with one or more uses of phrases in the *criminal justice* theme by year.

**sFigure 50. SDOH Theme**


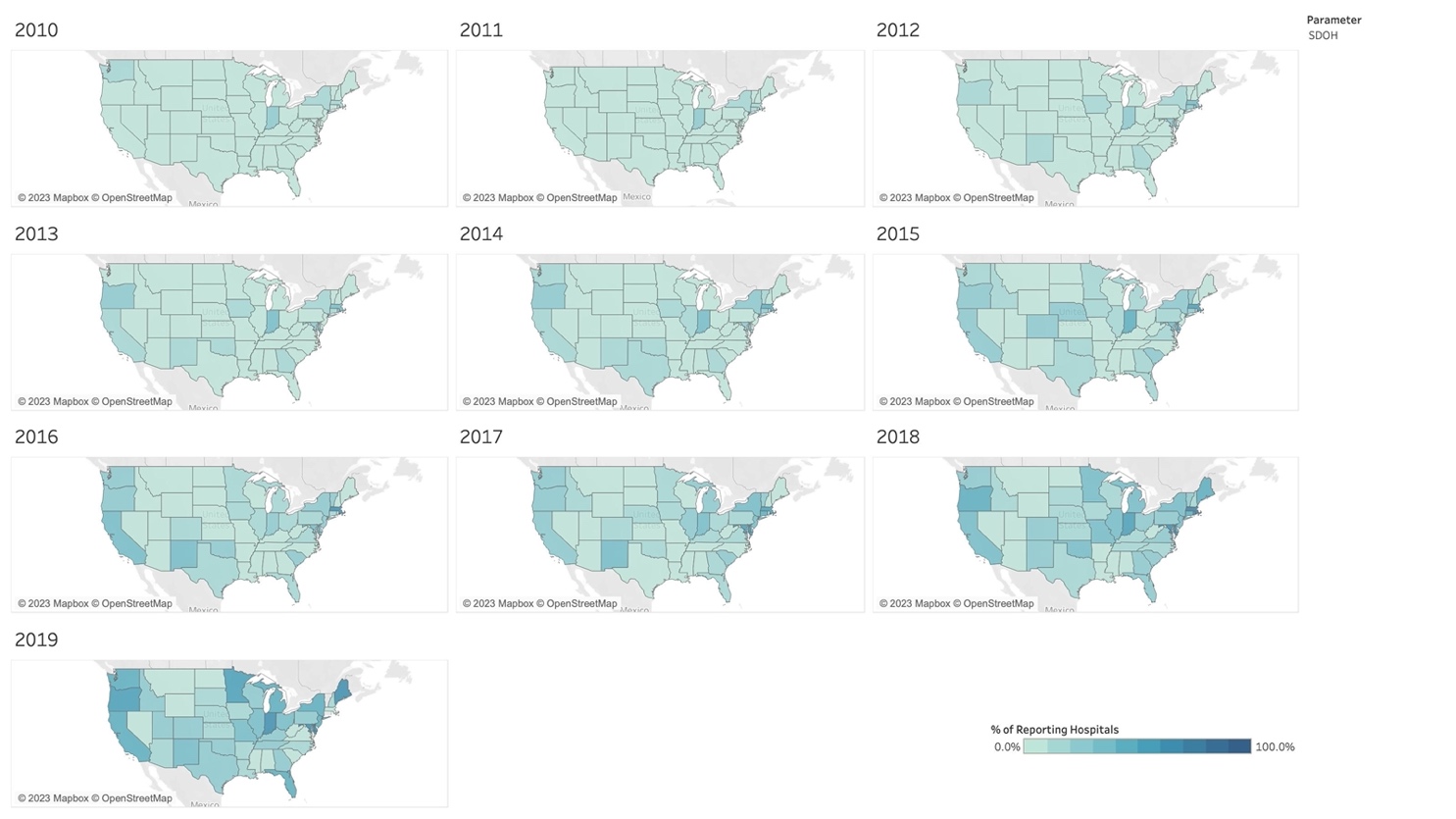


Percentage of hospital reporting entities by state with one or more uses of phrases in the *SDOH* theme by year.

**sFigure 51. Health IT Theme**


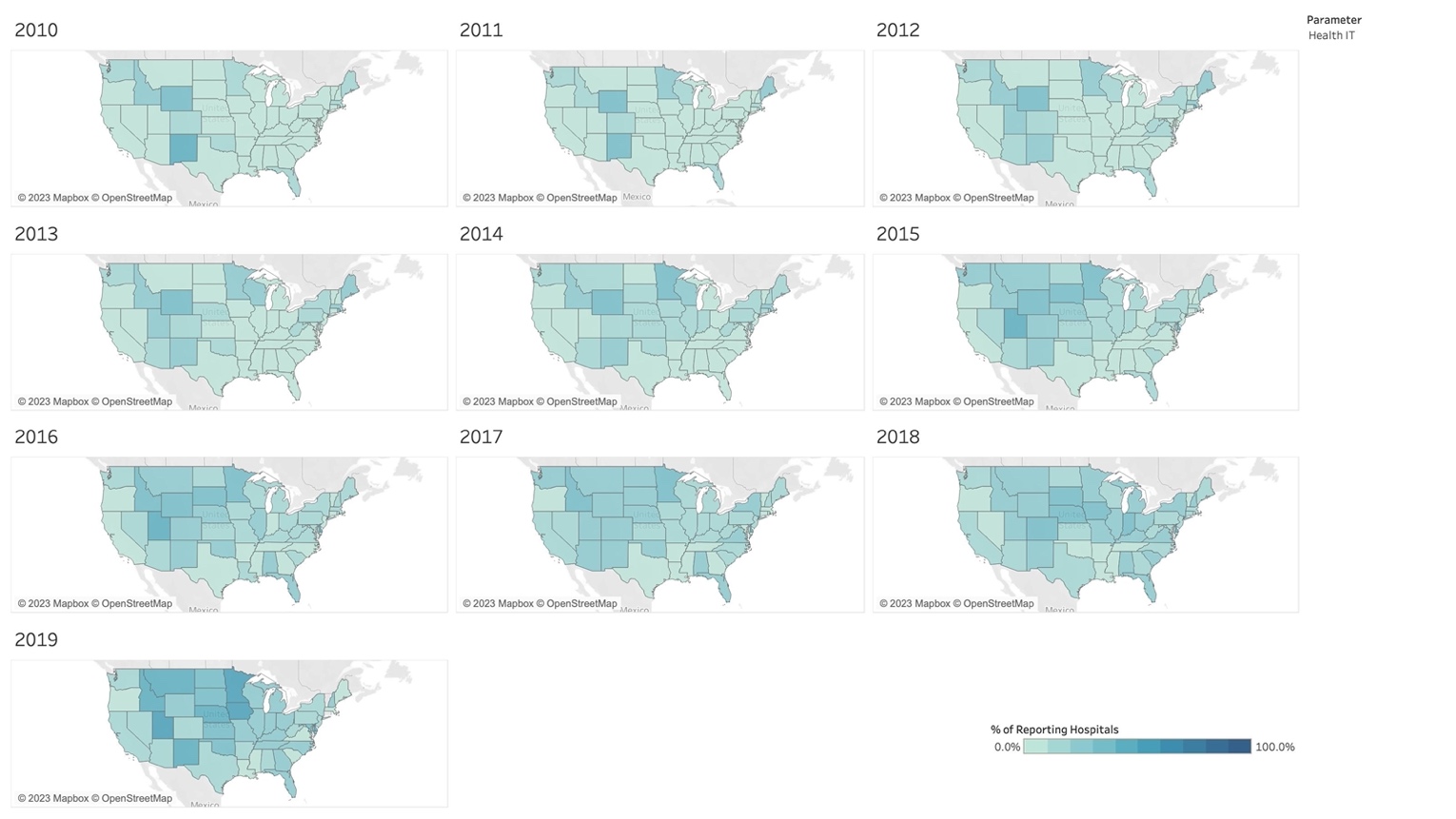


Percentage of hospital reporting entities by state with one or more uses of phrases in the *health IT* theme by year.

**sFigure 52. Sexual Health Theme**


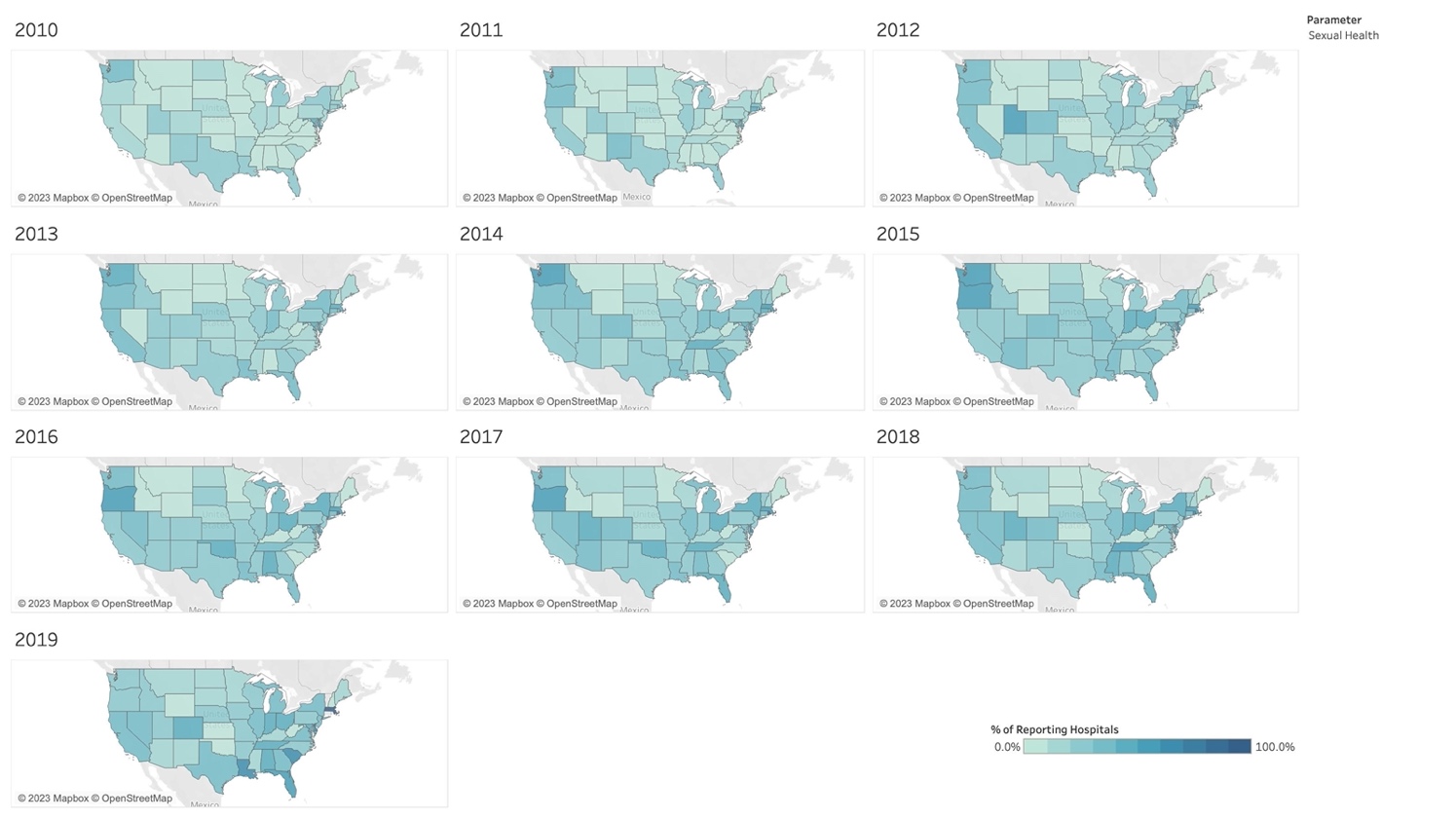


Percentage of hospital reporting entities by state with one or more uses of phrases in the *sexual health* theme by year.

**sFigure 53. Disability Theme**


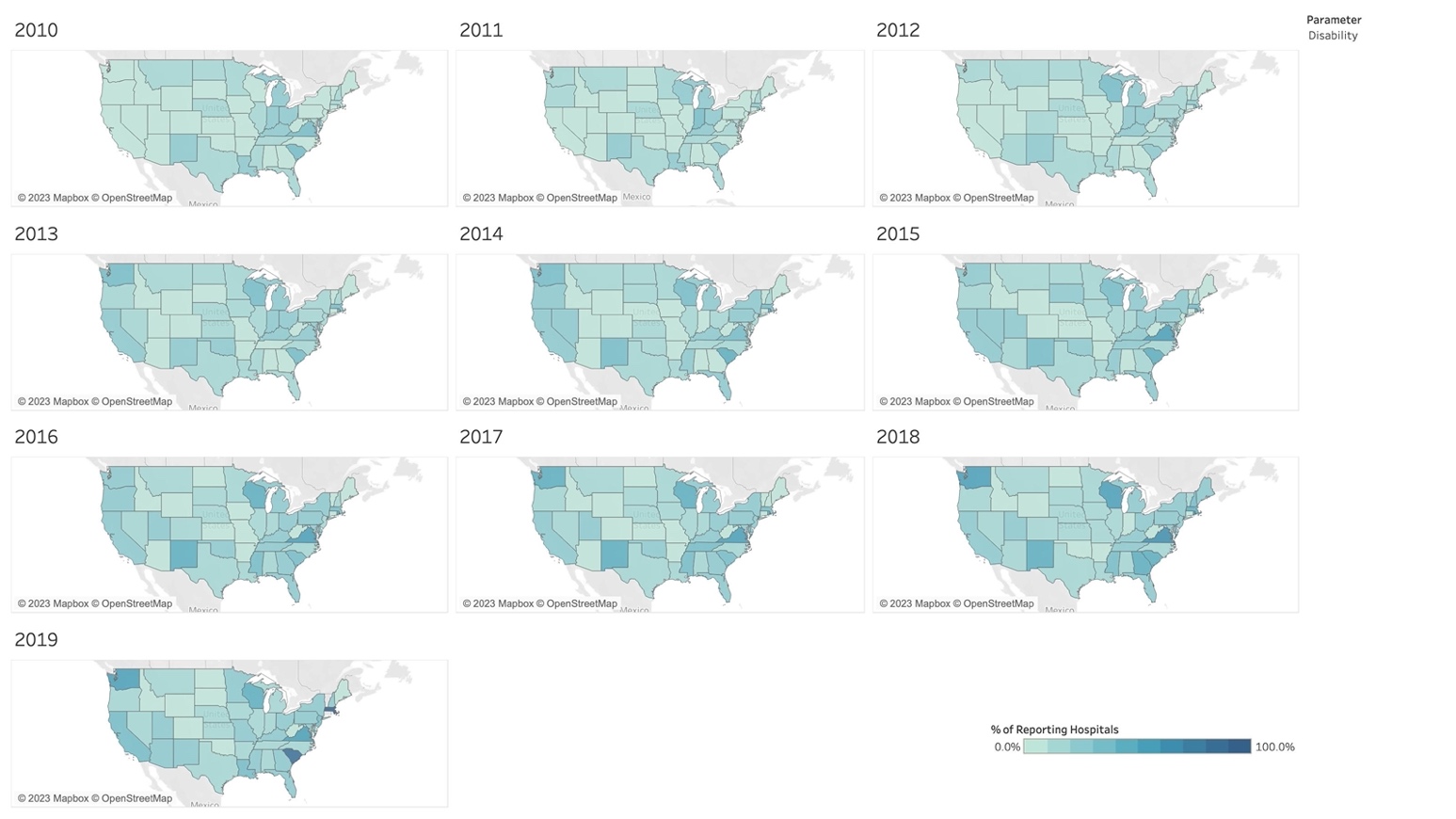


Percentage of hospital reporting entities by state with one or more uses of phrases in the *disability* theme by year.

**sFigure 54. Language Theme**


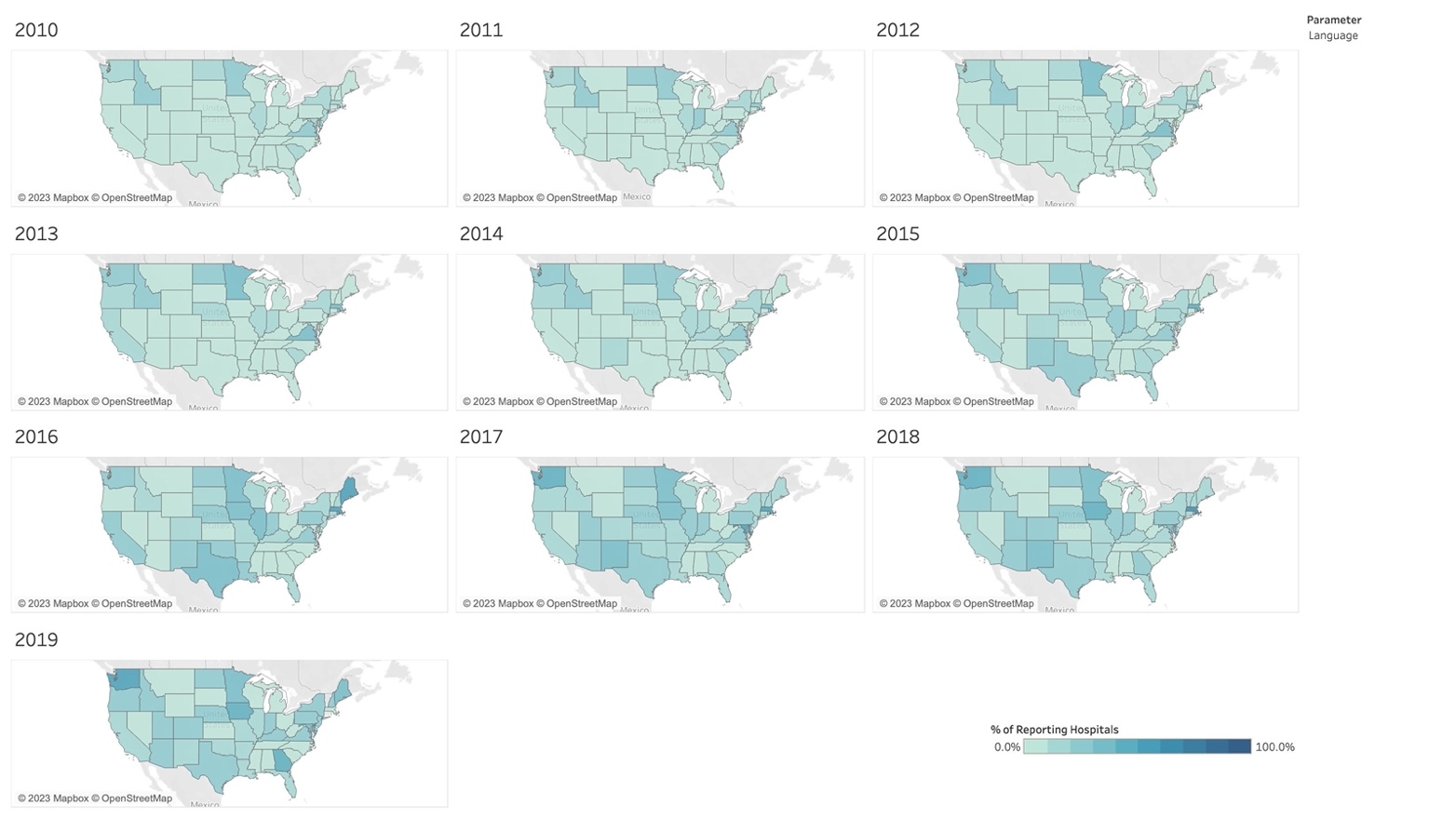


Percentage of hospital reporting entities by state with one or more uses of phrases in the *language* theme by year.

**sFigure 55. Firearms Theme**


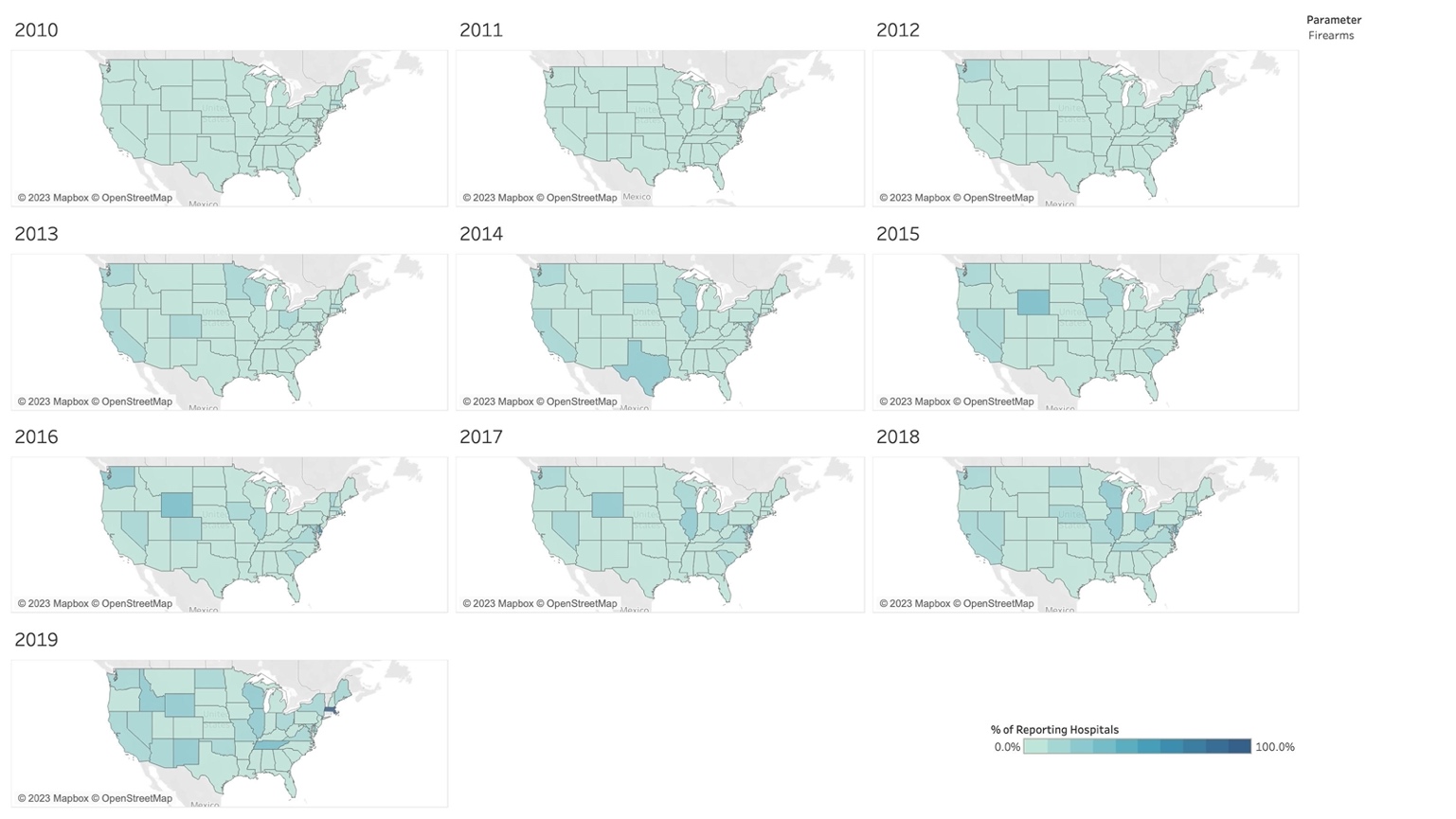


Percentage of hospital reporting entities by state with one or more uses of phrases in the *firearms* theme by year.

**sFigure 56. Immigration Theme**


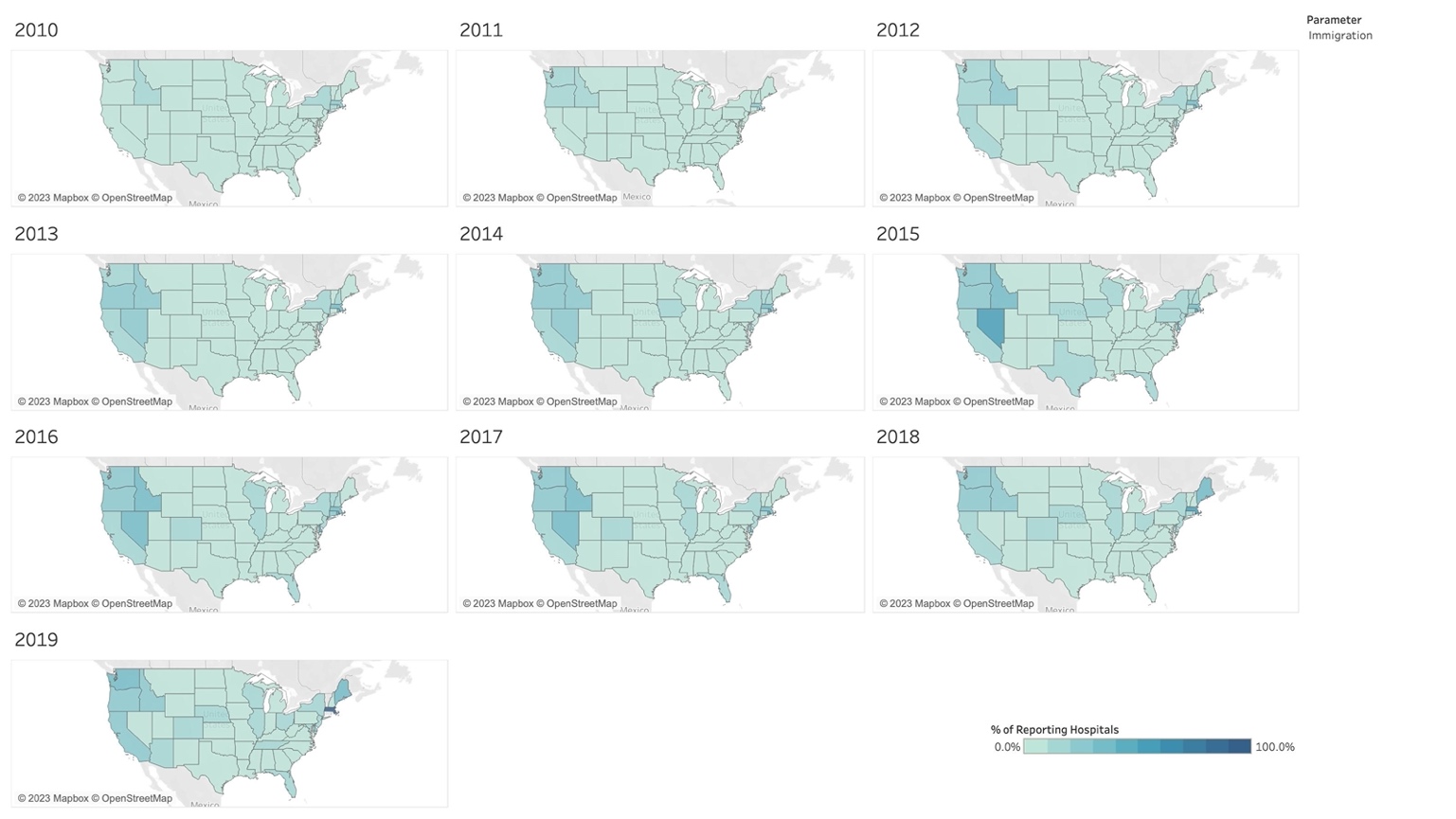


Percentage of hospital reporting entities by state with one or more uses of phrases in the *immigration* theme by year.

**sFigure 57. LGBTQ Theme**


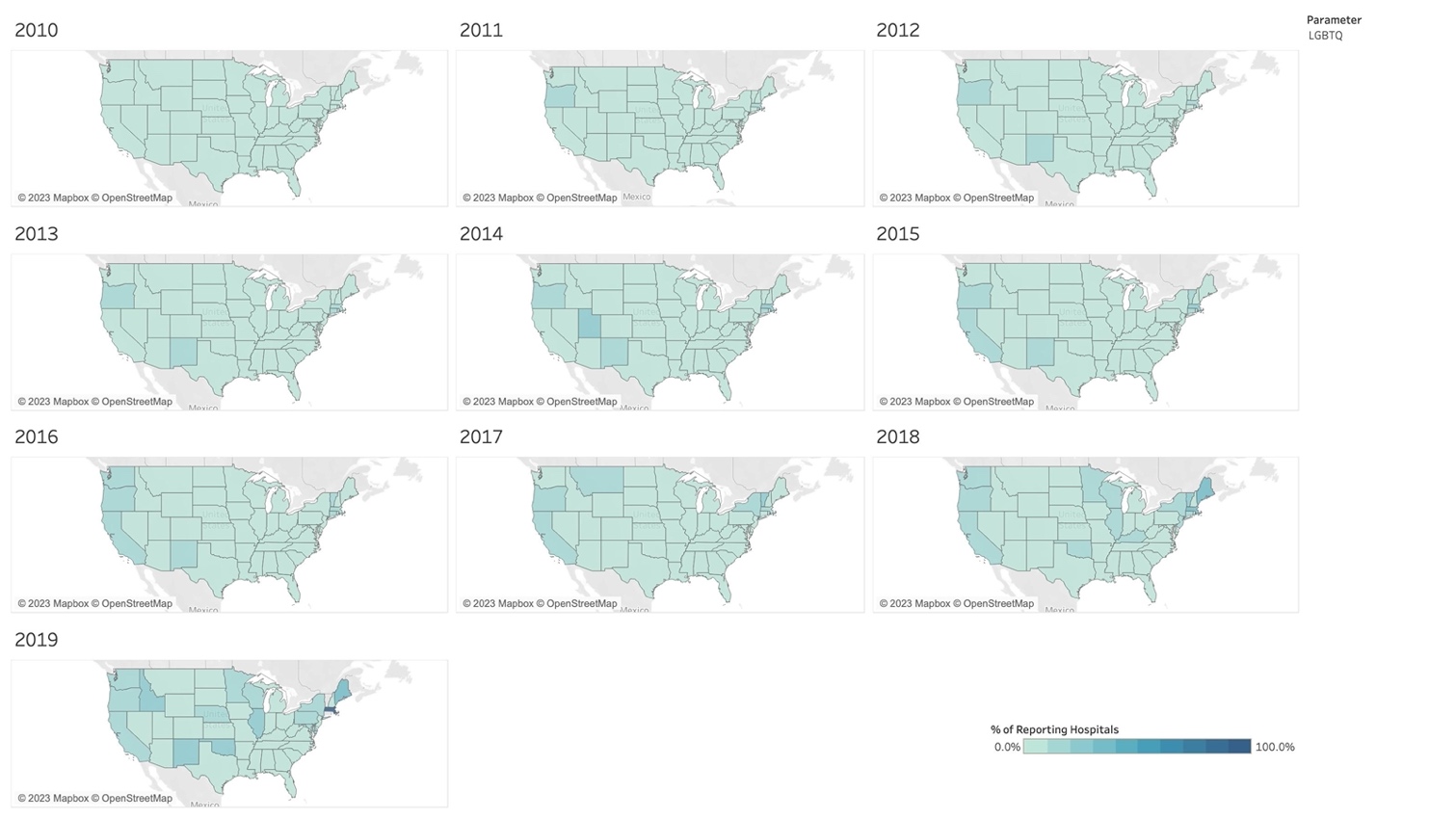


Percentage of hospital reporting entities by state with one or more uses of phrases in the *LGBTQ* theme by year.

**sFigure 58. Environment Theme**


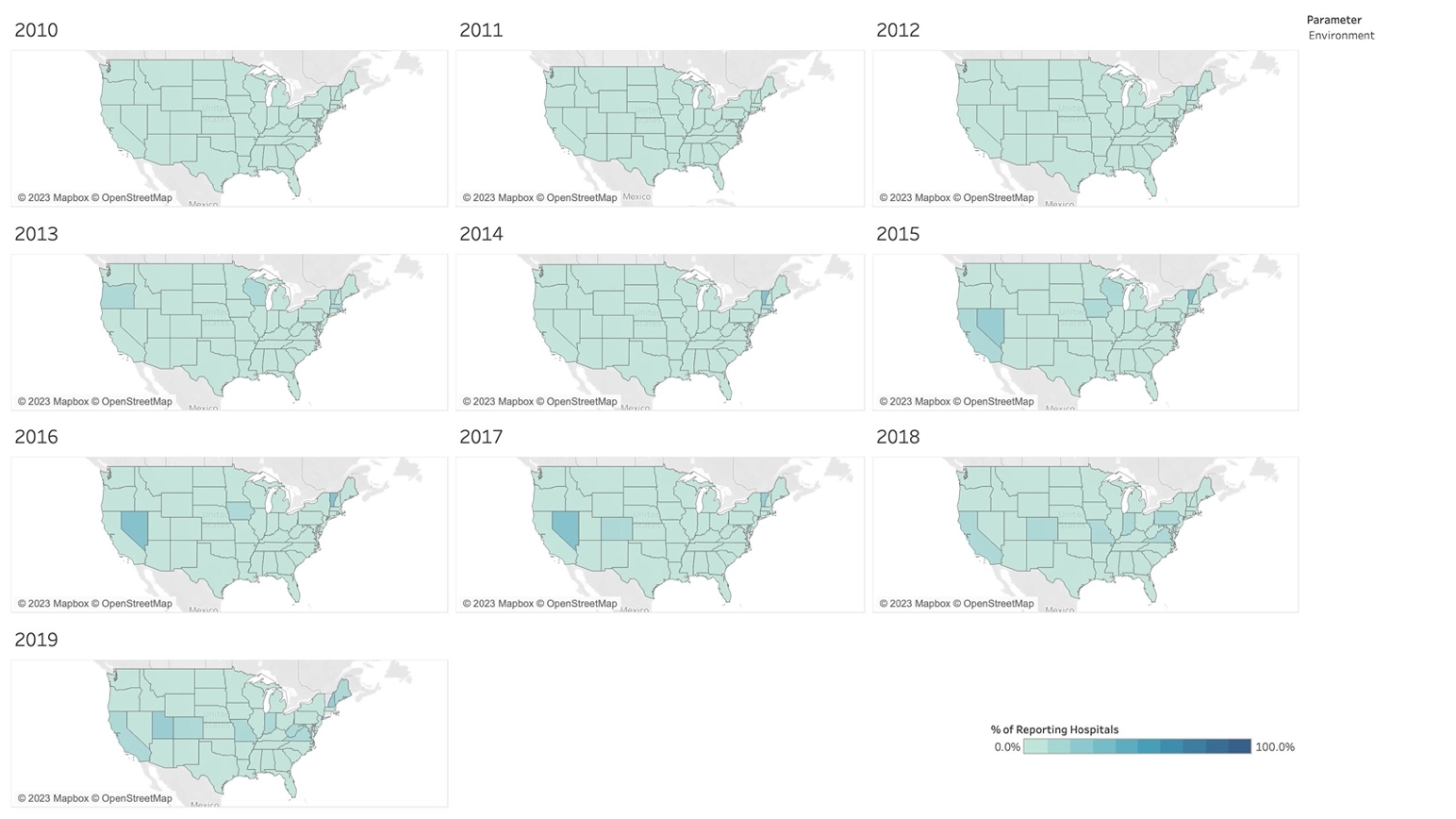


Percentage of hospital reporting entities by state with one or more uses of phrases in the *environment* theme by year.
